# Endocrine-disrupting chemicals and breast cancer: a meta-analysis

**DOI:** 10.3389/fonc.2023.1282651

**Published:** 2023-11-09

**Authors:** Haiyan Liu, Yukun Sun, Longkai Ran, Jiuling Li, Yafei Shi, Chunguang Mu, Changfu Hao

**Affiliations:** ^1^ Department of Child and Adolescent Health, School of Public Health, Zhengzhou University, Zhengzhou, Henan, China; ^2^ Department of Traditional Chinese Medicine, The First People’s Hospital of Guiyang, Guiyang, Guizhou, China; ^3^ Department of Clinical Nutrition, Jiaozuo Coal Industry (Group) Co. Ltd. Central Hospital, Jiaozuo, Henan, China

**Keywords:** endocrine-disrupting chemicals, breast cancer, epidemiological studies, pesticides, polychlorinated biphenyl, meta-analysis

## Abstract

**Background:**

Globally, the burden of breast cancer has increased significantly in recent decades. Emerging evidence suggested that endocrine-disrupting chemicals (EDCs), which have the potential to interfere with the function of normal hormones, may play a crucial role in this trend. However, the potential relationships were inconsistent in various studies.

**Objective and search methods:**

In our study, we sought to fully evaluate the currently available epidemiological evidence to ascertain whether certain EDC congeners and their metabolites are related to breast cancer risk. Following the Preferred Reporting Items for Systematic Reviews and Meta-Analyses guidelines, we conducted a comprehensive literature search of original peer-reviewed publications in three electronic databases: PubMed, Web of Science, and Embase. Publications that covered xenobiotic EDC exposures and breast cancer–confirmed histological results or antecedent medical records or reporting to health registers were taken into consideration.

**Outcomes:**

The final result of the literature search was 6,498 references, out which we found 67 publications that matched the requirements for meta-analysis and eight publications for qualitative trend synthesis. In this meta-analysis, statistically significant associations revealed that (i) 1-chloro-4-[2,2,2-trichloro-1-(4-chlorophenyl)ethyl]benzene (p,p'-DDT) and its major metabolite 2,2-bis(4-chlorophenyl)-1,1-dichloroethylene (p,p'-DDE) were somewhat related to a greater risk of breast cancer. However, this relationship only existed in blood serum but not in adipose tissue. (ii) Breast cancer risk was increased by exposure to chlordane and hexachlorocyclohexane. (iii) Five polychlorinated biphenyls (PCB 99, PCB 105, PCB 118, PCB 138, and PCB 183) can increase the risk of breast cancer. (iv) One phthalate congener (BBP) and one per- and polyfluoroalkyl substance congener (PFDoDA) were negatively associated with breast cancer risk. Unfortunately, heterogeneity was not well explained in our review, and a limited number of available prospective studies investigating the associations between EDC exposure and breast cancer were included in our meta-analysis. To elucidate the overall associations, future large, longitudinal epidemiological investigations are needed.

**Systematic review registration:**

https://www.crd.york.ac.uk/PROSPERO/, identifier CRD 42023420927.

## Introduction

The global burden of breast cancer is increasing significantly. According to GLOBOCAN 2020 (2021), an estimated of 2.3 million new breast cancer cases were diagnosed in 2020, which contributed to the most female cancer deaths globally (1). These numbers are expected to double by 2040, particularly in low- and middle-income countries (2). Epidemiological evidence has correlated different factors for the high incidence and death rates in breast cancer, such as obesity, late age for marriage, first childbirth, menopause, and early age at menarche. However, these factors only partially contributed to breast cancer risk (3). Recently, there has been an ongoing topic of debate regarding whether endocrine-disrupting chemicals (EDCs), which have evidence of being hormonally active, are partly attributed to breast cancer risk.

EDCs, which have the potential to interfere with the function of normal hormones and thus have a negative impact on an intact organism’s or its offspring’s health (4), are ubiquitous in the environment, and they can be widely absorbed by the human body through the skin, inhaled, and ingested. Although some EDC compounds have been banned in many countries, pollution still exists in the environment and in the food chain (5, 6). For example, dichloroethylene (DDT) and hexachlorocyclohexane (HCH), which were banned in 1983, are still detectable at considerable levels in some soils in China (7). EDC exposure is one stressor that might adversely affect normal human development. Adverse health outcomes, such as cardiovascular risk, autoimmune defects, male reproductive disorders, earlier timing of pubertal onset, and behavioral disorders are linked to EDC exposure (4, 8, 9). In addition, accumulating evidence has shown that the estrogenic properties of EDCs are potentially linked to the increasing rates of breast cancer. However, there is presently no consensus. In 2022, a systematic review, including 131 publications, identified that EDC exposure played a potential role in elevating the risk of breast cancer (10). However, no meta-analyses were conducted in this review. In light of recent epidemiological data, a meta-analysis study of the effects of environmental endocrine-disrupting xenobiotics on breast cancer has become necessary. In this meta-analysis, we conducted a comprehensive peer-reviewed of original literature search to obtain epidemiological evidence and analyzed whether 10 certain compound groups of common EDCs [bisphenol A (BPA), dioxins, parabens, phthalates diesters and their metabolites, flame retardants, polyaromatic hydrocarbons (PAHs), polychlorinated biphenyls (PCBs), organochloride pesticides, per- and polyfluoroalkyl substance (PFAS), and triclosan] and their metabolites (using biomarker measures) are related to breast cancer risk.

## Methods

### Protocol

This meta-analysis was carried out entirely in accordance with the protocol registered at PROSPERO.org (registration number CRD 42023420927) and Preferred Reporting Items for Systematic Reviews and Meta-Analyses (PRISMA) guidelines (11).

### Search strategy

The available research on EDC exposure and breast cancer was identified through a comprehensive peer-reviewed of original literature search in three electronic databases, namely, PubMed, Web of Science, and Embase, from 1961 to May 2023. The identified search terms were divided into three search blocks: the first dealt with the EDC exposure, the second covered the outcome (breast cancer), and the last covered study design (case−control and cohort study). A manual search of the included article’s reference lists was subsequently performed. The search protocol provided the search specifications and respective hits in each search block (**Supplementary Table 1**).

### Inclusion criteria

Original research papers published in English were included in our analysis. The full text of the corresponding article was reviewed after the title and abstract had been evaluated. Publications were considered eligible for inclusion if they met all the below criteria.

1. Exposures: Exposures to certain EDCs documented by measurements in biological specimens (blood, urine, and adipose tissue) were included in the meta-analysis. The following 10 compound groups of EDCs were investigated in the included publications: (i) BPA, (ii) dioxins, (iii) parabens, (iv) phthalates diesters and their metabolites, (v) flame retardants, (vi) PAHs, (vii) PCBs, (viii) organochloride pesticides, (ix) PFAS, and (x) triclosan.
2. Breast cancer: Breast cancer–confirmed histological results or antecedent medical, records, or reporting to health registers were taken into consideration.
3. Risk estimates [relative risk (RR), odds ratio (OR), and hazard ratio (HR)] and their 95% confidence intervals (95% CI) as an outcome according to higher versus lower levels of EDC exposure contacts within the given study.
4. Only cohort studies and case−control studies were included in our analysis.

### Exclusion criteria

Criteria for exclusion of studies were as follows:

1. Research studies conducted on animals, case reports, cross-sectional researches, reviews, conference proceedings/abstracts, letters, editorials, and comments were not included in our analysis.
2. Publications that discussed prescription hormones, did not report risk estimates, or reported repeated estimates from other publications were excluded.
3. Publications that reported DDT, PCBs, PFAS, and phthalate congener summary estimates but no specific risk estimates were excluded.
4. Self-reported breast cancer was excluded.

### Data extraction

The process of data extraction was performed independently by KR and YS, and any inconsistency was resolved by JL. The following information was extracted from each publication including author, location, study design, number of cases and referents, biospecimens, exposure contrast, and substance. Risk estimates with 95% CIs were recorded for each measured compound. When risks according to several levels of exposure were reported, The risk estimate of the highest versus lowest levels was chosen. If a study reported that the OR value was in both unadjusted and adjusted models, then we gave preference to the adjusted OR value.

### Statistical analysis

Studies were eligible in the meta-analysis when the effect sizes were reported as an relative risk (OR, RR, and HR) and sample types were human specimens. Separate forest plots for each EDC exposure were conducted to illustrate summary ORs with 95% CIs. The random-effects model was used to summarize the risk estimates. A meta-analysis was performed independently when ≥3 studies reported the compound. Heterogeneity was assessed using the degree of I^2^-test statistic and *p*-value. Significant heterogeneity was defined as I^2^ > 50% or value of *p* < 0.10. Low, moderate, and high degrees of heterogeneity were defined with I^2^-values of 25%, 50%, and 75% (12). Subgroup analysis was used to determine the source of heterogeneity when it was assessed as moderate or high degree. We stratified our analysis into categories on the basis of the study design (case−control and nested case−control) and sample type (blood, adipose tissue, or urine). The leave-one-out method was used to perform sensitivity analysis. All statistical analyses of the data were performed using STATA software (version 15.0; State Corporation, College Station, Texas, USA) with a significance level of 0.05.

### Risk of bias and quality assessment

The process of risk of bias and quality assessment was performed independently by two authors (KR and YS), and any inconsistency was resolved by a third author (JL). Each study was assessed for the completeness of reporting using a standardized form adapted from (9). There are a total of 11 items that need to be evaluated, and the 11 areas were equally weighted with the value one given for adequate reporting. We deemed a total of 8 to be adequate for reporting completion. A standardized questionnaire that was derived from (13) was used to assess the potential sources of bias in each study. There are a total of seven items, including reporting of tested hypotheses, sample size justification, selection bias, information bias, confounding, measuring of confounding factors, and exposure contrast, that need to be evaluated and each area was either rated as high risk, uncertain risk, or low risk (the evaluation form is available in **Supplementary Table 11**). If two or more of the specified areas were found to carry a high risk of bias, then publications were deemed to be biased in that direction. The potential sources of bias are showed in **Supplementary Table 12**.

## Results

### Study selection and characteristics

**Figure 1:**
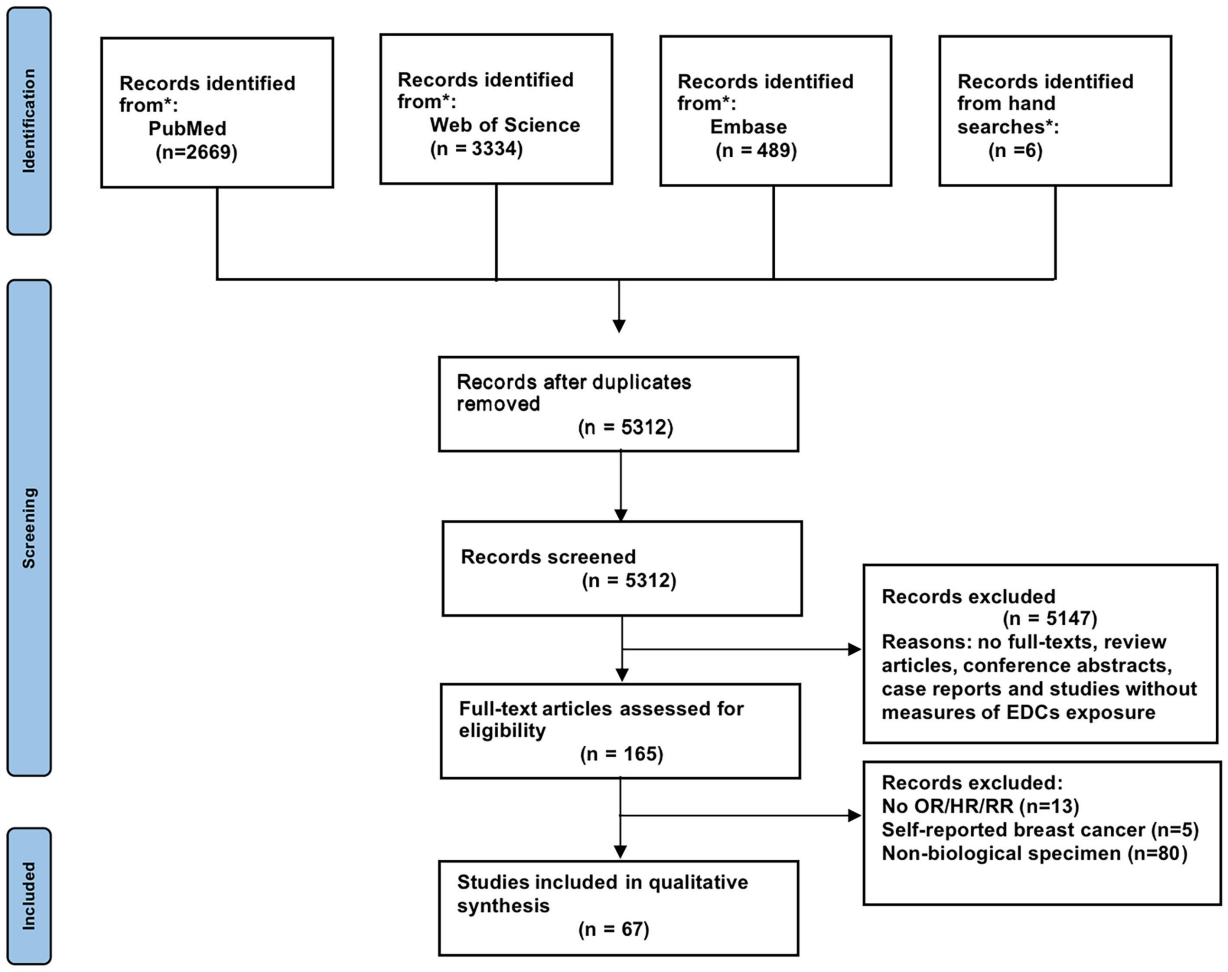
Flow diagram according to the Preferred Reporting Items for Systematic Reviews and Meta-Analyses (PRISMA) protocol recommendations.

**Figure 1** depicts the screening and selection procedure for the study. A total of 6,498 records were retrieved from PubMed, Web of Science, and Embase, of which 1,186 were duplications. Another 5,147 were disqualified when the titles and abstracts were examined because these studies were review articles, conference abstracts, or case reports or without measures of EDC exposure. No full-text studies were also disqualified. The full texts of 165 articles were reviewed after reading the titles and abstracts. Finally, 67 publications met the qualifying requirements for meta-analysis and eight publications for qualitative trend synthesis. All included studies concerned breast cancer only in women. **Supplementary Tables 2–10** provide an overview of the characteristics of the included publications.

### Pesticides

The relationship between pesticides and breast cancer has received the most attention because of their persistence in the environment (14). However, the results were inconsistent in various studies. In this meta-analysis, we included 38 publications addressing pesticides. The characteristics of the studies in our review are shown in **Supplementary Tables 2–5**.

### DDT and breast cancer

**Figure 2:**
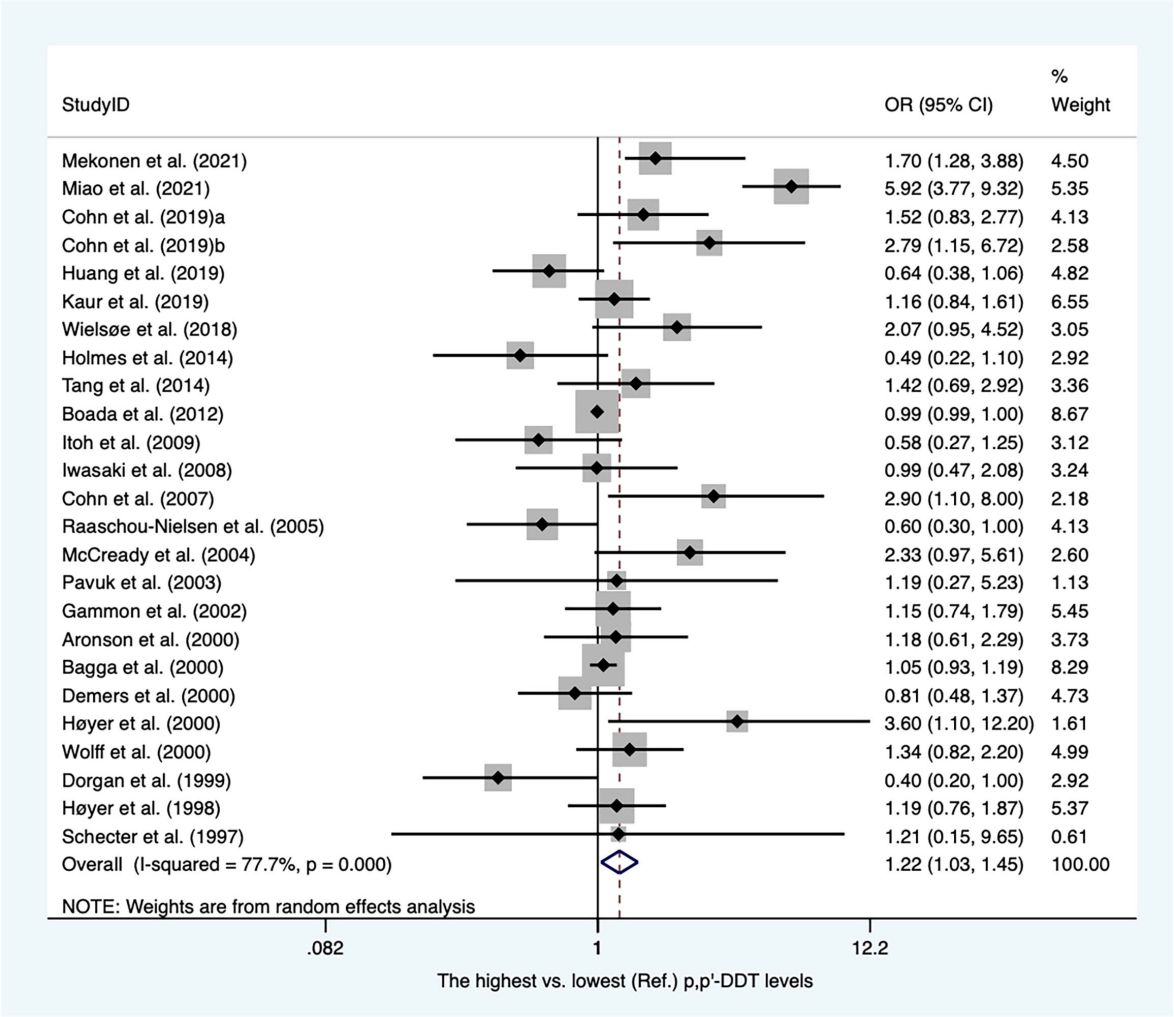
Summary estimates of the meta-analysis: association between p,p′-DDT exposure and breast cancer.

**Figure 3:**
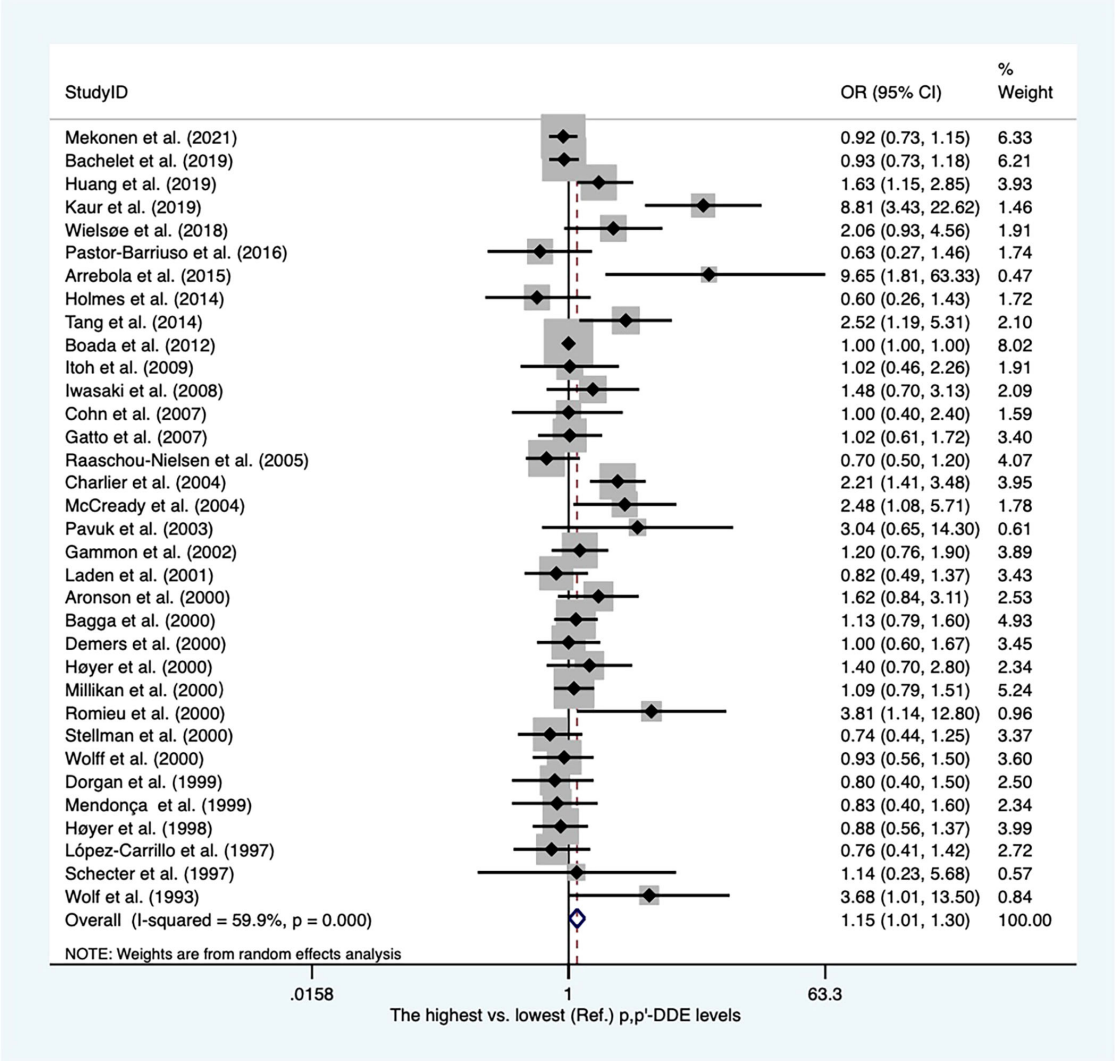
Summary estimates of the meta-analysis: association between p,p′-DDE exposure and breast cancer.

There were twenty-eight case−control articles and eight nested case−control studies (**Supplementary Table 2**). Of these, six publications reported DDT levels from adipose tissues, whereas others presented concentrations of DDT from blood samples. Thirty-six case-referent studies provided thirty-four risk estimates for p,p′-DDE, twenty-five risk estimates for p,p′-DDT, four risk estimates for o,p′-DDT, and four risk estimates for p,p′-DDD. The summary OR based on twenty-four studies showed that there was a positive association between p,p′-DDT and breast cancer (OR, 1.22; 95% CI, 1.03–1.45) with high heterogeneity (I^2 ^= 77.7%, *P* < 0.001) (**Figure 2**). In subgroups stratified by study design and sample type, the OR for case−control studies was close to unity but not statistically significant (OR, 1.22; 95% CI, 1.00–1.49; I^2 ^= 81.7%; *P* < 0.001), whereas the blood serum p,p′-DDT was associated with an increase in breast cancer (OR, 1.32; 95%CI, 1.03-1.70; I^2 ^= 80.5%; *P* < 0.001) (**Supplementary Figures 1**, **2**). The pooled OR found that p,p′-DDE was associated with a significant increase in breast cancer (OR, 1.15; 95% CI, 1.01–1.30) with high heterogeneity between them (I^2 ^= 59.9%, *P* < 0.001) (**Figure 3**). In subgroups stratified by study design and sample type, the OR for case−control studies was 1.17 (95% CI, 1.02–1.34; I^2 ^= 63.4; *P* < 0.001), and the OR for blood serum was close to unity but not significant (OR, 1.15; 95% CI, 1.00–1.32; I^2 ^= 59.7%; *P* < 0.001) (**Supplementary Figures 3, 4**). There were only four studies that addressed o,p'-DDT in blood and an inverse association was observed in the meta-analysis (OR, 0.62; 95% CI, 0.42–0.92; I^2 ^= 5.6%; *P* = 0.365) (**Supplementary Figure 5**). The summary OR for p,p′-DDD in blood was slightly elevated but not statistically significant (OR, 2.78; 95% CI, 0.62–12.41; I^2 ^= 97.6%; *P* < 0.001) (**Supplementary Figure 6**).

### Hexachlorobenzene and breast cancer

**Figure 4:**
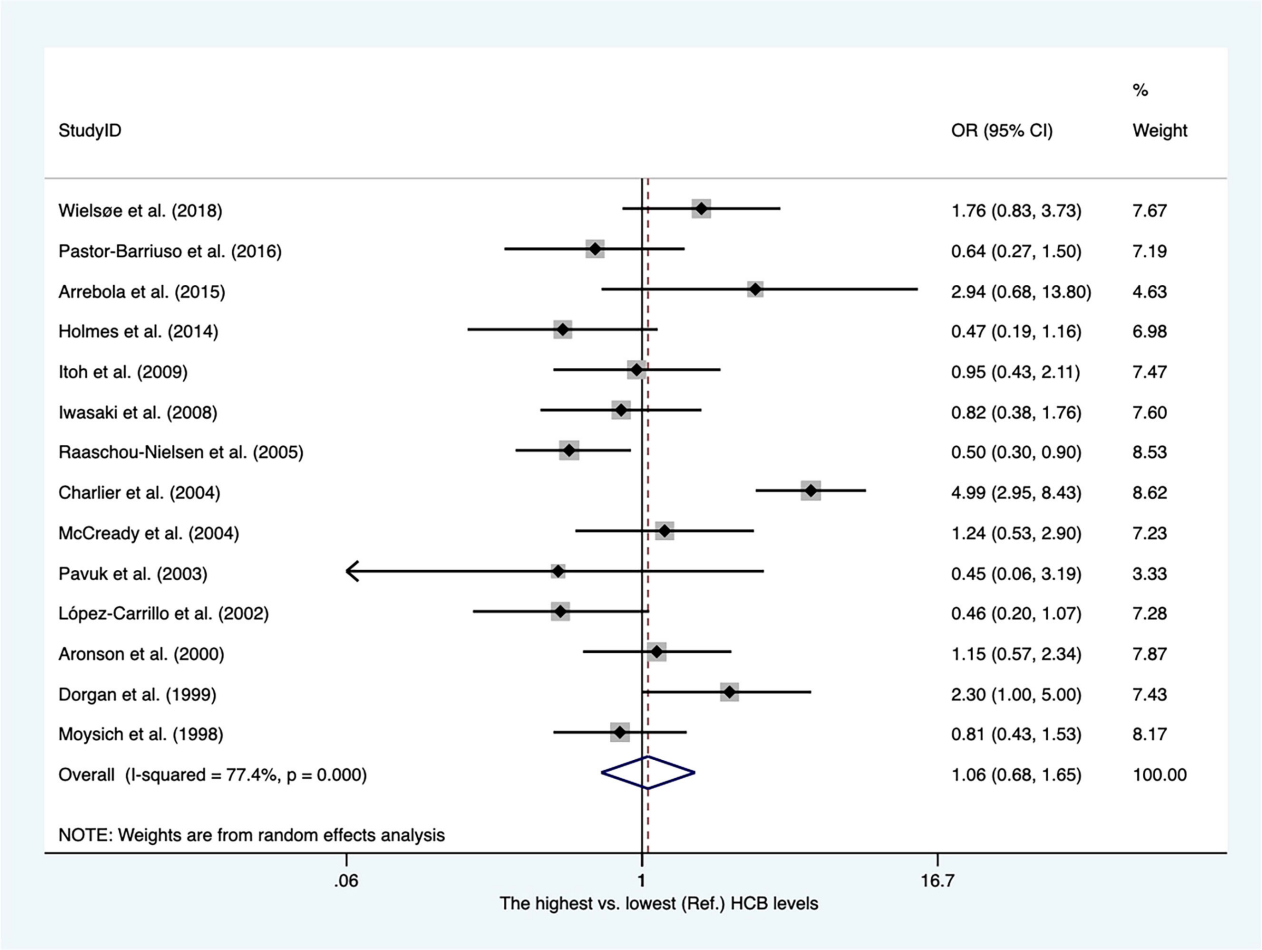
Summary estimates of the meta-analysis: association between HCB exposure and breast cancer.

There were twelve case−control articles and two nested case−control studies (**Supplementary Table 3**). Of these, three publications reported hexachlorobenzene (HCB) levels from adipose tissues, whereas others presented concentrations of HCB from blood samples. As shown in **Figure 4**, the overall OR for the highest vs. lowest HCB levels was 1.06 (95% CI, 0.68–1.65), with high heterogeneity among these studies (I^2 ^= 77.4%). The heterogeneity was not affected by subgroups of sample type (**Supplementary Figure 7**).

### Hexachlorocyclohexane and breast cancer

**Figure 5:**
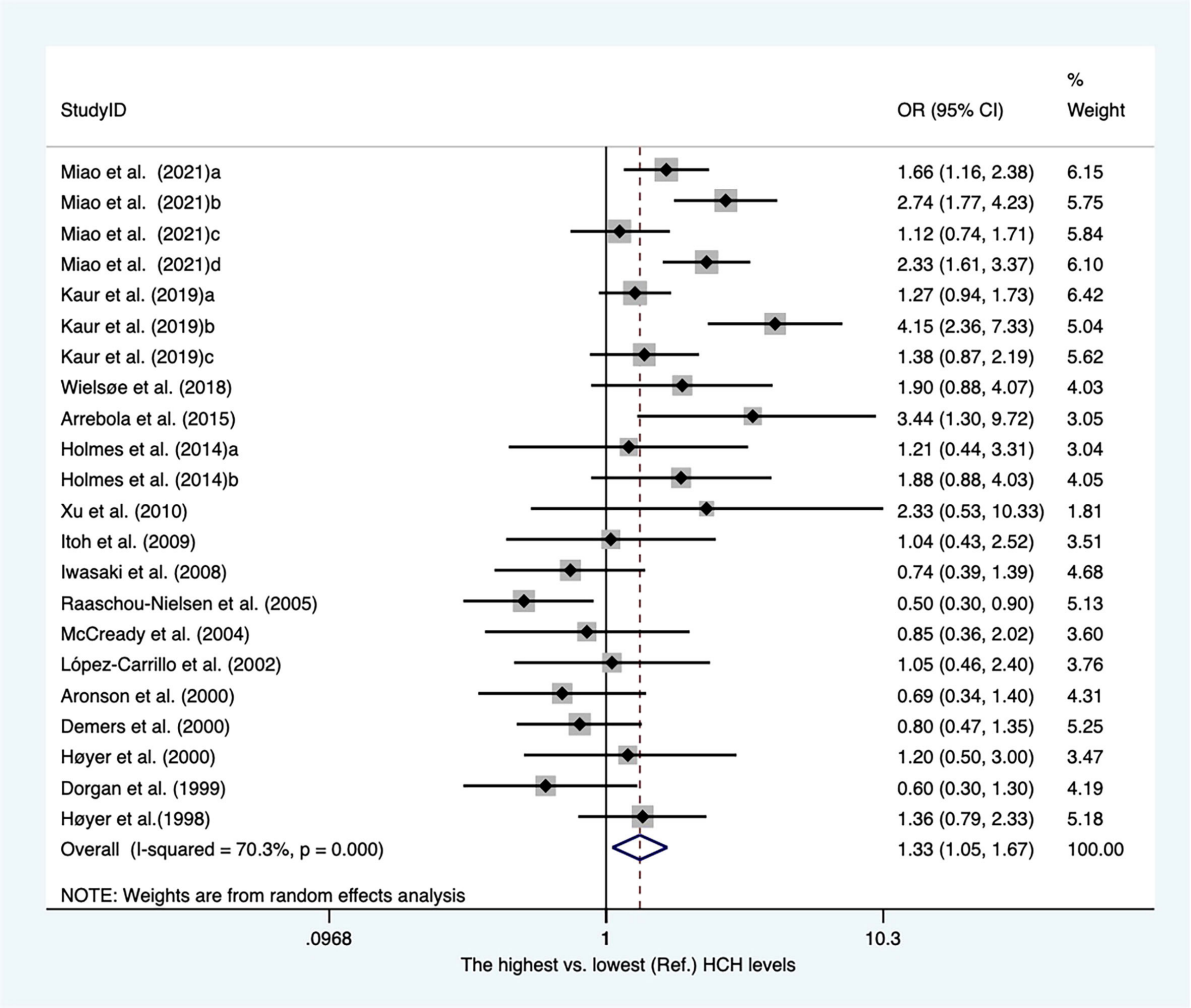
Summary estimates of the meta-analysis: association between HCH exposure and breast cancer.

Sixteen case-referent studies provided twenty-two risk estimates for hexachlorocyclohexane (HCH). There were 12 case−control articles and four nested case−control studies (**Supplementary Table 4**). Of these, three publications reported HCH levels from adipose tissues, whereas others presented concentrations of HCH from blood samples. As shown in **Figure 5**. The pooled OR showed that higher blood/fat levels of HCH was associated with a substantial increase in the risk of breast cancer in individuals (OR, 1.33; 95% CI, 1.05–1.67; I^2 ^= 70.3%; *P* < 0.001). The heterogeneity was affected by subgroups of sample type and study design. In blood serum, the concentrations of HCH were associated with a significant increase in breast cancer (OR, 1.48; 95% CI, 1.19–1.86; I^2^ = 64.2%; P < 0.001). The summary OR for HCH in adipose tissue was significantly reduced (OR, 0.61; 95% CI, 0.42–0.90) with no heterogeneity (**Supplementary Figure 8**). The summary estimate risk of twelve case−control publications was a statistically significant increase (OR, 1.47; 95% CI, 1.15–1.87; I^2 ^= 67%; P < 0.001) (**Supplementary Figure 9**).

### Other pesticides

**Figure 6:**
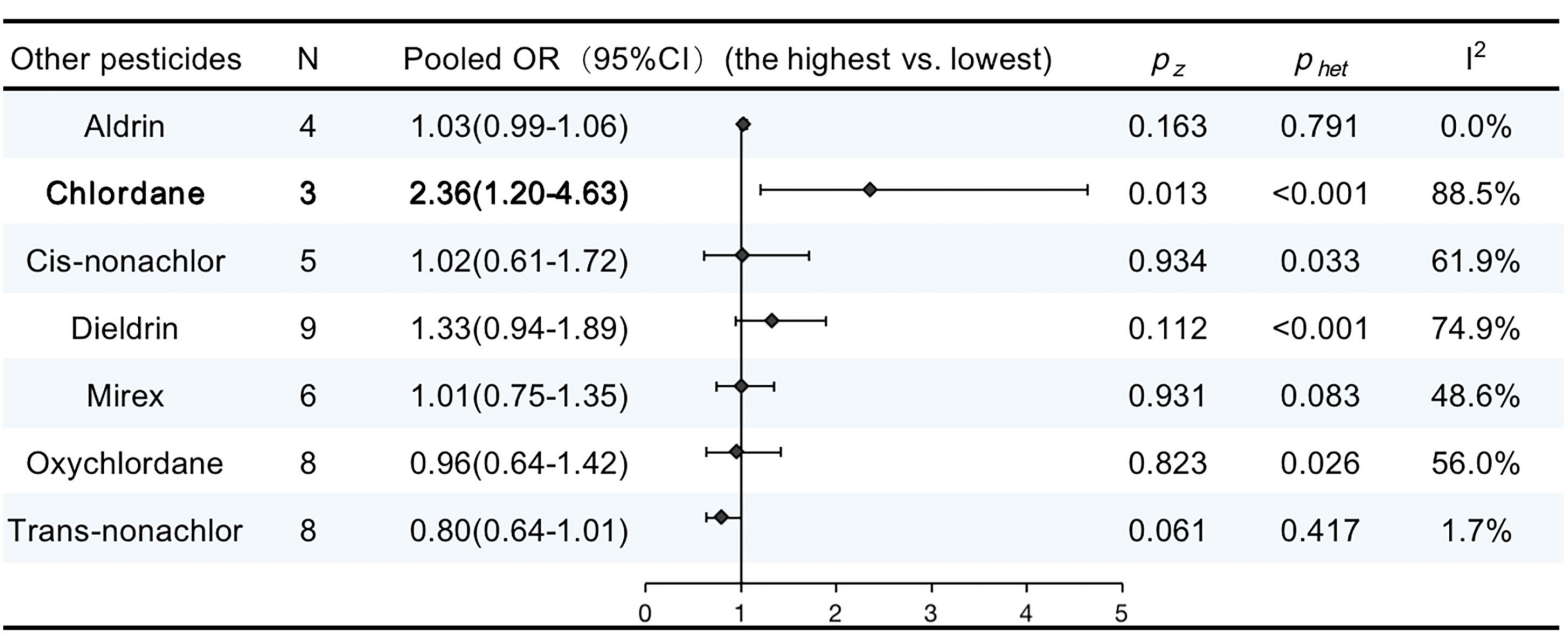
ORs (95% CI) of the summary estimate of analyses for associations between other pesticides and breast cancer.

There were 17 publications that reported the associations between other pesticide exposure and breast cancer. **Supplementary Table 5** details the features of the studies included in our meta-analysis. The pooled OR for chlordane showed a significant increase in the risk of breast cancer (OR, 2.36; 95% CI, 1.20–4.63; I^2 ^= 88.5%; *P* < 0.001). No significant increase were observed in other pooled ORs. The results are summarized in **Figure 6**.

### PCBs and breast cancer

**Figure 7:**
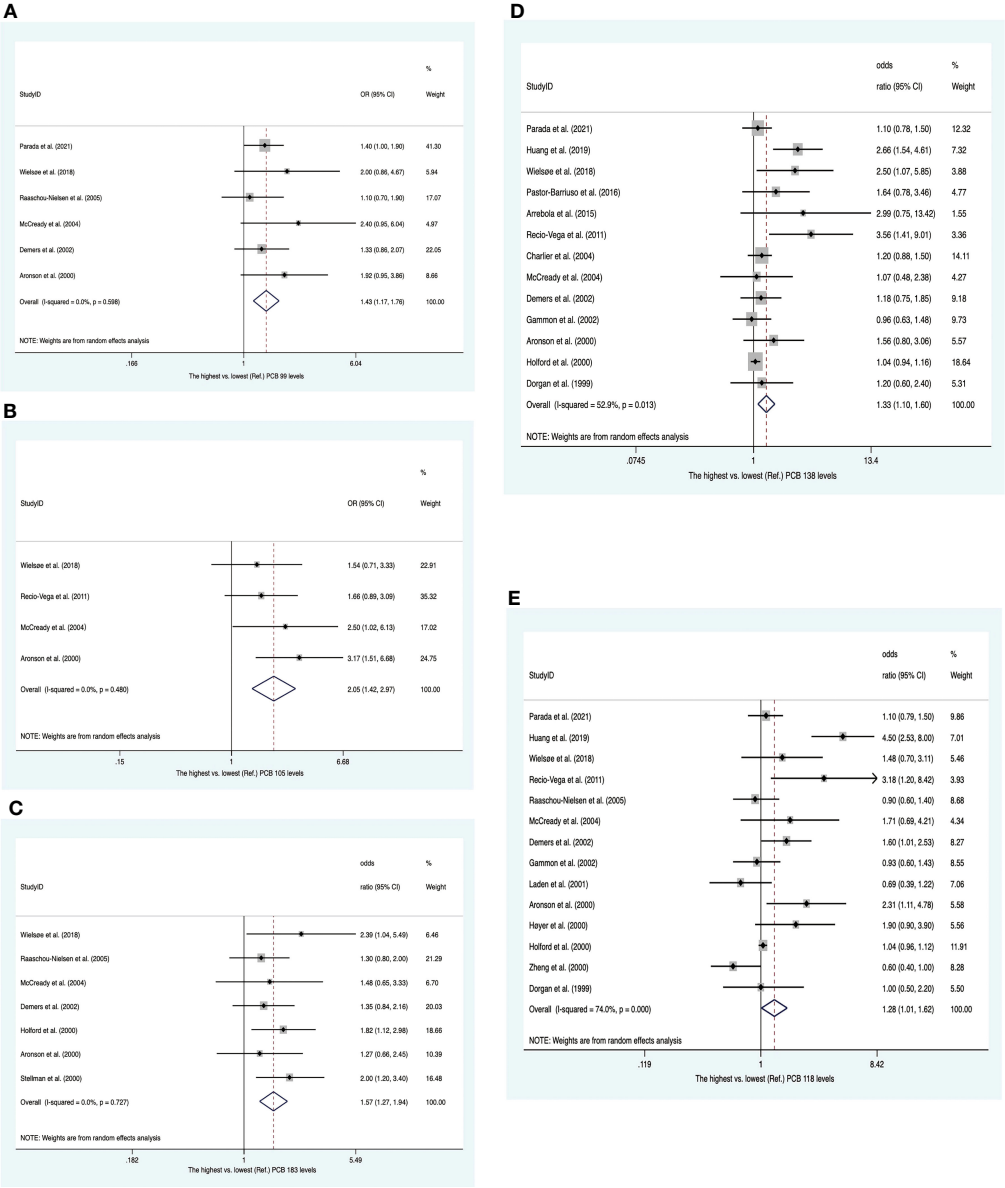
Summary estimates of the meta-analysis. **(A)** Associations between PCB 99 and breast cancer. **(B)** Associations between PCB 105 and breast cancer. **(C)** Associations between PCB 183 and breast cancer. **(D)** Associations between PCB 138 and breast cancer. **(E)** Associations between PCB 118 and breast cancer.

Twenty-two studies were enrolled, including four nested case−control studies and eighteen case−control studies (**Supplementary Table 6**). Of these, eight publications reported PCB concentrations from adipose tissues, whereas others presented concentrations of PCBs from blood samples. Twenty-two publications provided 13 summary risk estimates for breast cancer. The pooled ORs showed that individuals with higher blood/fat levels of PCB 99, PCB 105 and PCB 183 increased the risk of breast cancer (OR 1.43; 95% CI, 1.17-1.76; OR 2.05; 95% CI, 1.42-2.97; OR 1.57; 95% CI, 1.27-1.94) with no heterogeneity (**Figures 7A–C**). The summary ORs for PCB 118 and PCB 138 were statistically significantly elevated with high heterogeneity between them (OR, 1.28; 95% CI, 1.01–1.62; I^2 ^= 74.0%; OR, 1.33; 95% CI, 1.10–1.60; I^2 ^= 52.9%) (**Figures 7D, E**). These heterogeneities were affected by subgroups of sample type and study design. In subsequent subgroup analysis, we found that PCB 118 in case−control studies and PCB 138 in blood samples were positively associated with breast cancer risk (OR, 1.38; 95% CI, 1.04–1.83; I^2 ^= 77.7%; and OR, 1.29; 95% CI, 1.05–1.60; I^2 ^= 30.8%) (**Supplementary Figures 10**, **11**). The summary estimate for PCB 187 was near to unity (OR, 1.23; 95% CI, 1.00–1.53) with low heterogeneity (I^2 ^= 24.6%) (**Supplementary Figure 12**). In addition, the pooled ORs of PCB 52, PCB 74, PCB 101, PCB 153, PCB 156, PCB 170, and PCB 180 showed no significant increase in breast cancer (**Supplementary Figures 13–19**).

### Phthalates and breast cancer

**Figure 8:**
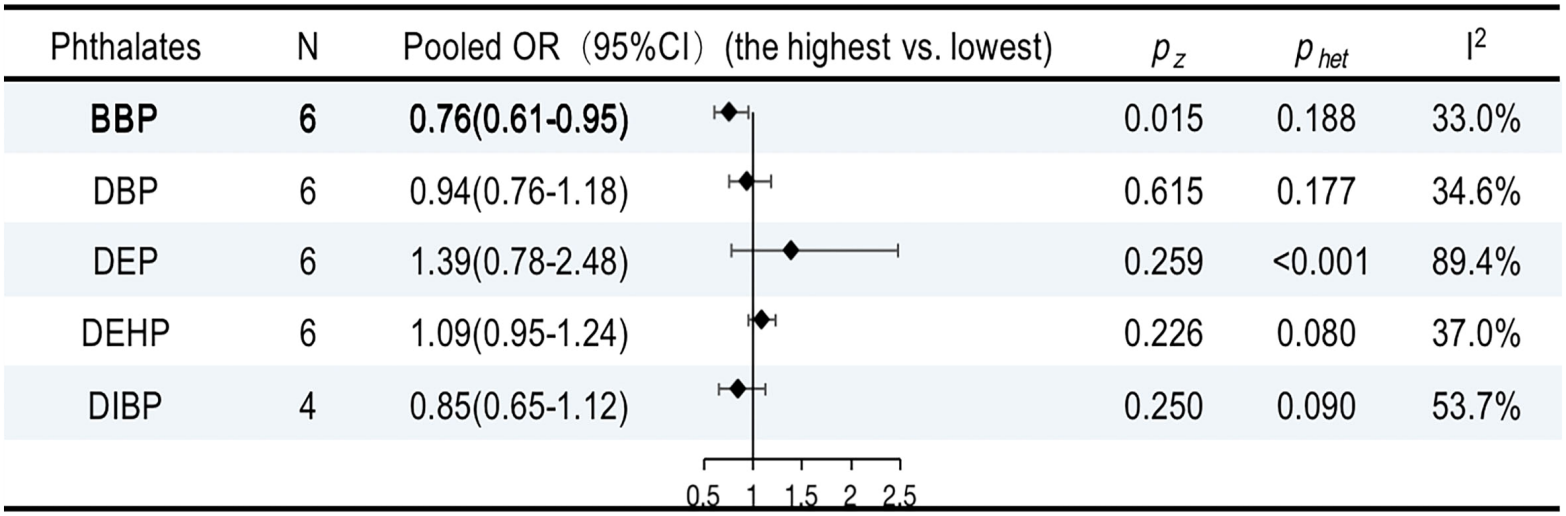
ORs (95% CI) of the summary estimate of analyses for associations between phthalates and breast cancer.

The characteristics of the studies included in our review are shown in **Supplementary Table 7**. Six publications provided five summary risk estimates for breast cancer. The urinary benzyl butyl phthalate (BBP) was negatively associated with breast cancer (OR, 0.76; 95% CI, 0.61–0.95; I^2 ^= 33.0%; *P* = 0.1888). However, the overall ORs for DBP, DEHP, DEP, and DIBP were not statistically significant (**Supplementary Figures 20–24**). The results are summarized in **Figure 8**.

### Per- and polyfluoroalkyl substances and breast cancer

**Figure 9:**
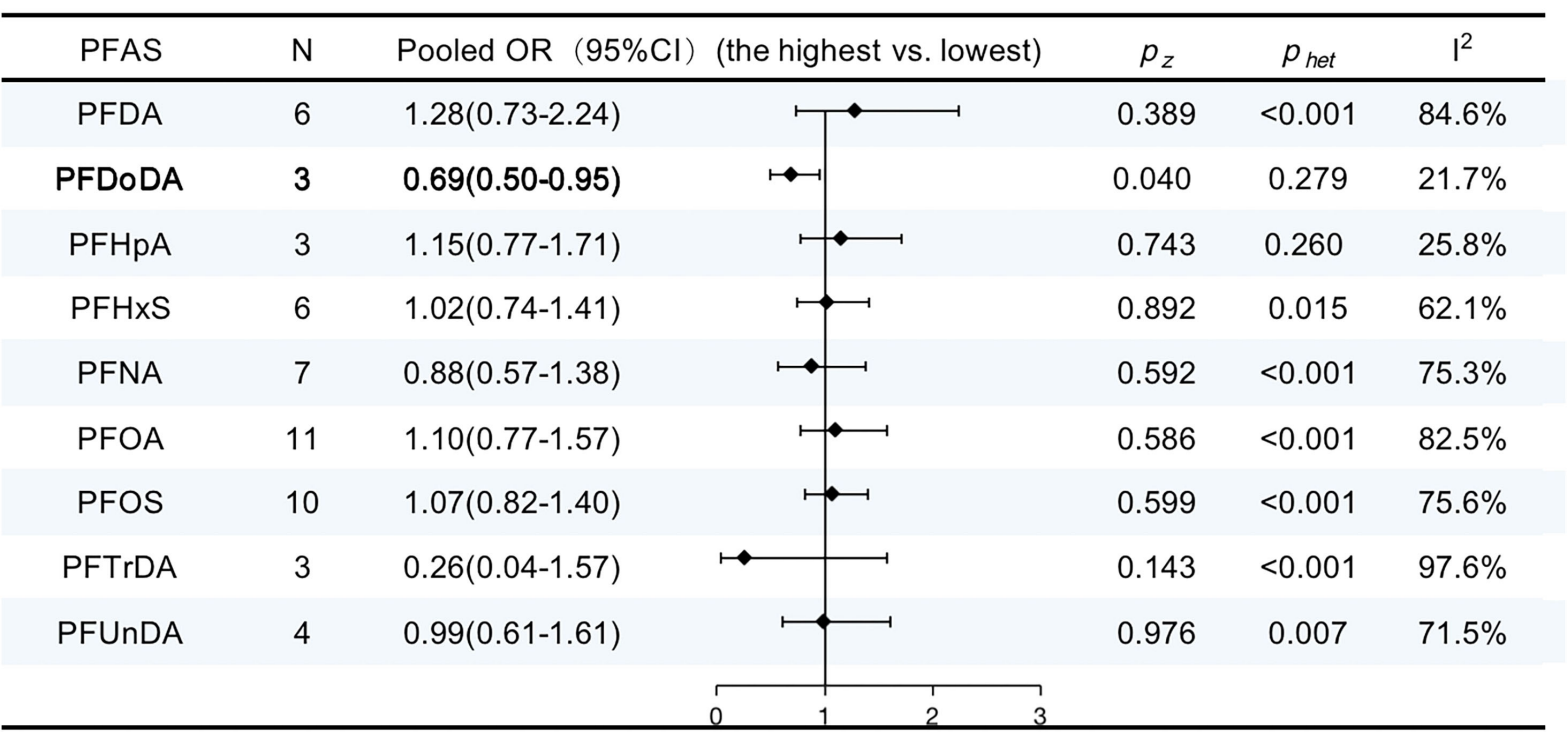
ORs (95% CI) of the summary estimate of analyses for associations between PFAS and breast cancer.

The characteristics of the studies included in our review are shown in **Supplementary Table 8**. Eleven publications provided nine summary risk estimates for breast cancer following exposure to PFASs. The summary estimates were above unity for perfluorooctanoic acid (PFOA), perfluorooctanesulfonic acid (PFOS), perfluorodecanoic acid (PFDA), perfluorohexanesulfonic acid (PFHxS), and perfluoro heptanoic acid (PFHpA) but were not statistically significantly elevated. Conversely, the pooled ORs were below unity for perfluorononanoic acid (PFNA), perfluoro undecanoic acid (PFUnDA), perfluoro-n-tridecanoic acid (PFTrDA), and perfluorododecanoic acid (PFDoDA) but only statistically significantly decreased for PFDoDA (OR, 0.69; 95% CI, 0.50–0.95; I^2 ^= 21.7%). The results are summarized in **Figure 9**.

### Polybrominated diphenyl ethers and breast cancer

**Figure 10:**
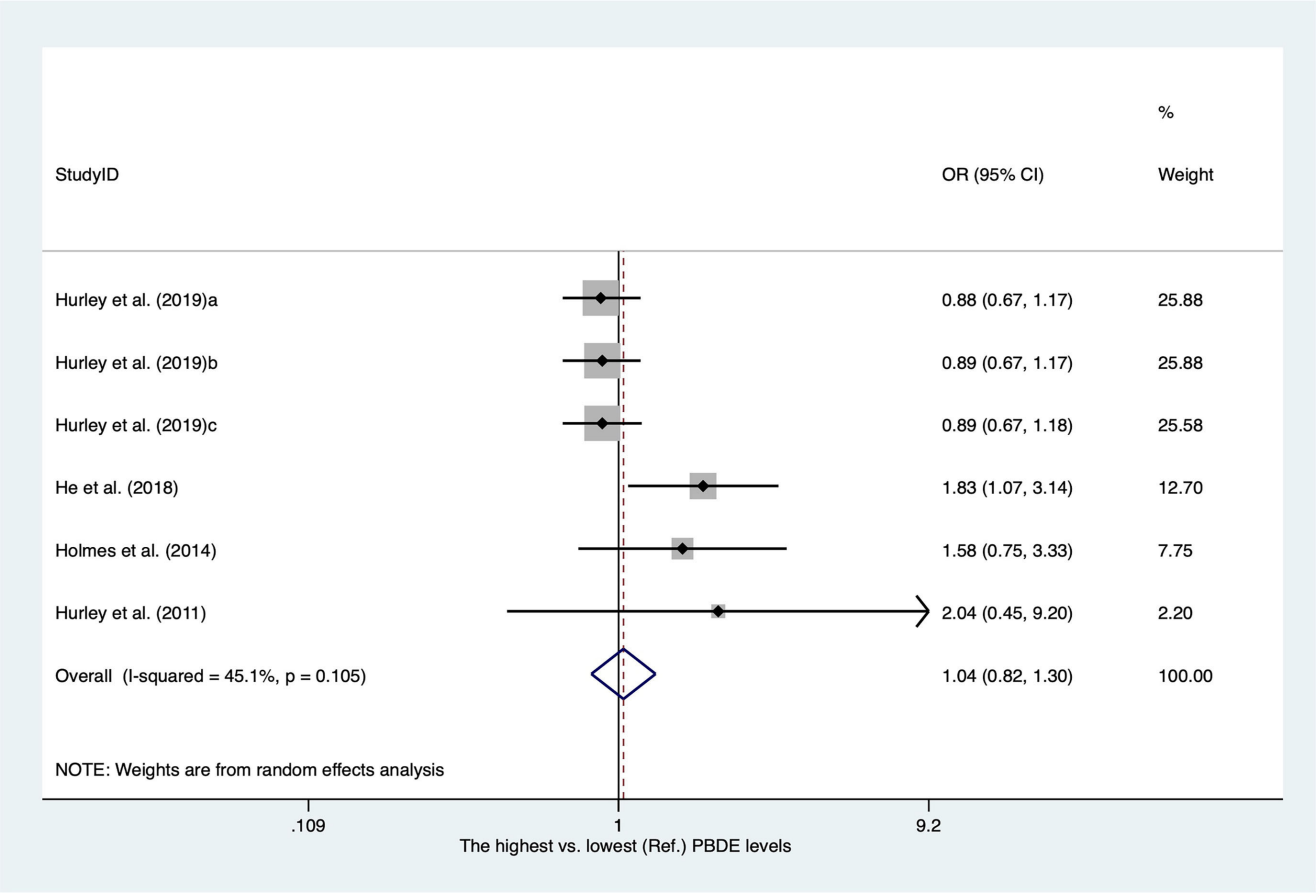
Summary estimates of the meta-analysis: association between PBDE exposure and breast cancer.

There were only four publications for polybrominated diphenyl ethers (PBDEs) included in our meta-analysis. The characteristics of the studies are shown in **Supplementary Table 9**. As shown in **Figure 10**, the overall OR for the highest versus lowest PBDE levels was 1.04 (95% CI, 0.82–1.30; I^2 ^= 45.1%).

### Bisphenol A and breast cancer

**Figure 11:**
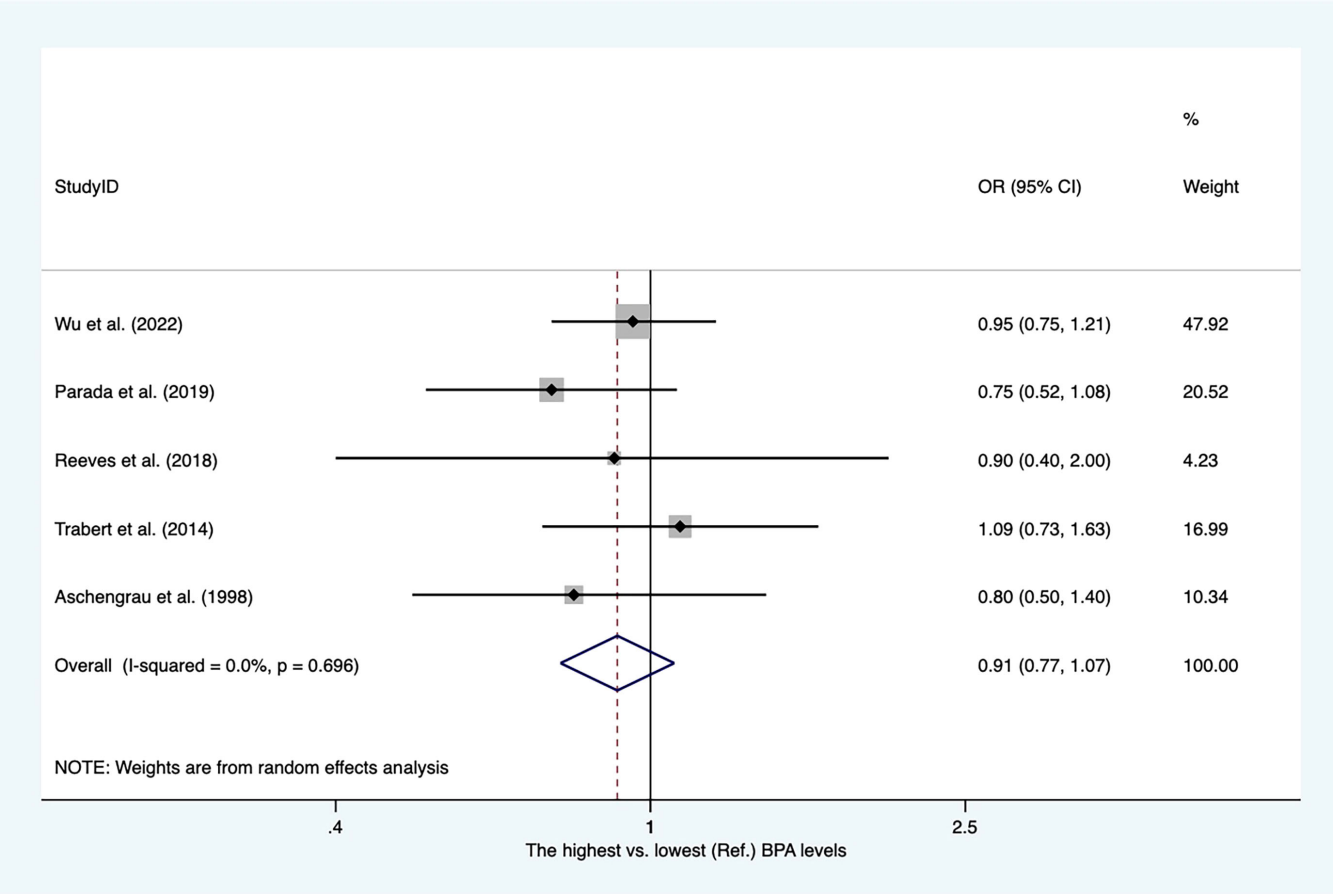
Summary estimates of the meta-analysis: association between PBA exposure and breast cancer.

The characteristics of the studies included in our meta-analysis are shown in **Supplementary Table 10**. There were four case−control studies and one nested case−control study. Four articles reported PBA levels from blood serum. As shown in **Figure 11**, the overall OR for the highest versus lowest PBA levels was 0.91 (95% CI, 0.77–1.07).

### Risk of bias assessment

**Figure 12:**
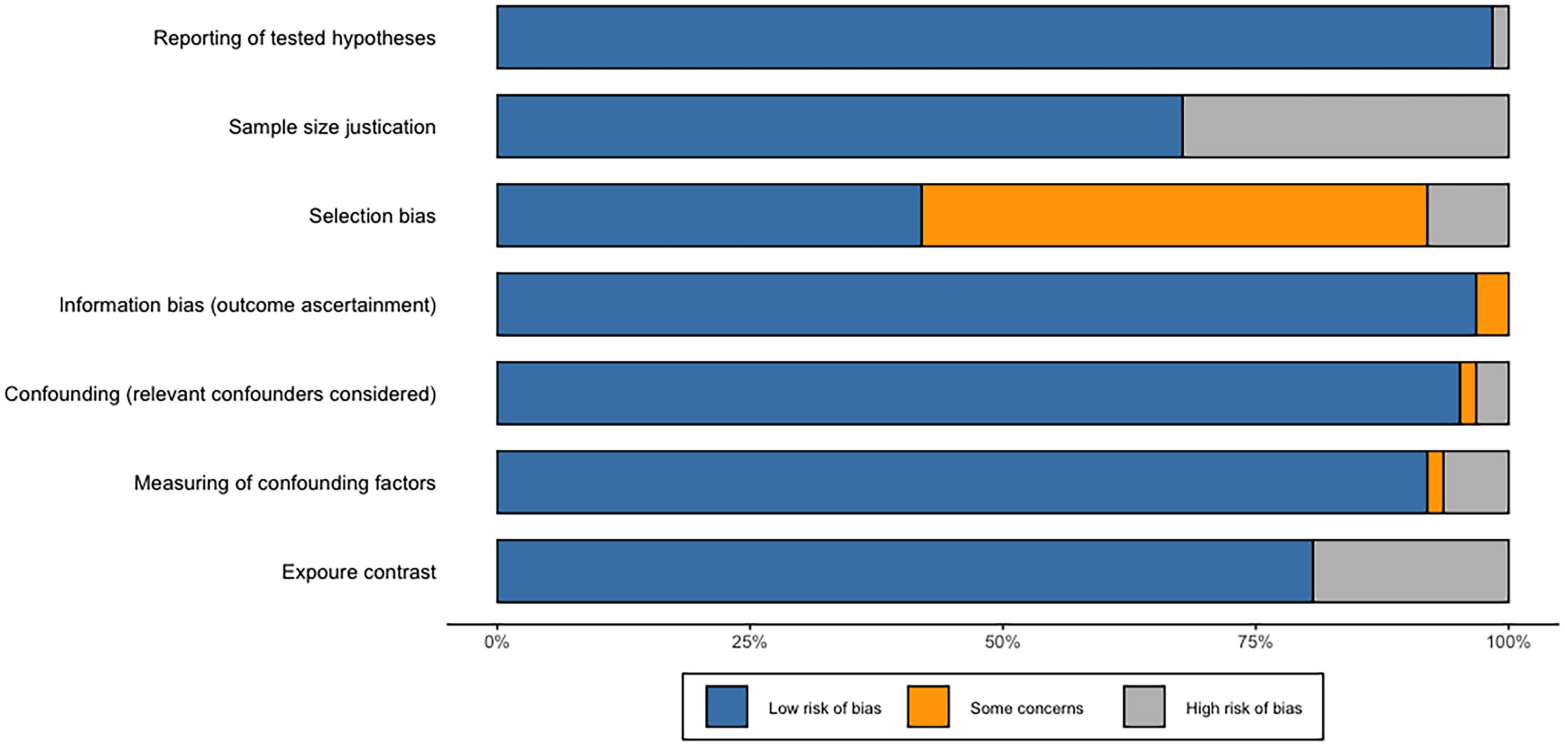
Risk of bias. The proportion of included publications with each of the identified risk categories (low risk, some concerns, and high risk).

**Figure 12** summarizes the risk of bias assessment for randomized controlled trials and crossover trials that are included. Overall, most of the included publications reported tested hypotheses, and there was a low risk of bias for information bias. More than 90% of studies considered relevant confounders and measured confounding factors. Five of all papers were considered to have a high risk of selection bias, and the bias in the other 31 studies were not clearly described. Approximately 30% of all studies did not report whether they addressed sample size in the discussion. Fourteen articles had a high risk of exposure contrast because exposure categories were split by the median or by *ad-hoc* grouping comparison of median values in cases and controls rather than divided by tertiles and quantiles (or more detailed) or by grouping of levels. For example, this case–control study that only contrasted the median values in cases and control has assessed as high risk of exposure contrast (15).

## Discussion

This meta-analysis aimed to pool available epidemiological evidence on 10 compound groups of EDCs and breast cancer. We finally pooled six compound groups because of limited publications. We included publications with real measurements of the chemical in biospecimens because reliable exposure assessment is necessary for the compounds of interest. This is a meta-analysis that, to our knowledge, has rigorously assessed the epidemiological data on the association between common endocrine-disrupting compounds and breast cancer. A total of 67 articles provided over 300 risk estimates regarding EDC exposure and breast cancer.

### Meta-analysis

#### DDT and breast cancer

Paul Müller discovered that DDT can kill insects in 1939, and it has been widely used in agriculture since then (16). The results of this meta-analysis showed that the most recent body of literature supported a moderately positive relationship between DDT/DDE and breast cancer. DDT was a common organochlorine pesticide (OCP) during the 1940s and 1950s (17). Although many countries banned DDT from agricultural usage in the 1970s, especially in developed countries, the pollution still exists in the environment and in the food chain (5). There is growing interest in DDT/DDE exposure to breast cancer that has been evaluated and recognized by many systematic reviews and meta-analyses (11, 18, 19). Differing from meta-analyses conducted in 2013, we analyzed the risk of four isoforms of DDT: p,p′-DDT, p,p′-DDE, o,p′-DDT, and p,p′-DDD, respectively. These statistical results revealed that p,p′-DDT and p,p′-DDE were marginally associated with a higher risk of breast cancer with moderate to high levels of heterogeneity although the associations were weak. However, we did not observe consistent results after stratifying by study design and sample type. Overall, DDT/DDE in blood serum, not in adipose tissue, was positively associated with breast cancer. One possible explanation was that most of the control’s adipose tissues came from those people with benign breast disease, which may confuse our final results. Unfortunately, we did not observe a positive relationship from nested case−control publications. There were only eight publications designed as prospective cohort, and these nested case−control publications most published before 2009 excluded one published in 2019. Cohn et al. found that blood serum p,p′-DDT was positively associated with breast cancer risk in 2019 (20), which was consistent with our analysis. A growing body of studies have analyzed the underlying mechanisms. Among of them, the estrogen-like properties of DDT are considered the most likely mechanism because overexpression of the estrogen hormone is associated with an increased risk for breast cancer (21, 22). Unfortunately, there were only four publications regarding o,p′-DDT and p,p′-DDD, respectively. Thus, the impact of o,p′-DDT and p,p′-DDD exposure on breast cancer cannot be determined in our review. More prospective studies are needed to clarify the relationship between DDT/DDE and breast cancer.

#### Other pesticides and breast cancer

In addition, we pooled another nine OCPs. Thereinto, HCH and chlordane were also related to breast cancer risk. However, in subgroups stratified by sample type, the summary OR for HCH in adipose tissue was significantly reduced. There were only three publications addressing HCH in adipose tissues, and these articles were published before 2005. Meanwhile, no association was observed for HCH from nested case−control studies and breast cancer. There were only four nested case−control publications, and these studies were conducted before 2008. To our knowledge, this is the first meta-analysis to analyze the relationship between HCH and breast cancer.

#### PCBs and breast cancer

The International Agency for Research on Cancer (IARC) upgraded PCBs to group 1 “Carcinogenic to humans” in 2015, on the basis of sufficient evidence of an excess risk for melanoma (23). In recent decades, an increasing number of epidemiological studies have investigated the connection between PCBs and the risk of breast cancer. However, the results were inconsistent. Two meta-analyses were conducted to investigate the relationship between individual PCB congeners and breast cancer in 2015 and 2016 (24, 25). As epidemiological evidence has been updated in recent years (26–31), we further evaluated the association between PCBs and breast cancer. In 1995, Wolff and Toniolo classified PCB congeners into three groups: (i) group 1, containing PCBs that act as estrogen agonists, such as PCB 187; (ii) group 2, containing PCBs that act as dioxin, such as PCB 105, PCB 118, PCB 138, PCB 156, and PCB 170; and (iii) group 3, containing PCBs that work by stimulating cytochrome P450 enzymes, such as PCB 99, PCB 153, PCB 180, and PCB 183 (32). Zhang et al. found that group 2 and group 3 PCB exposure, but not group 1 PCB exposure, increased the risk of breast cancer in 2015 (25). However, it proved challenging to identify which specific PCB congeners are associated with breast cancer. In our review, 13 PCB congeners were reported by more than two studies. Similarly, we found that the highest (vs. lowest) tertiles of PCB 99 and PCB 183 (group 3) were positively associated with the risk of breast cancer in our analysis, which was consistent with the meta-analysis conducted in 2016. In addition, we also found that the risk of breast cancer can be increased by PCB 105, PCB 118, and PCB 138 (group 2).

#### Other EDCs and breast cancer

There are numerous unavoidable and accidental causes of exposure to BPA, phthalates, PBDEs, and PFASs in daily life. We found that one PFAS congener (PGDoDA) and one phthalate congener (BBP) were passively linked with the risk of breast cancer. However, only three publications addressing PGDoDA matched the requirements for meta-analysis. More studies are needed to identify the association. The impact of other phthalates, PFAS, BPA, and PBDE on breast cancer was not sufficiently supported by the results.

#### Studies not eligible for meta-analysis

In addition, there were four compound groups of EDCs that were not eligible for meta-analysis because of limited studies. Further larger population-based studies are needed to clarify the real relationship between environmental EDCs and breast cancer.

There were only two studies addressing the relationship between parabens and breast cancer. Wu et al. found that breast cancer was inversely associated with total parabens (OR, 0.77; 95% CI, 0.62–0.97) in a nested case−control study in 2021 (33). Parada et al. found that there was no association between the risk of breast cancer and the highest (vs. lowest) quintiles of urine propylparaben (OR, 1.31; 95% CI, 0.90–1.90) and total parabens (OR, 1.35; 95% CI, 0.93–1.97), but the positive association was found between methylparaben and breast cancer (OR, 1.50; 95% CI, 1.03–1.18) (34). 2,3,7,8-Tetrachlorodibenzo-p-dioxins (TCDD), the most toxic congener of dioxin, is a widespread environmental contaminant that has been classified as carcinogenic to humans by the IARC (35). Two studies, conducted in 2002 and 2011, found that the TCDD levels in serum were not associated with the risk of breast cancer (36, 37). However, Rhee et al. found that residential exposure to dioxin emissions may confer an increased risk of breast cancer (38). Larger longitudinal studies are necessary to clarify the relationship between TCDD and breast cancer. Triclosan is a nonpersistent EDC that has caused serious public health concerns because it is widely absorbed through the skin, inhaled, and ingested. To date, only two studies have addressed the effect of individual exposure to triclosan on breast cancer. These two publications suggested that exposure to triclosan was not associated with breast cancer (33, 34). Two nested case−control studies found inconsistent results for PHA and breast cancer. In 2017, Shen et al. found that plasma PHA was positively associated with breast cancer risk (39). However, Wu et al. found no significant association between PHA and breast cancer in 2021 (40).

## Strengths and limitations of the review

We have rigorously assessed the epidemiological data on 10 compound groups of EDCs (BPA, dioxins, parabens, phthalates diesters and their metabolites, flame retardants, PAHs, PCBs, organochloride pesticides, PFAS, and triclosan) and breast cancer and, finally, summarized risk estimates for six compounds (organochloride pesticides, PCBs, phthalates diesters and their metabolites, PFAS, flame retardants, and BPA). To our knowledge, we have for the first time summarized the relationship between HCH and breast cancer, and found that HCH was positively related to breast cancer risk. Meanwhile, the relationships between several common EDC congeners and breast cancer have been updated. such as DDT, PCBs, and phthalates. This facilitated a better understanding of the association between each type of EDCs and breast cancer. Unfortunately, heterogeneity was not well explained in our review, and a limited number of available prospective studies investigating the associations between EDC exposure and breast cancer were included in our meta-analysis. More attention was given to women, not men, perhaps because breast cancer is more common in women. To elucidate the overall associations, future large, longitudinal epidemiological investigations are needed.

## Conclusions

In this meta-analysis, statistically significant associations revealed that (i) p,p′-DDT and its major metabolite p,p′-DDE were somewhat related to a greater risk of breast cancer. However, this relationship only existed in blood serum but not in adipose tissue. (ii) Breast cancer risk was increased by exposure to chlordane and HCH. (iii) Five polychlorinated biphenyls (PCB 99, PCB 105, PCB 118, PCB 138, and PCB 183) can increase the risk of breast cancer. (iv) One phthalate congener (BBP) and one PFAS congener (PFDoDA) were negatively associated with breast cancer risk. Our meta-analysis suggested that exposure to a few specific EDCs was identified as a risk factor for breast cancer. More effective preventive measures should be taken to control the environmental pollution of EDCs.

## Data availability statement

The original contributions presented in the study are included in the article/**Supplementary Material**. Further inquiries can be directed to the corresponding author.

## Author contributions

HL: Software, Data curation, Formal analysis, Writing – original draft. YKS: Data curation, Formal analysis, Writing – review & editing. LR: Data curation, Formal analysis, Writing – review & editing. JL: Data curation, Formal analysis, Writing – review & editing. YFS: Data curation, Formal analysis, Writing – review & editing. CM: Data curation, Formal analysis, Writing – original draft. CH: Conceptualization, Data curation, Project administration, Writing – original draft.

## Glossary

**Table:**

BBP	Benzyl butyl phthalate
BPA	Bisphenol A
EDCs	Endocrine-disrupting chemicals
DBP	Dibutyl phthalate
DDT	1,1-(2,2,2-trichloroethane-1,1-diyl)bis (4-chlorobenzene)
DEP	Diethyl phthalate
DEHP	Di(2-ethylhexyl) phthalate
DIBP	Diisobutyl phthalate
HCB	Hexachlorobenzene
HCH	Hexachlorocyclohexane
HR	Hazard ratio
IARC	International Agency for Research on Cancer
OCPs	Organochlorine pesticide
o,p'-DDT	1-methoxy-2-[2,2,2-trichloro-1-(4 methoxyphenyl)ethyl]benzene)
OR	Odds ratio
PAHs	Polyaromatic hydrocarbons
PBDE	Polybrominated diphenyl ethers
PCB	Polychlorinated biphenyls
PFAS	Per- and polyfluoroalkyl substances
PFDA	Perfluorodecanoic acid
PFDoDA	Perfluorododecanoic acid
PFHpA	Perfluoro heptanoic acid
PFHxS	Perfluorohexanesulfonic acid
PFNA	Perfluorononanoic acid
PFOA	Perfluorooctanoic acid
PFOS	Perfluorooctanesulfonic acid
PFTrDA	Perfluoro-n-tridecanoic acid
PFUnDA	Perfluoro undecanoic acid
p,p′-DDE	2,2-bis(4-chlorophenyl)-1,1-dichloroethylene
p,p′-DDT	1-chloro-4-[2,2,2-trichloro-1-(4-chlorophenyl)ethyl]benzene
p,p′-DDD	2,2-bis(4-chlorophenyl)-1,1-dichloroethane
RR	Relative risk
TCDD	2,3,7,8-tetrachlorodibenzo-p-dioxins
95% CI	95% confidence interval
