# Supplementary figures and images for: Endocrine-disrupting chemicals and breast cancer: a meta-analysis
Source: Front Oncol. 2023 Nov 9;13:1282651. doi: 10.3389/fonc.2023.1282651 (PMC10665889; doi:10.3389/fonc.2023.1282651)

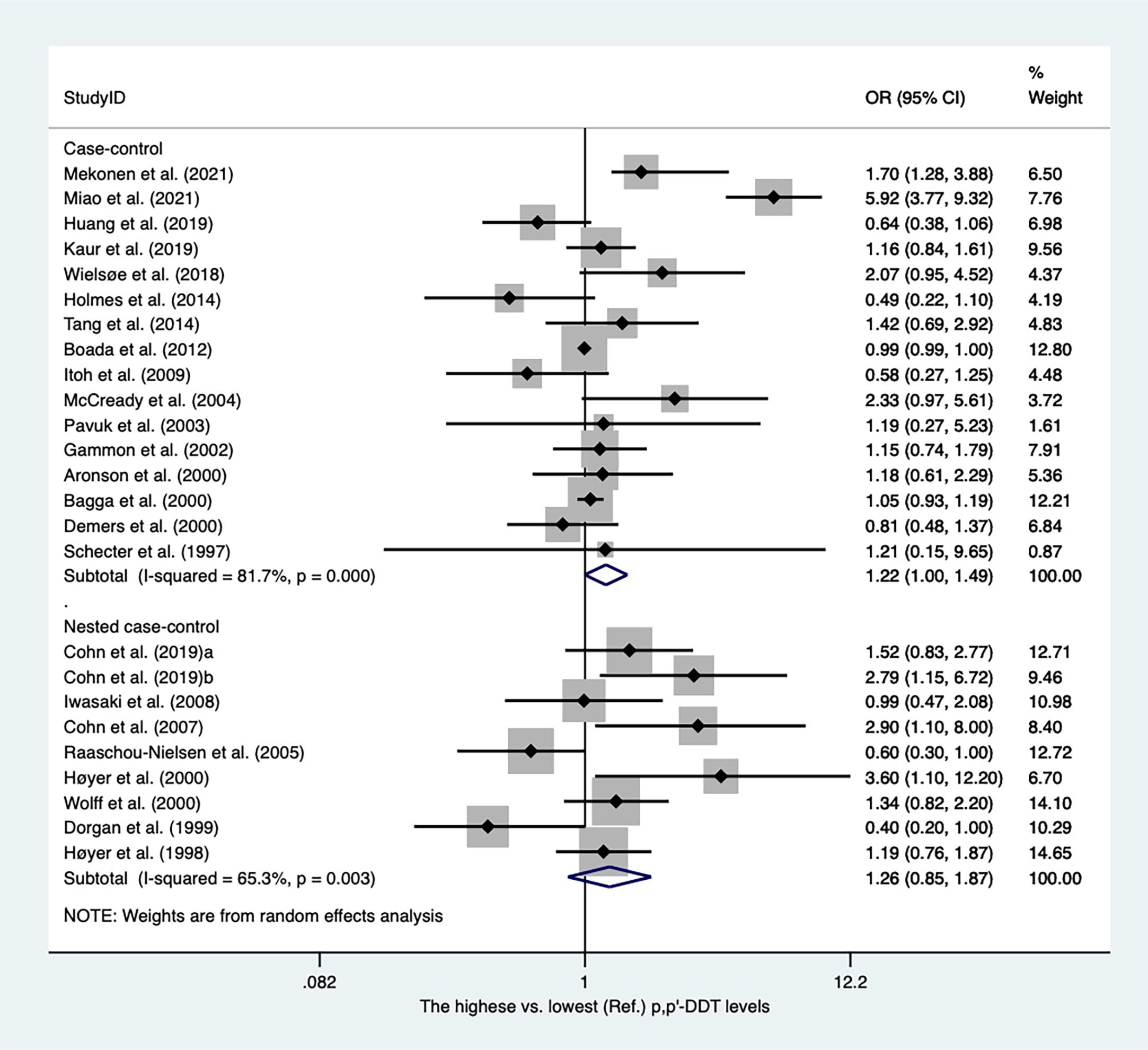

Supplement: Supplementary file 1 [file DataSheet_1.zip › Supplemetary Figures 1-24/Supplementary Figure 1.jpg]

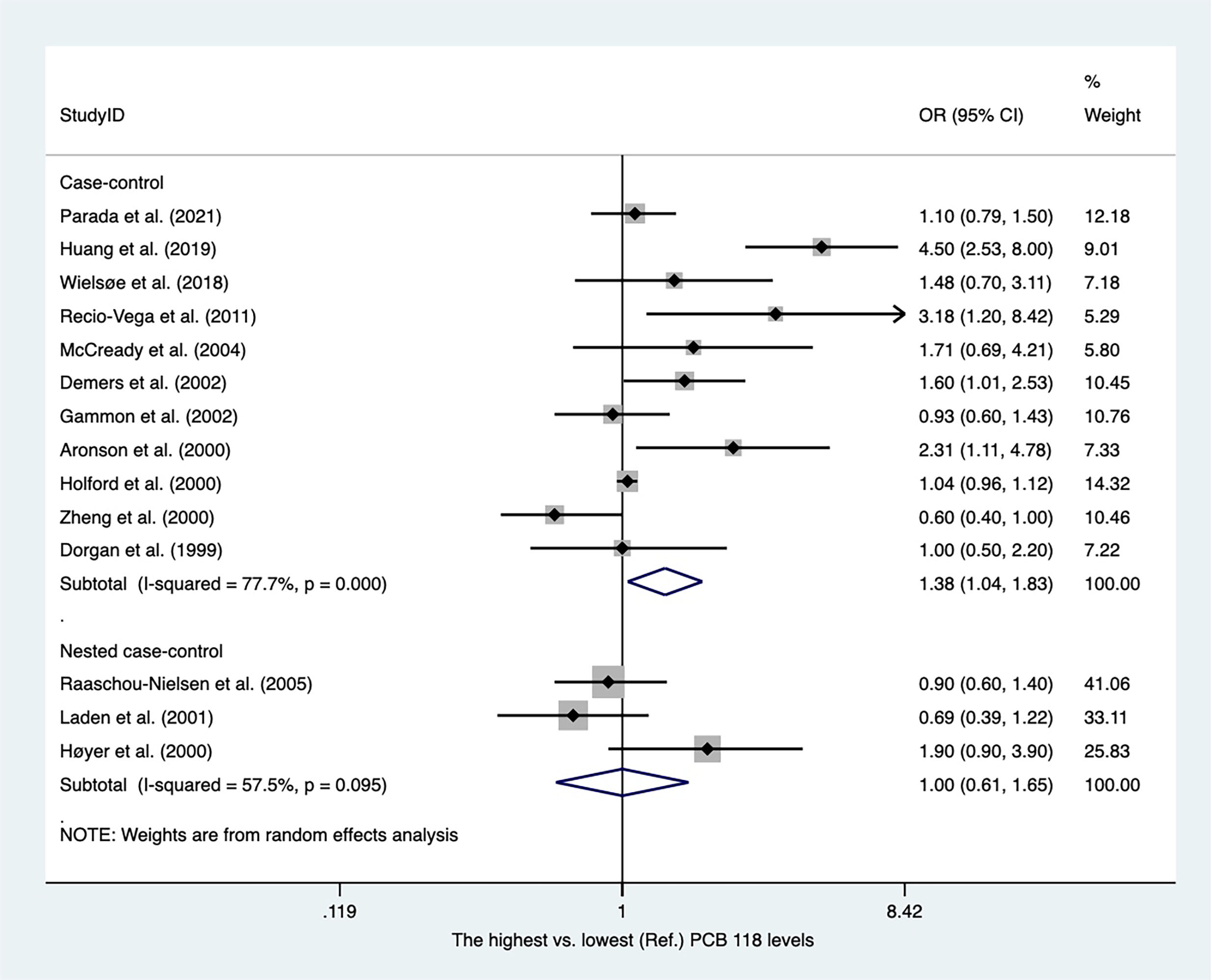

Supplement: Supplementary file 1 [file DataSheet_1.zip › Supplemetary Figures 1-24/Supplementary Figure 10.jpg]

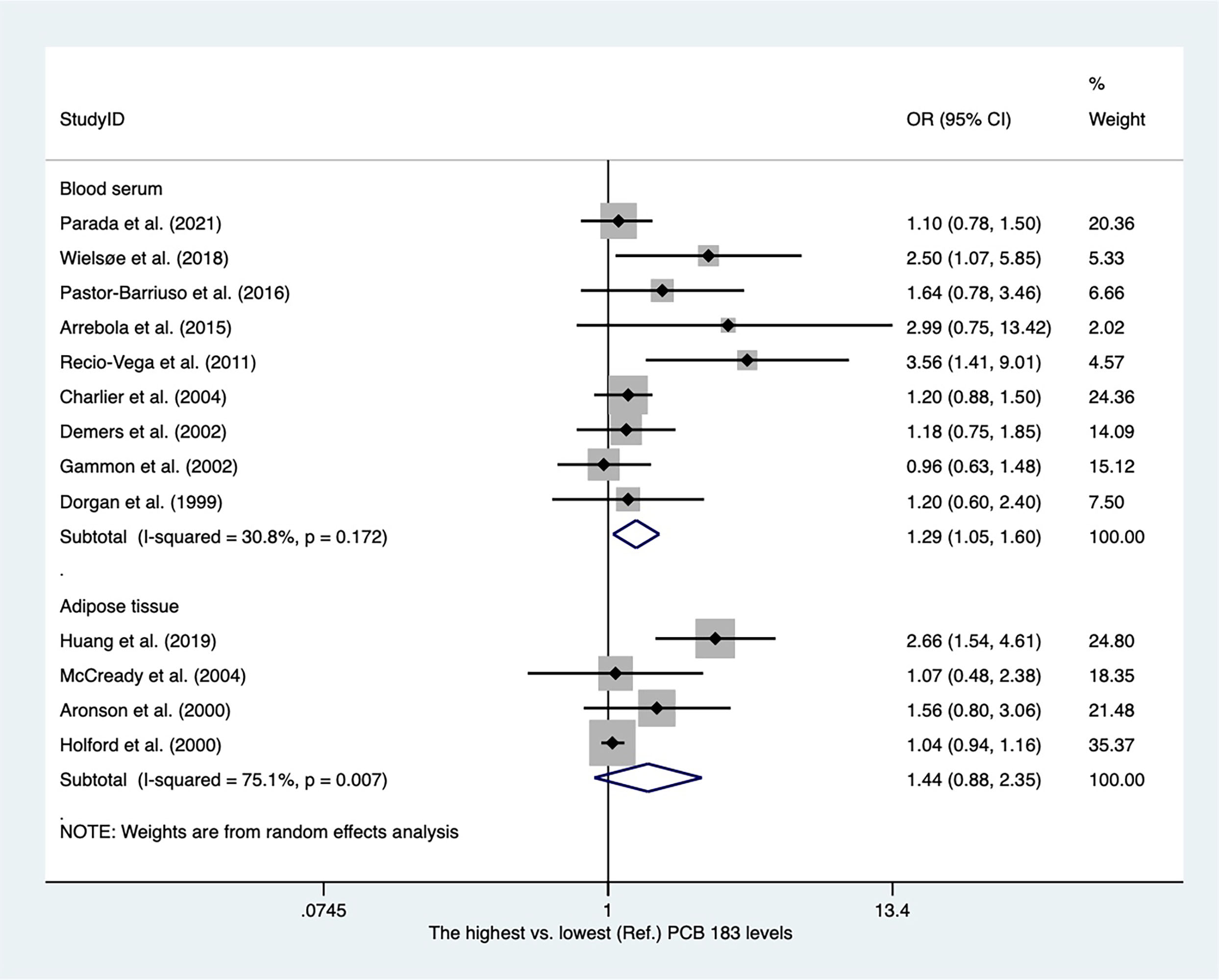

Supplement: Supplementary file 1 [file DataSheet_1.zip › Supplemetary Figures 1-24/Supplementary Figure 11.jpg]

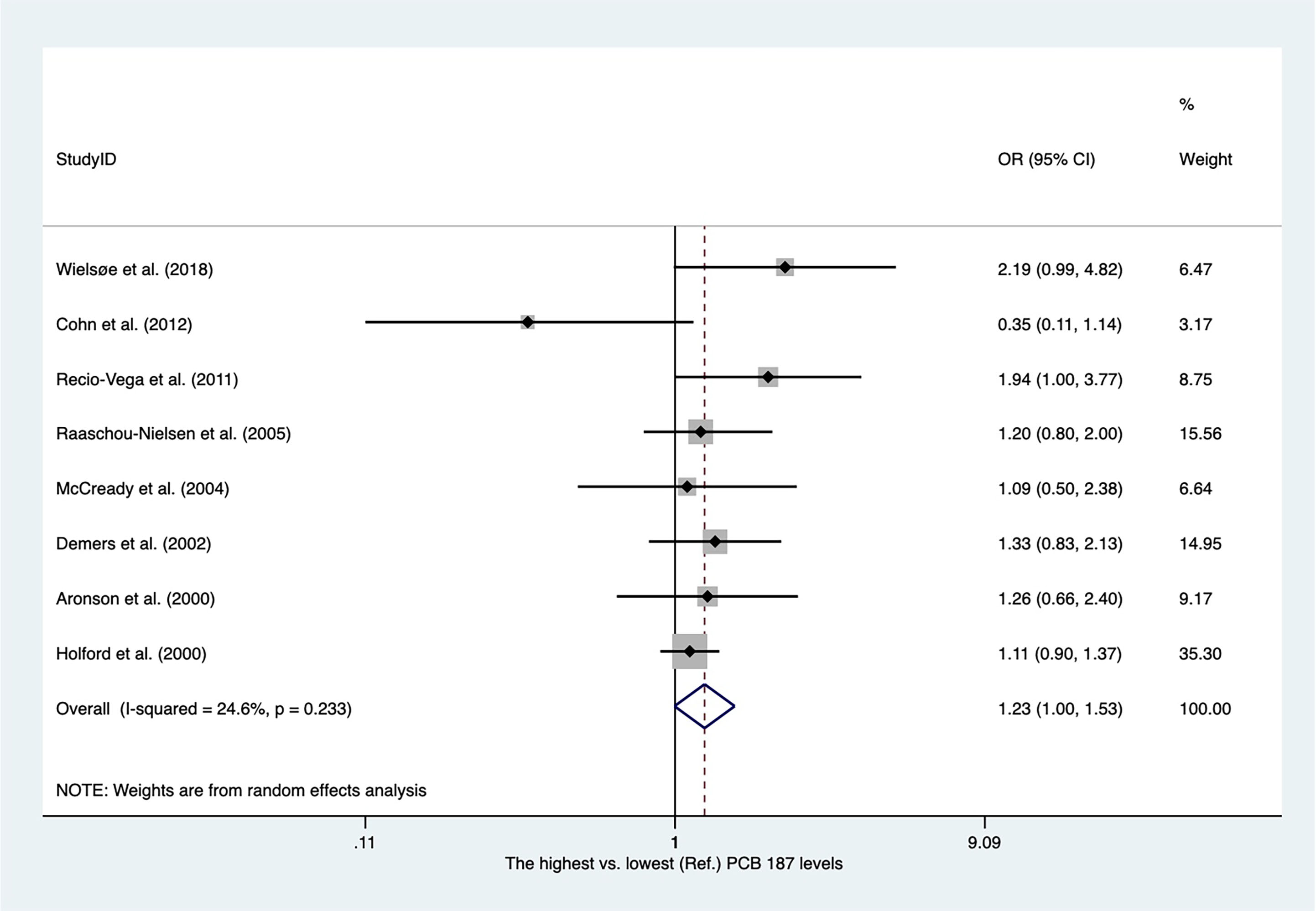

Supplement: Supplementary file 1 [file DataSheet_1.zip › Supplemetary Figures 1-24/Supplementary Figure 12.jpg]

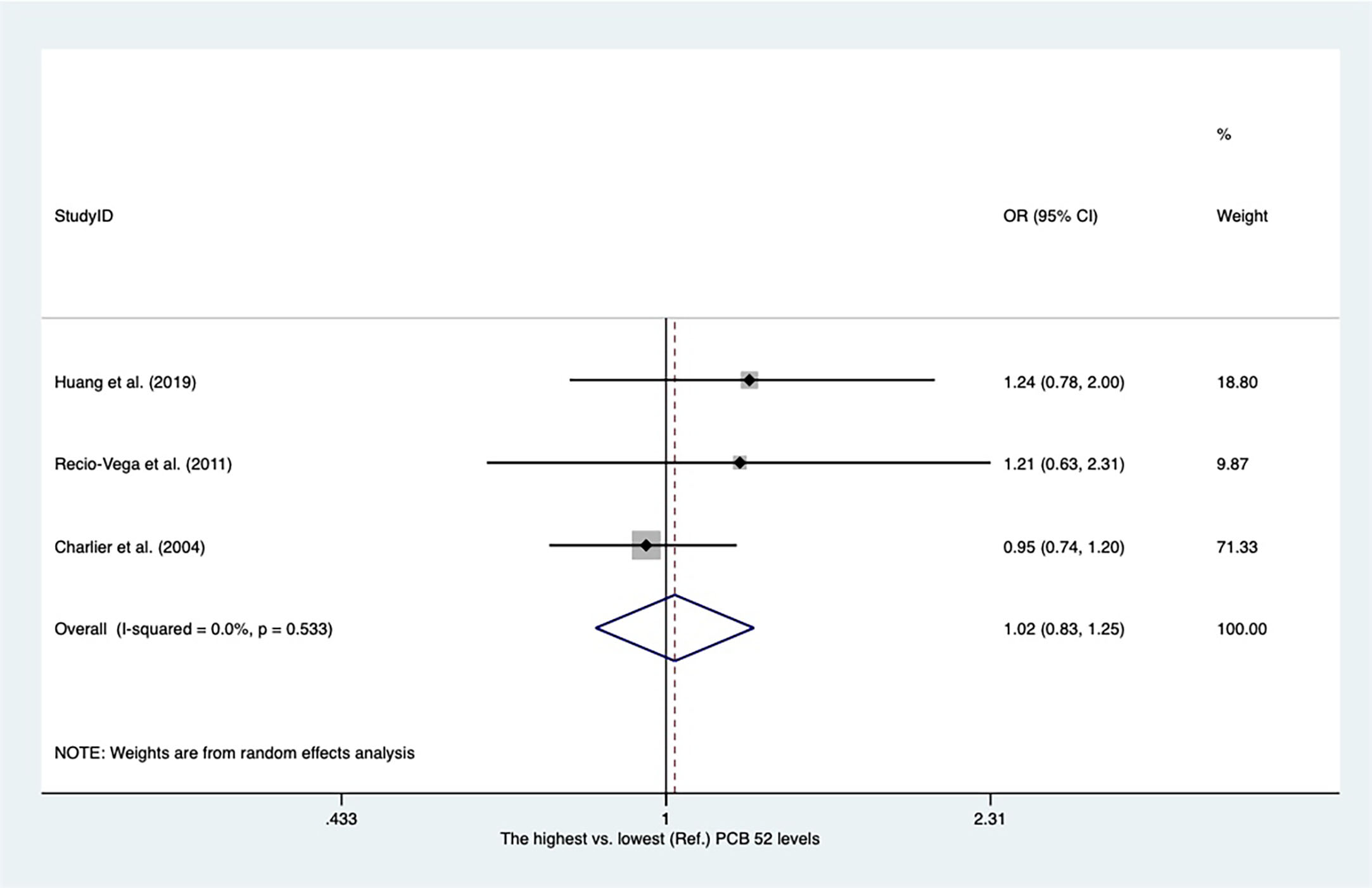

Supplement: Supplementary file 1 [file DataSheet_1.zip › Supplemetary Figures 1-24/Supplementary Figure 13.jpg]

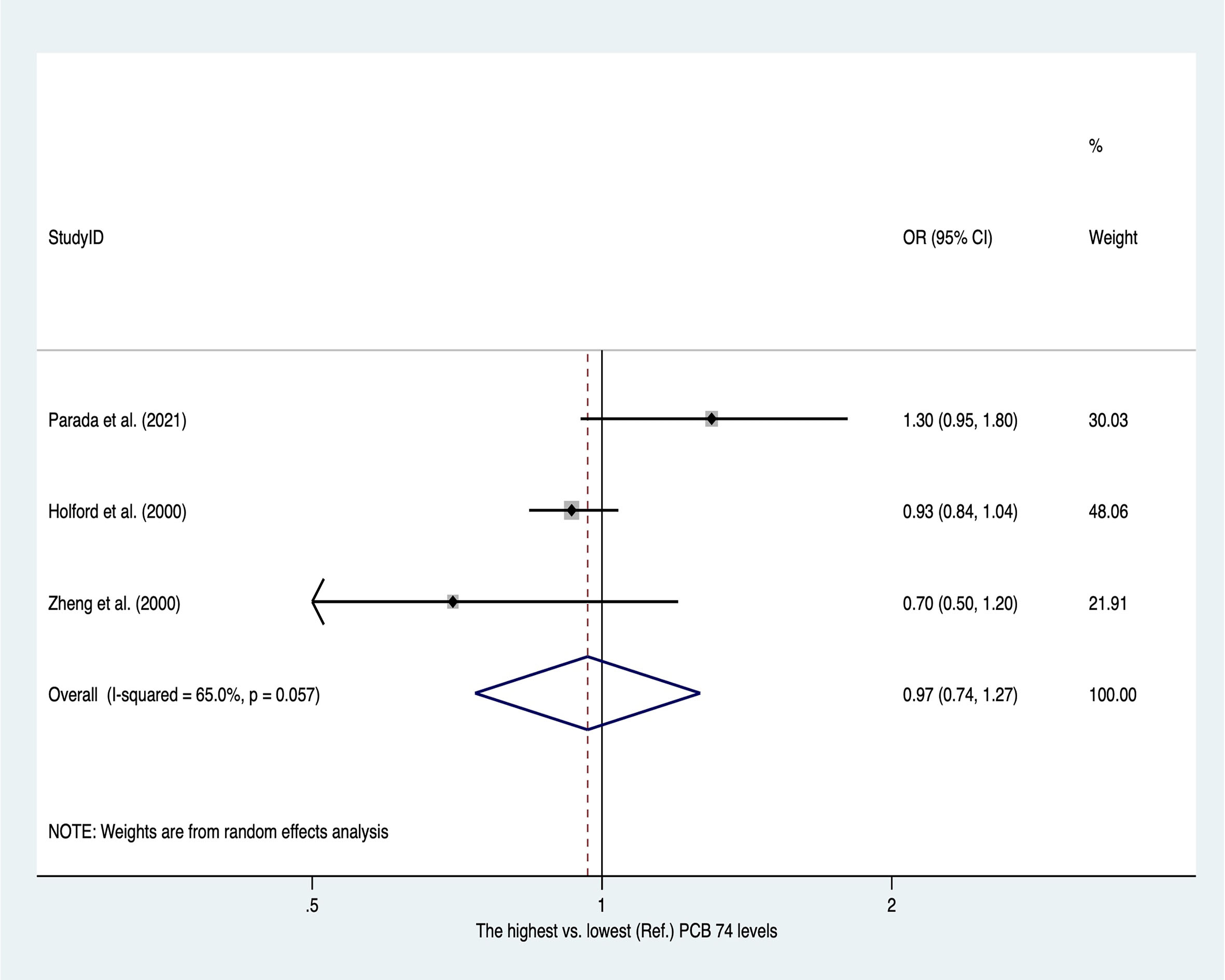

Supplement: Supplementary file 1 [file DataSheet_1.zip › Supplemetary Figures 1-24/Supplementary figure 14.jpg]

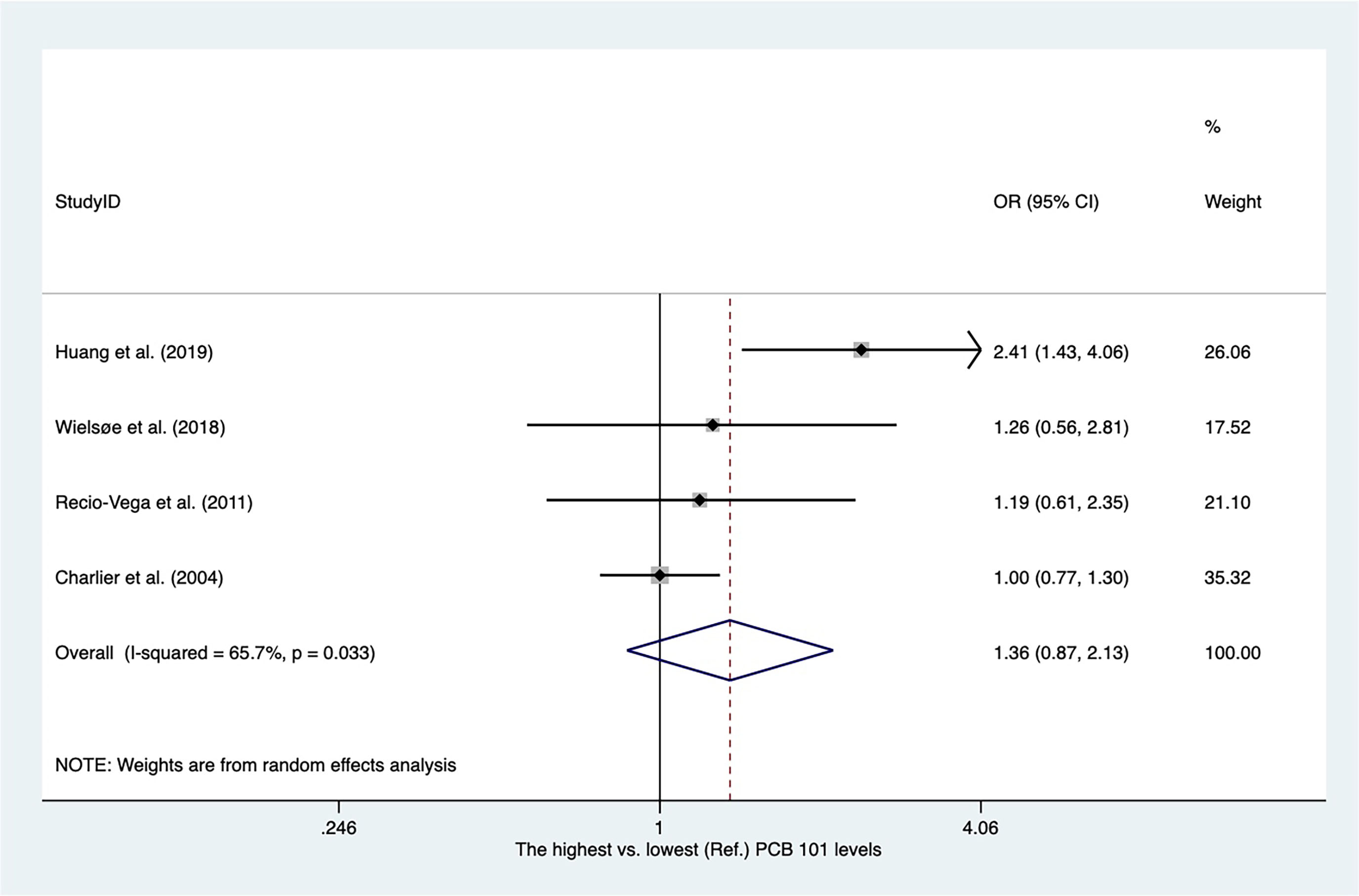

Supplement: Supplementary file 1 [file DataSheet_1.zip › Supplemetary Figures 1-24/Supplementary Figure 15.jpg]

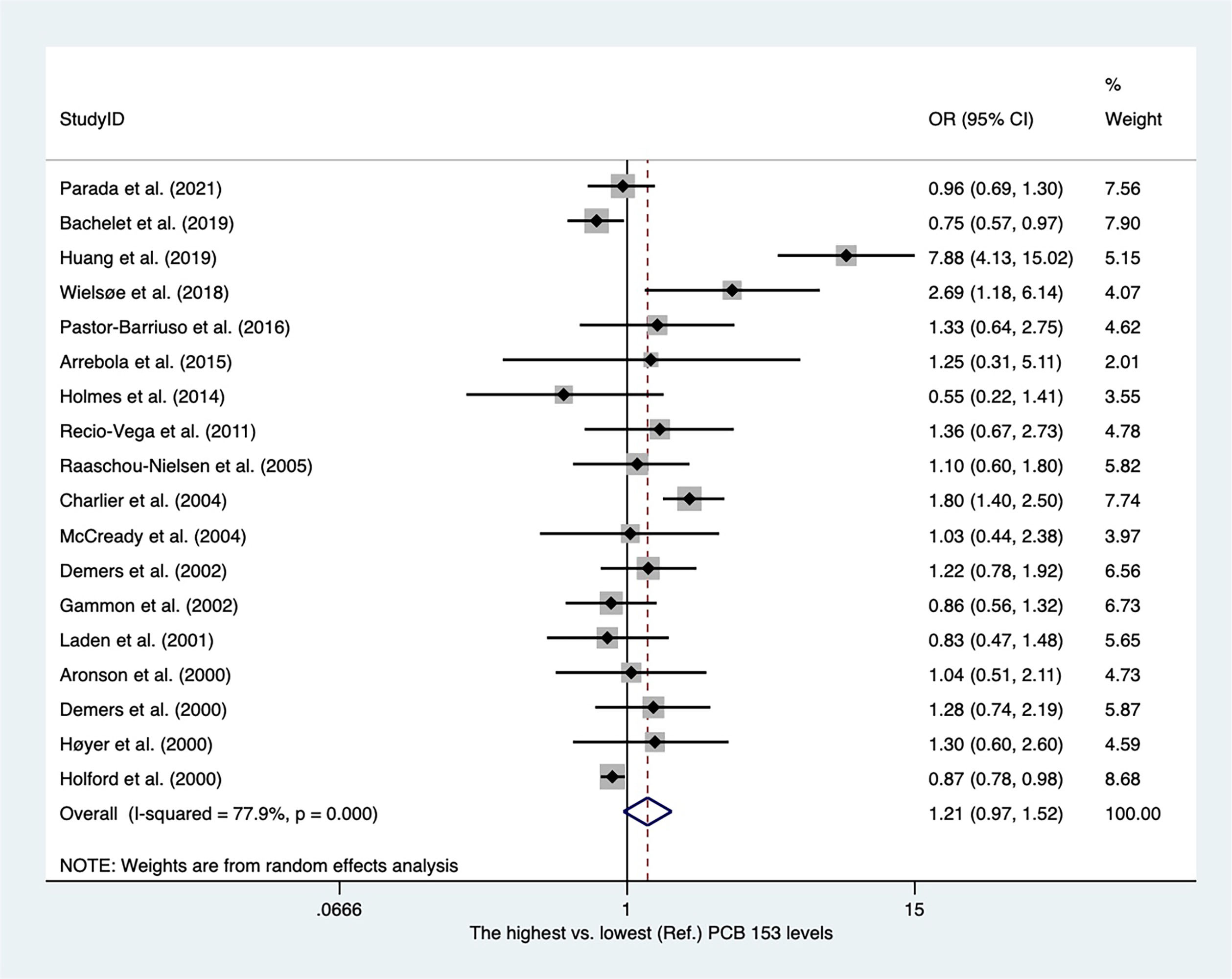

Supplement: Supplementary file 1 [file DataSheet_1.zip › Supplemetary Figures 1-24/Supplementary Figure 16.jpg]

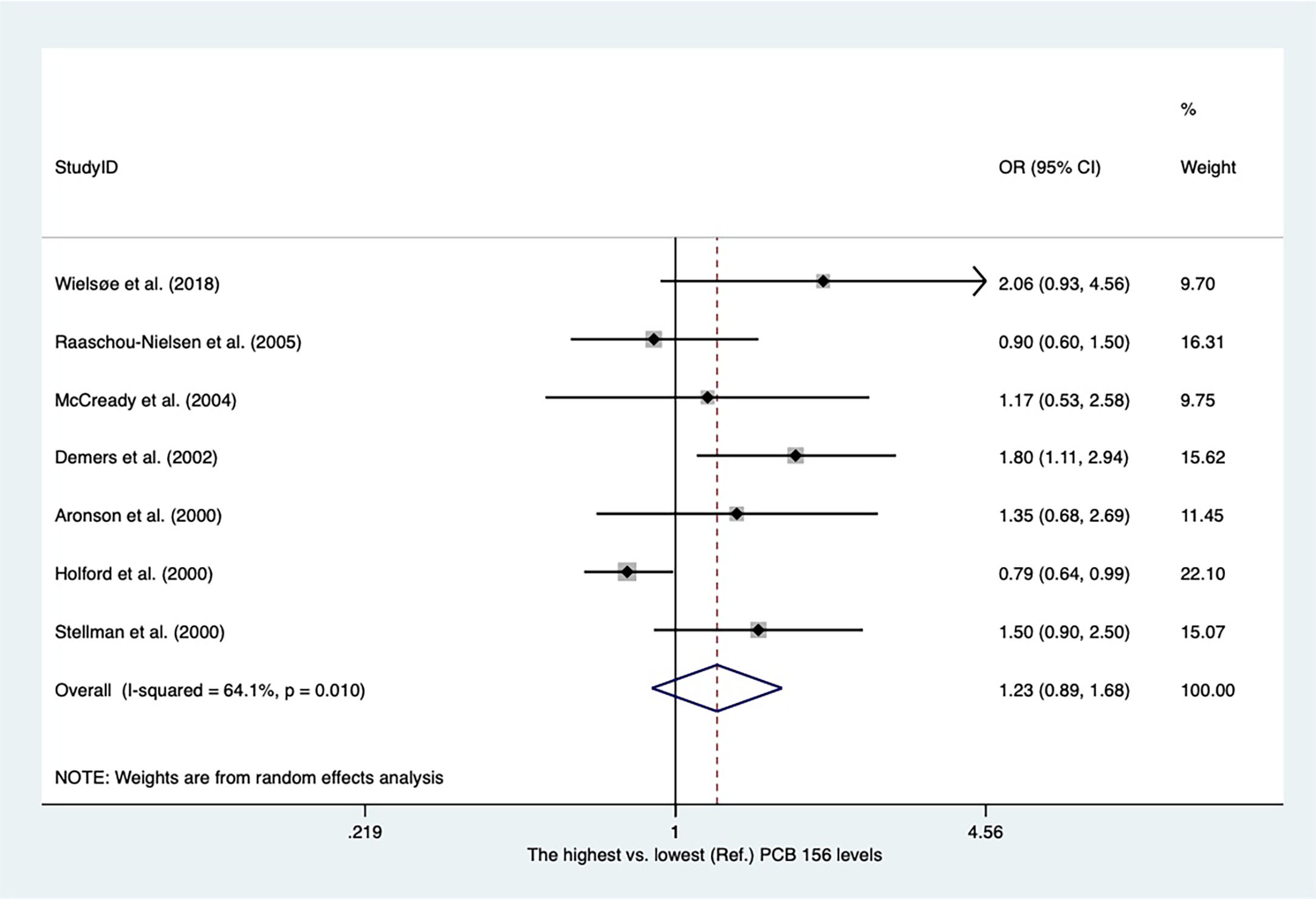

Supplement: Supplementary file 1 [file DataSheet_1.zip › Supplemetary Figures 1-24/Supplementary Figure 17.jpg]

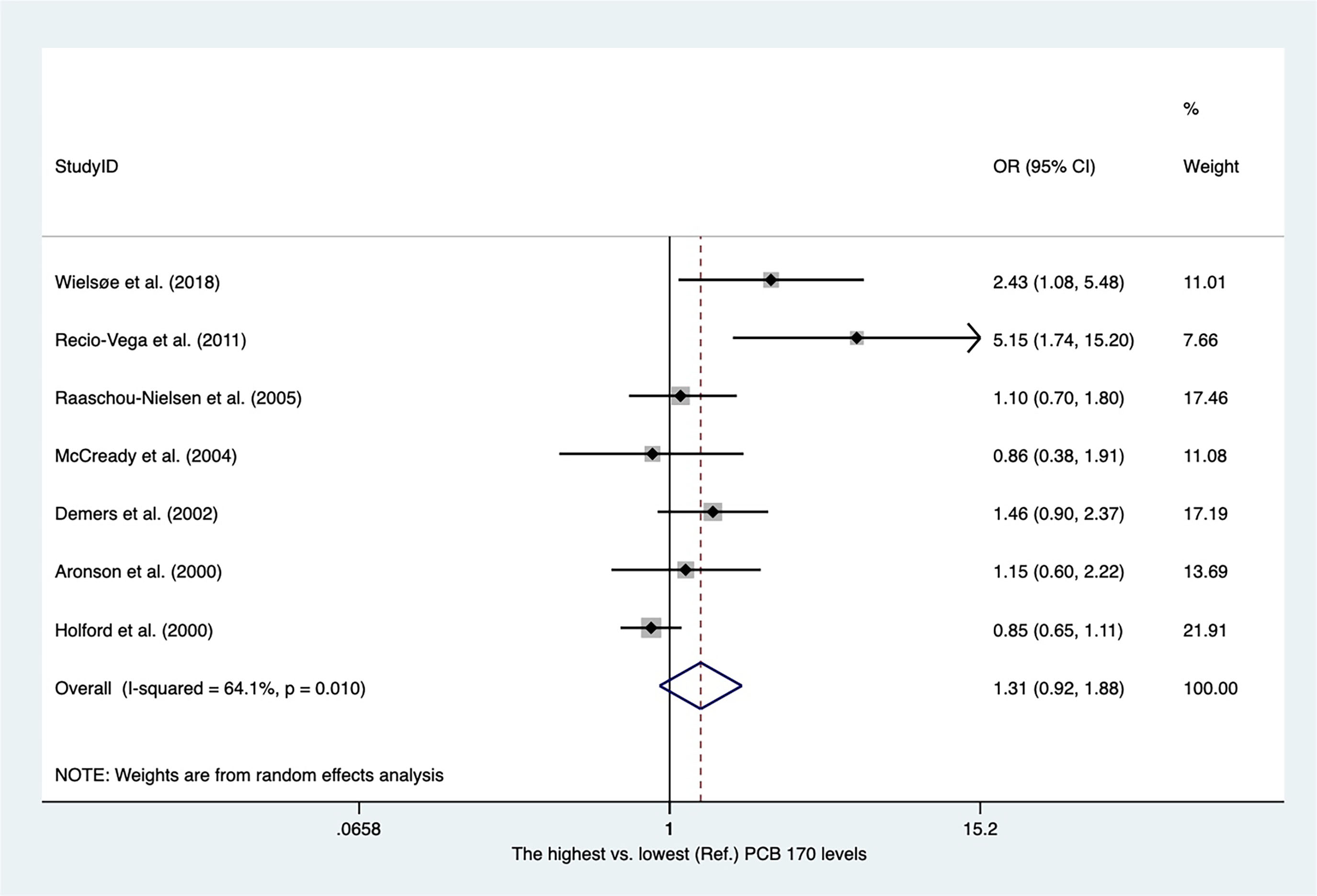

Supplement: Supplementary file 1 [file DataSheet_1.zip › Supplemetary Figures 1-24/Supplementary Figure 18.jpg]

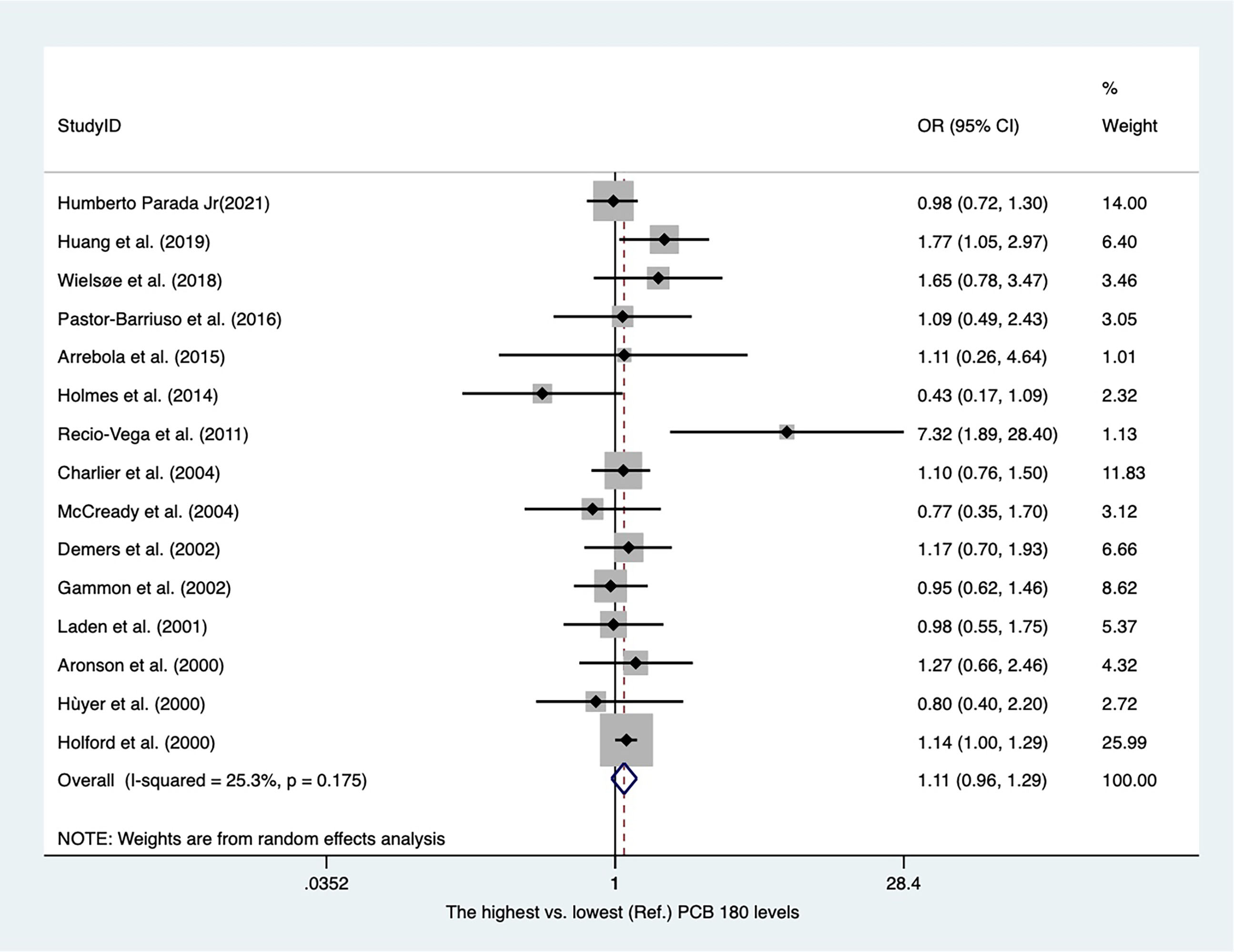

Supplement: Supplementary file 1 [file DataSheet_1.zip › Supplemetary Figures 1-24/Supplementary Figure 19.jpg]

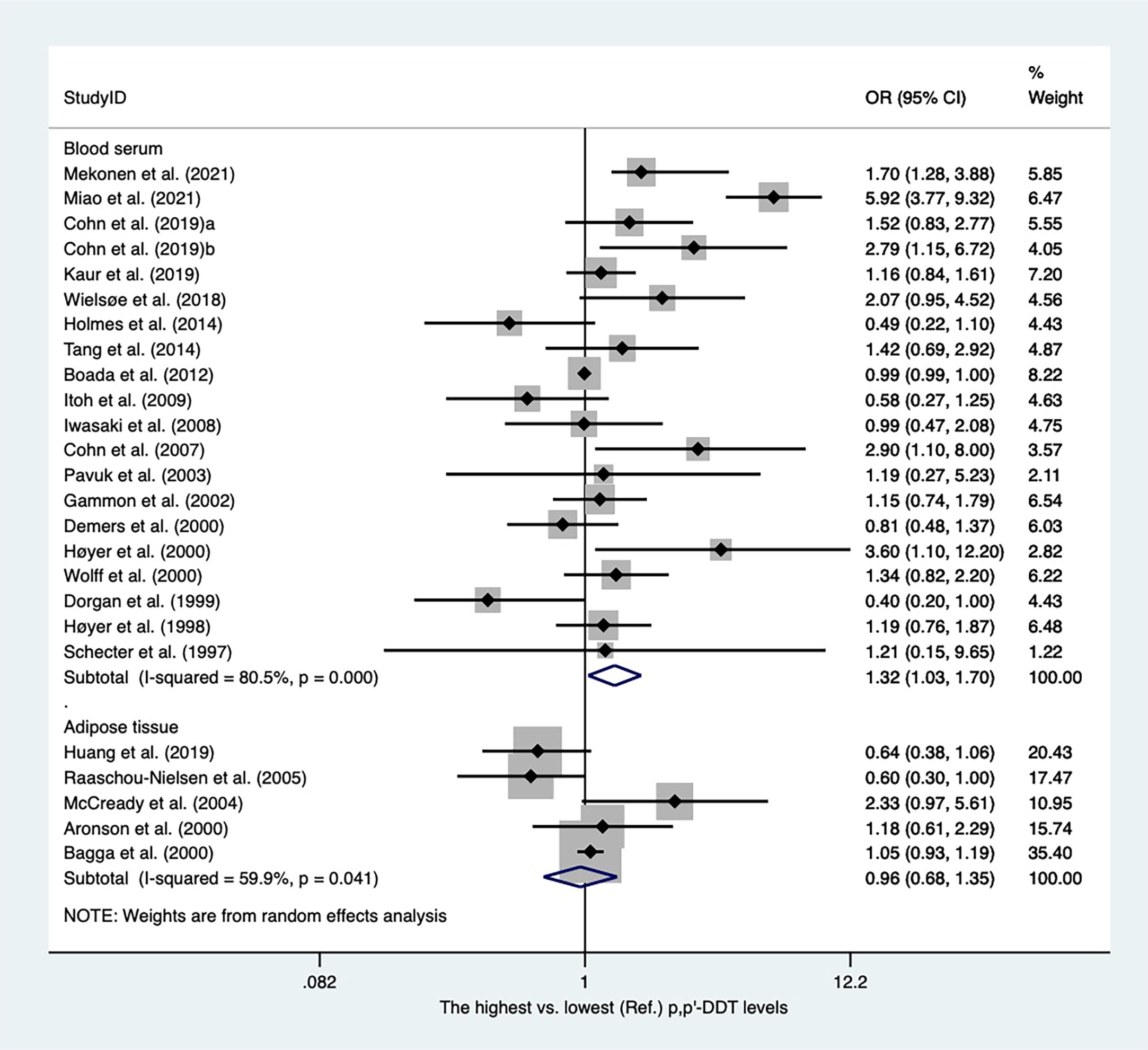

Supplement: Supplementary file 1 [file DataSheet_1.zip › Supplemetary Figures 1-24/Supplementary Figure 2.jpg]

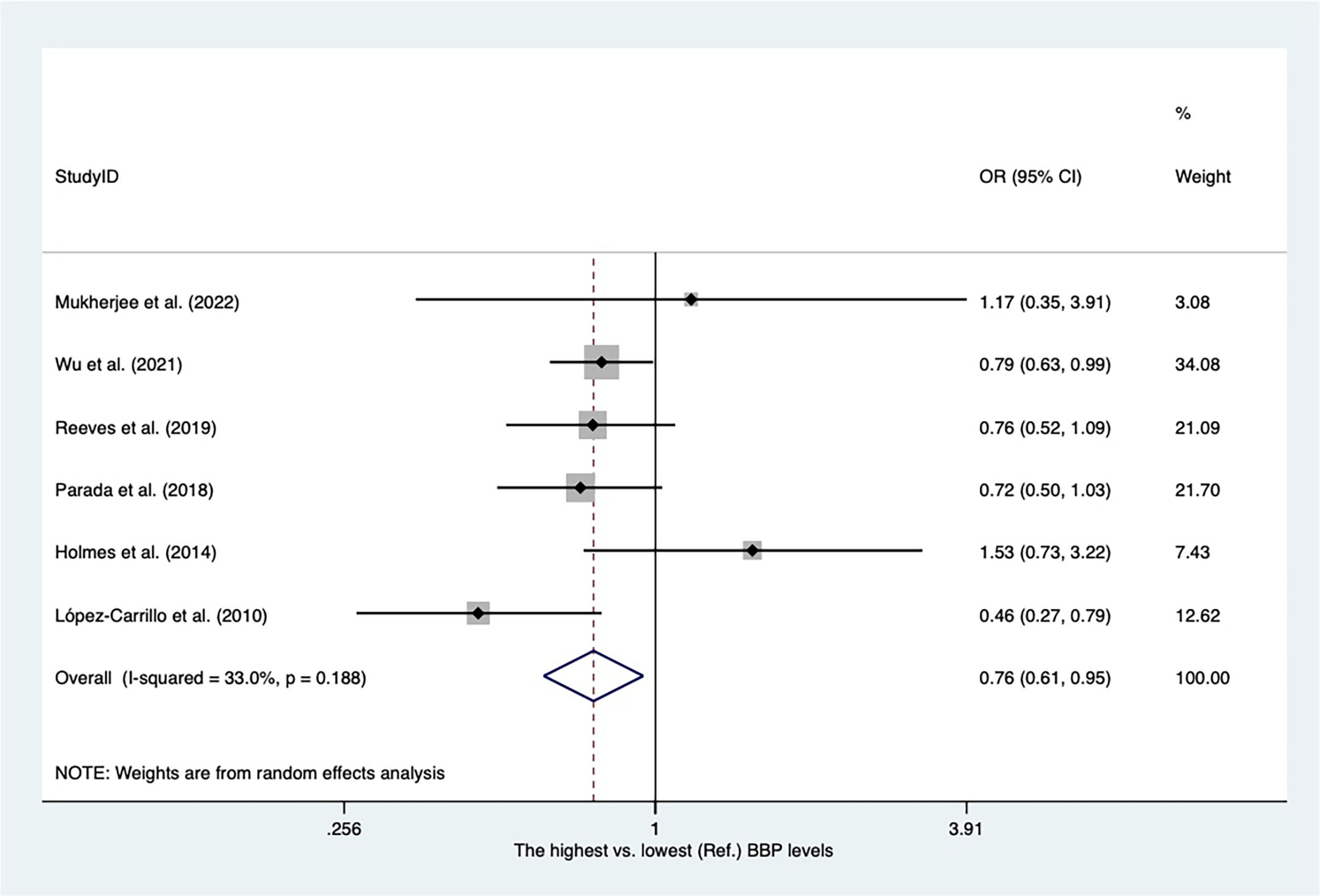

Supplement: Supplementary file 1 [file DataSheet_1.zip › Supplemetary Figures 1-24/Supplementary Figure 20.jpg]

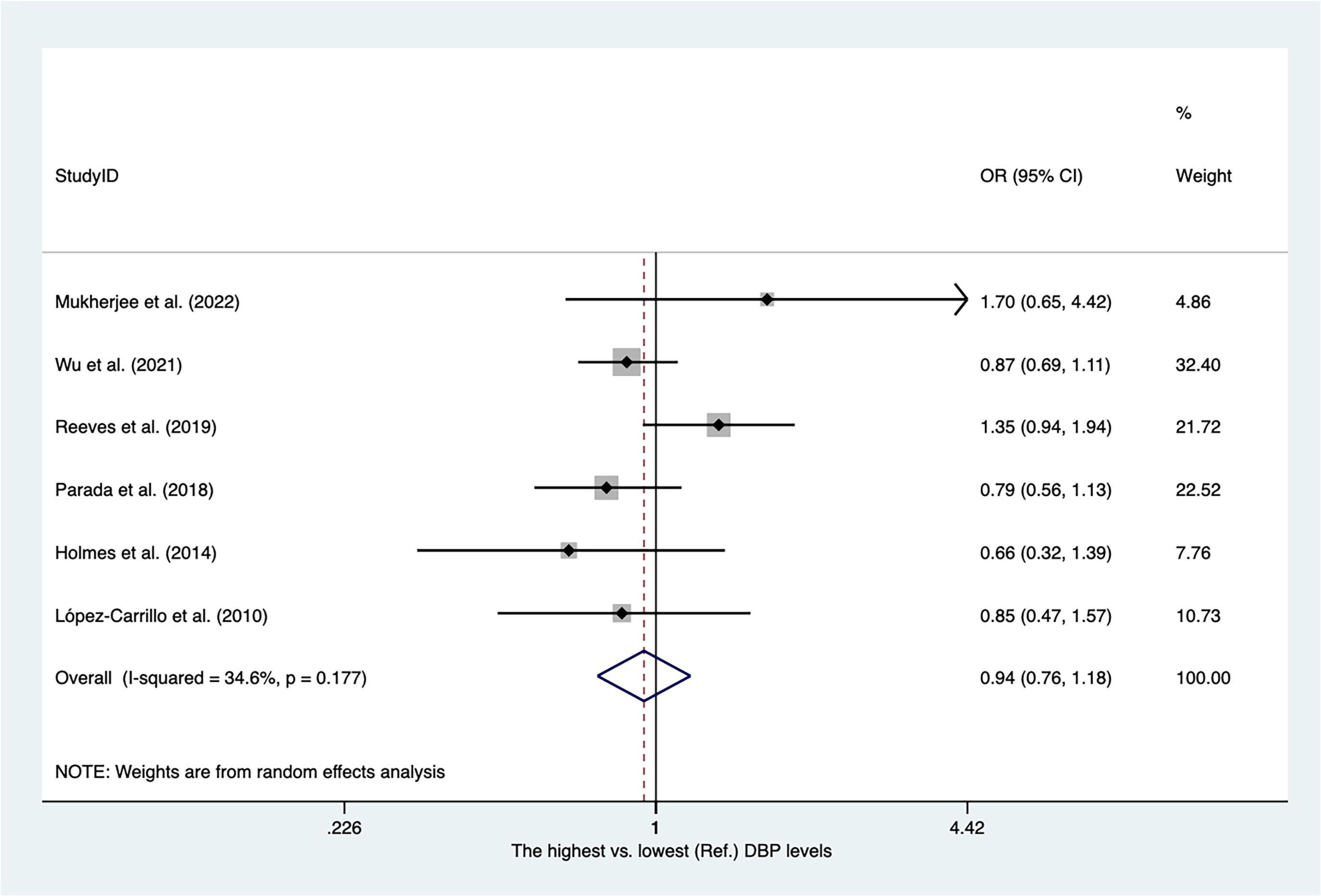

Supplement: Supplementary file 1 [file DataSheet_1.zip › Supplemetary Figures 1-24/Supplementary Figure 21.jpg]

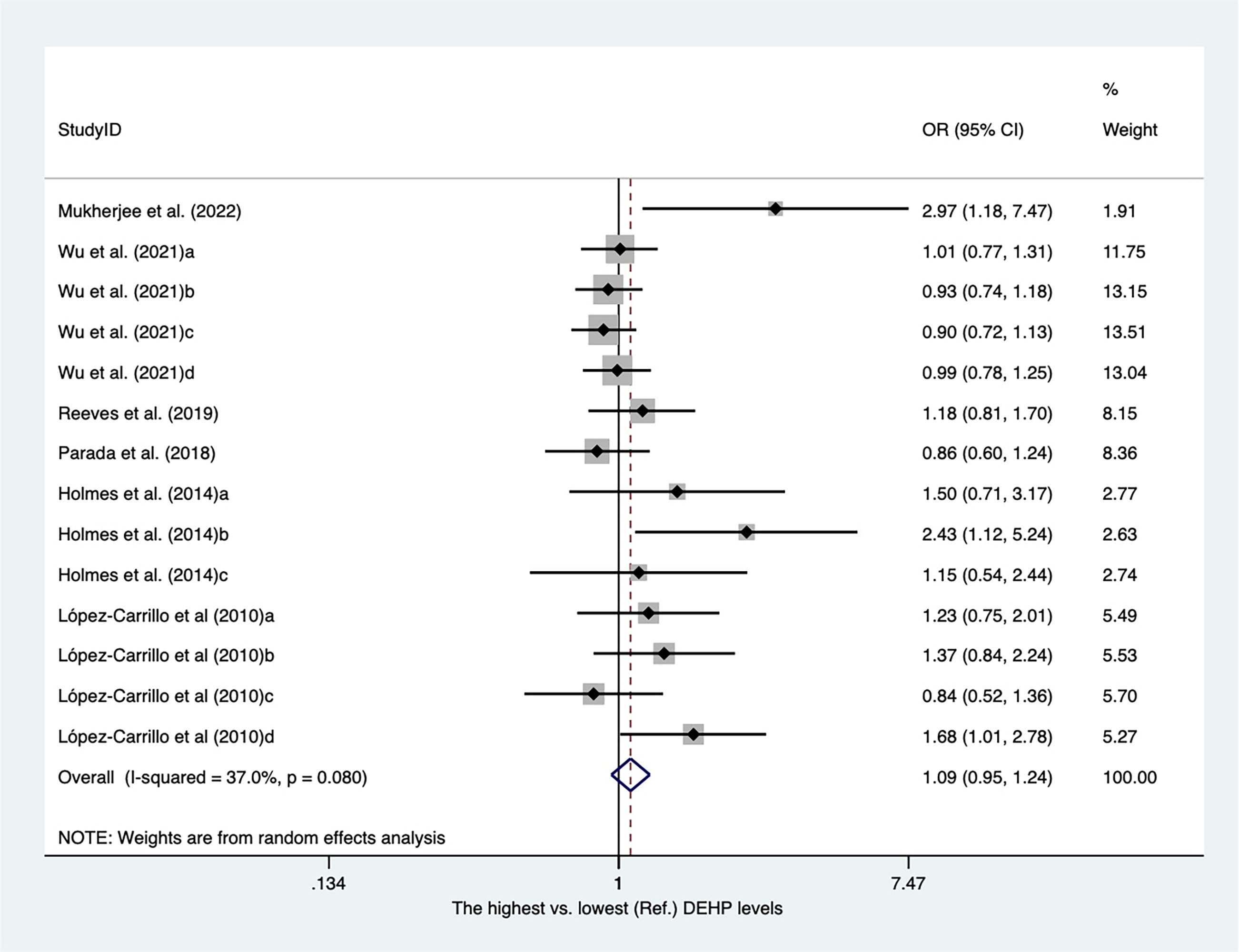

Supplement: Supplementary file 1 [file DataSheet_1.zip › Supplemetary Figures 1-24/Supplementary Figure 22.jpg]

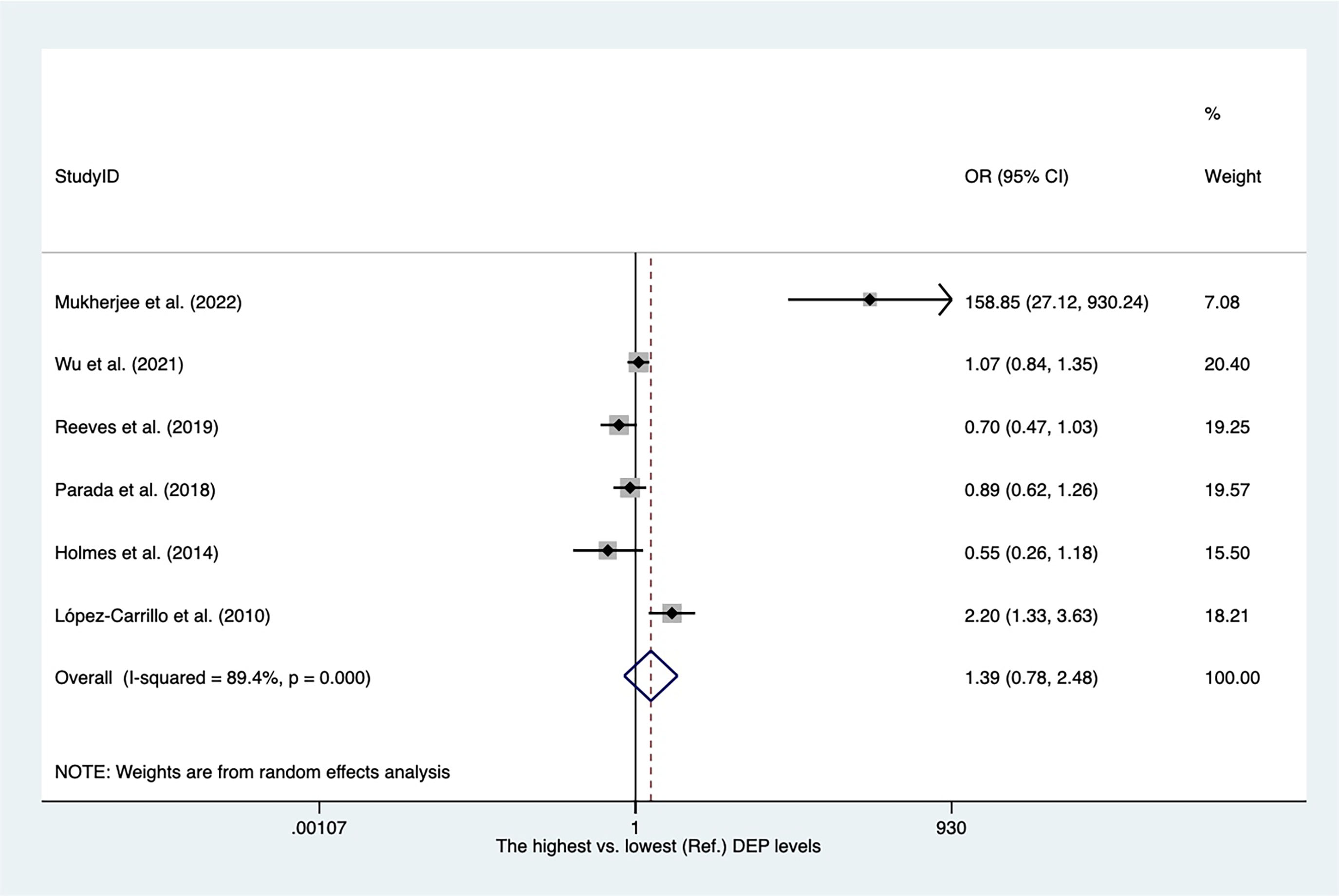

Supplement: Supplementary file 1 [file DataSheet_1.zip › Supplemetary Figures 1-24/Supplementary Figure 23.jpg]

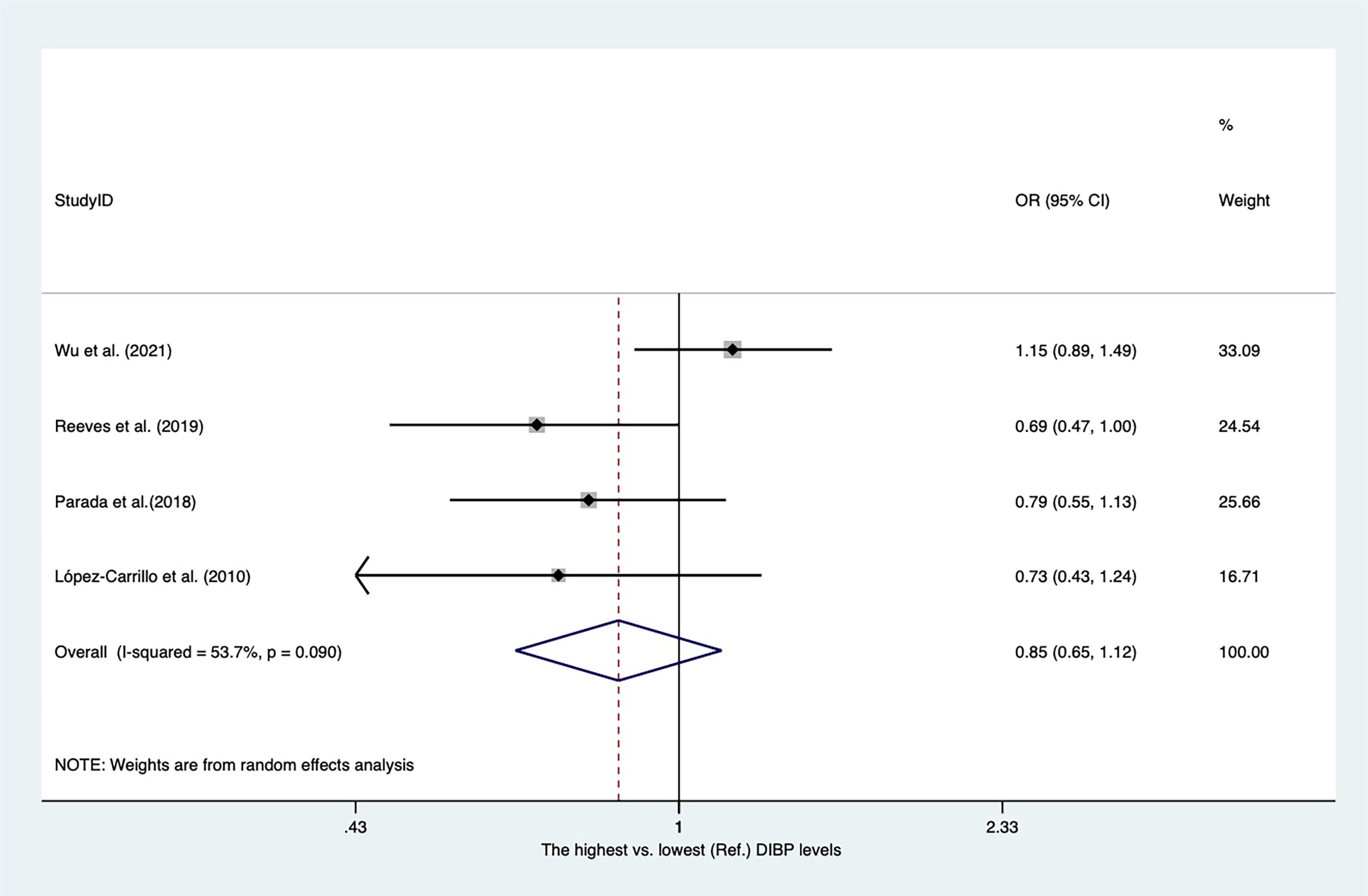

Supplement: Supplementary file 1 [file DataSheet_1.zip › Supplemetary Figures 1-24/Supplementary Figure 24.jpg]

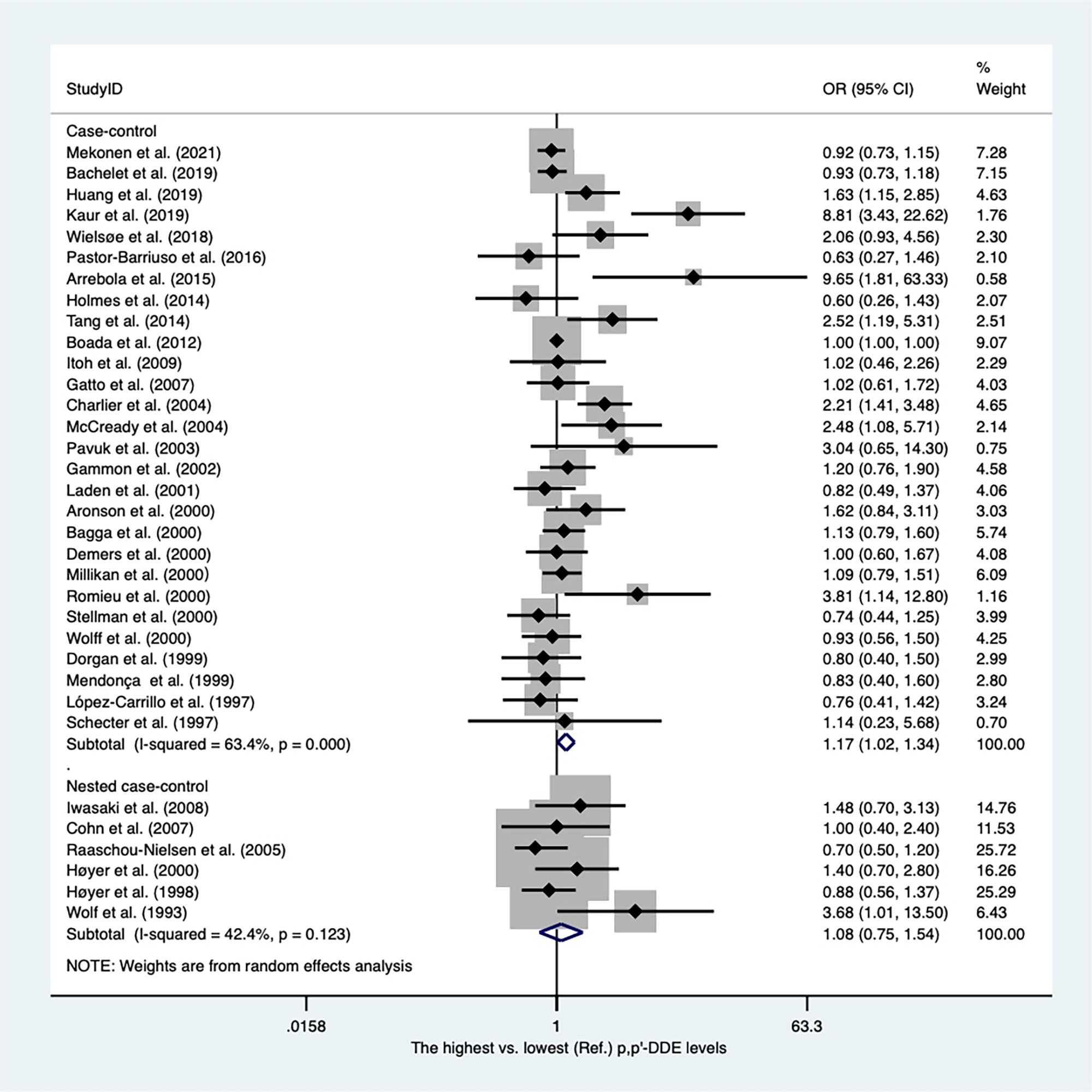

Supplement: Supplementary file 1 [file DataSheet_1.zip › Supplemetary Figures 1-24/Supplementary Figure 3.jpg]

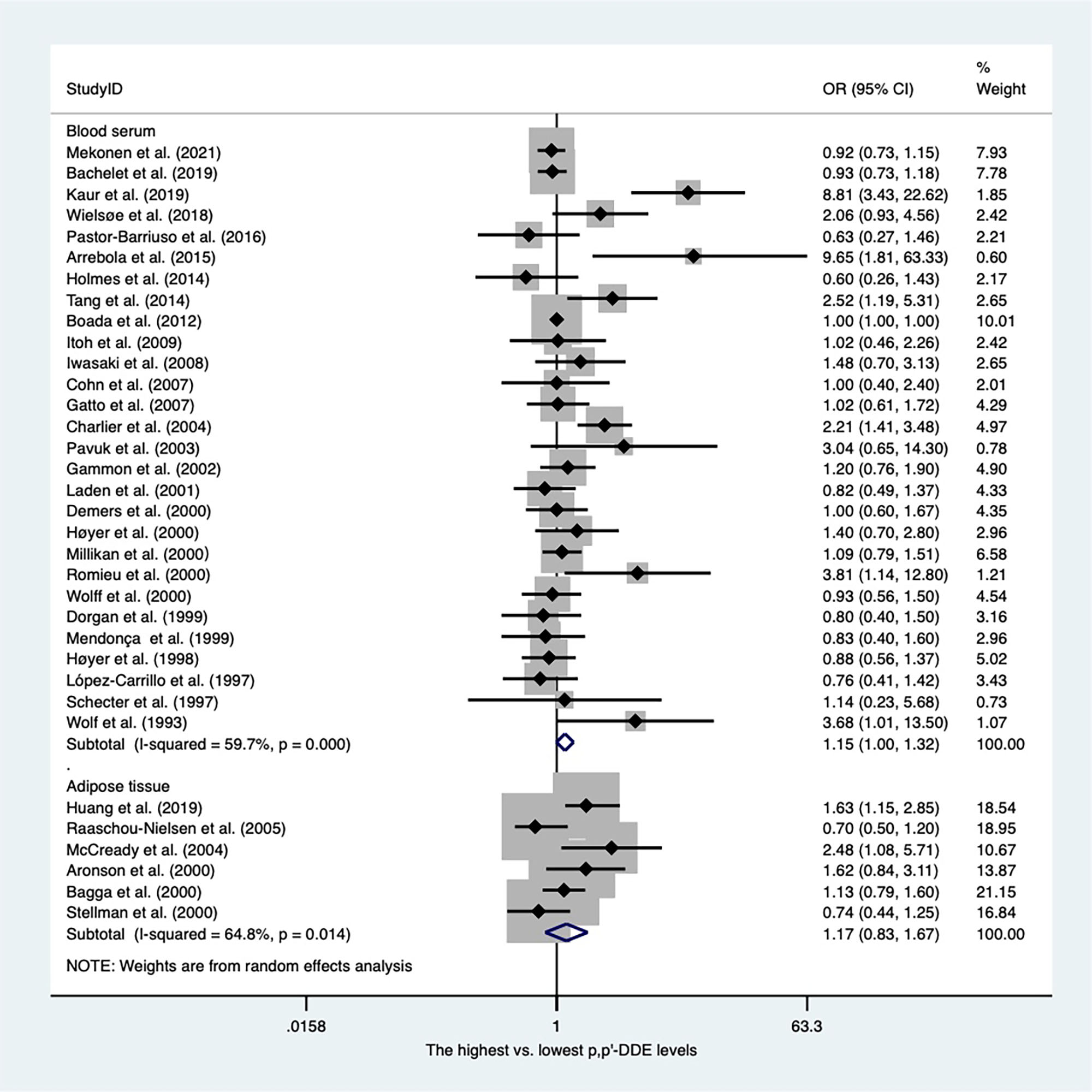

Supplement: Supplementary file 1 [file DataSheet_1.zip › Supplemetary Figures 1-24/Supplementary Figure 4.jpg]

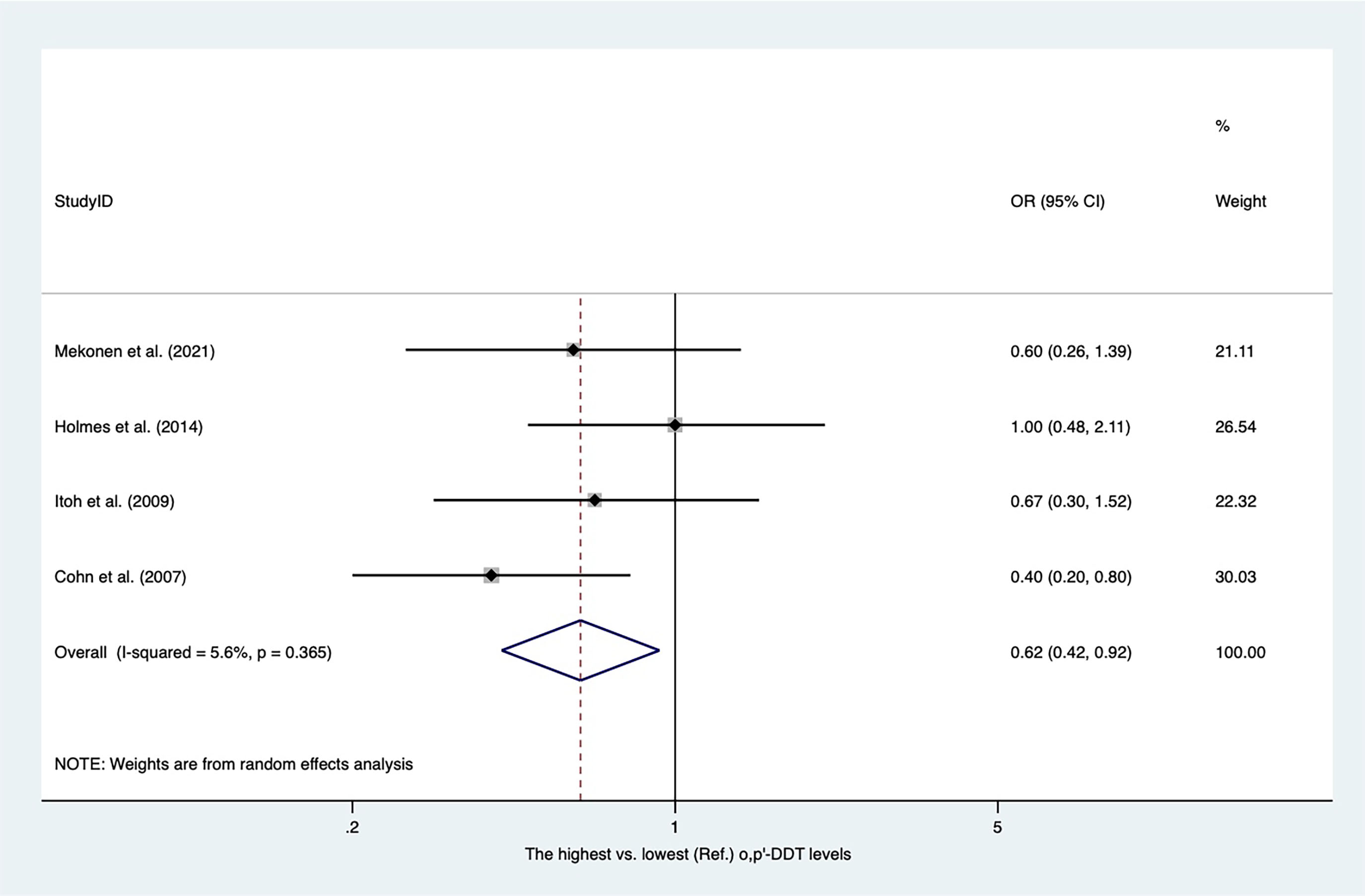

Supplement: Supplementary file 1 [file DataSheet_1.zip › Supplemetary Figures 1-24/Supplementary Figure 5.jpg]

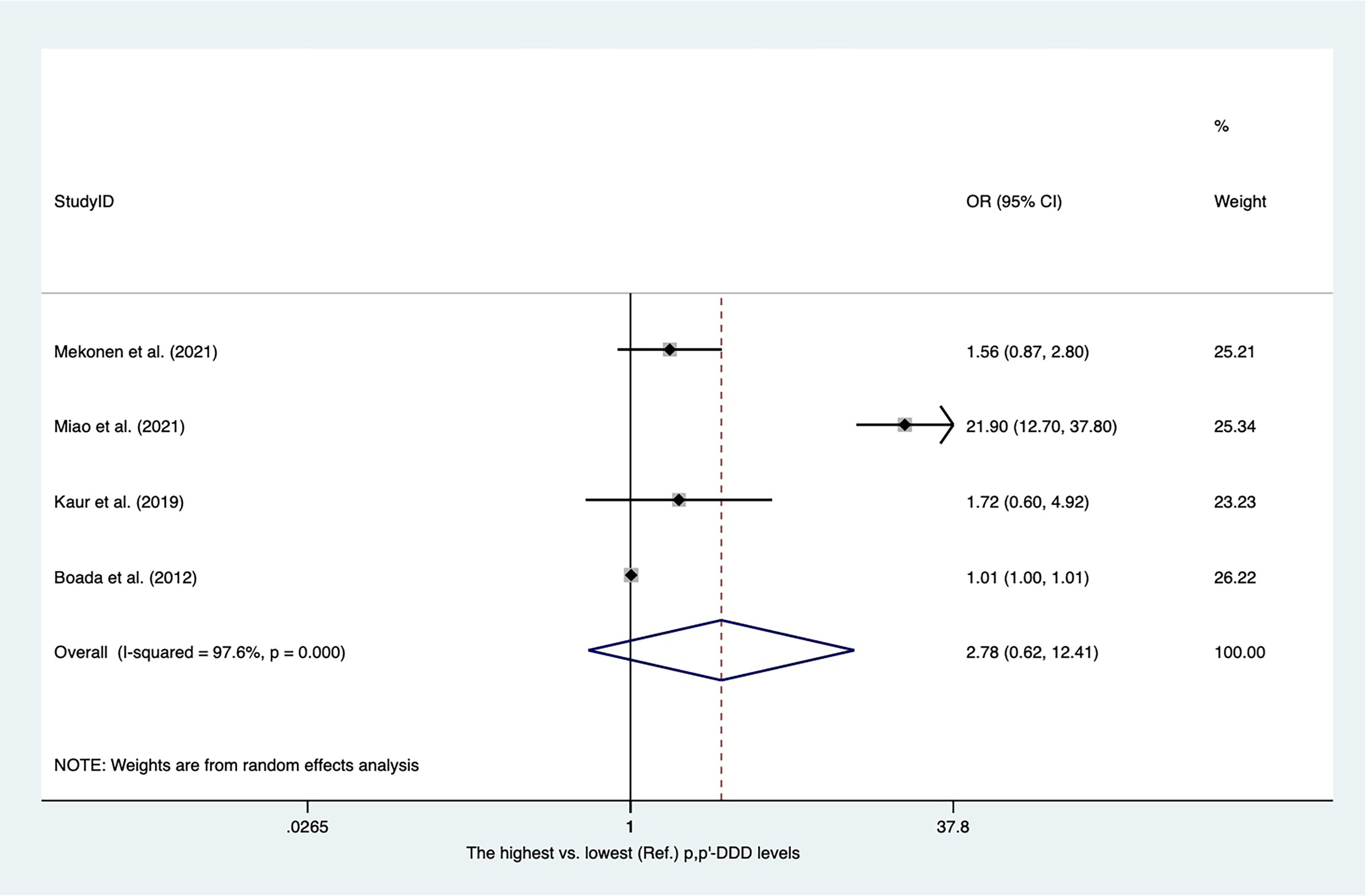

Supplement: Supplementary file 1 [file DataSheet_1.zip › Supplemetary Figures 1-24/Supplementary Figure 6.jpg]

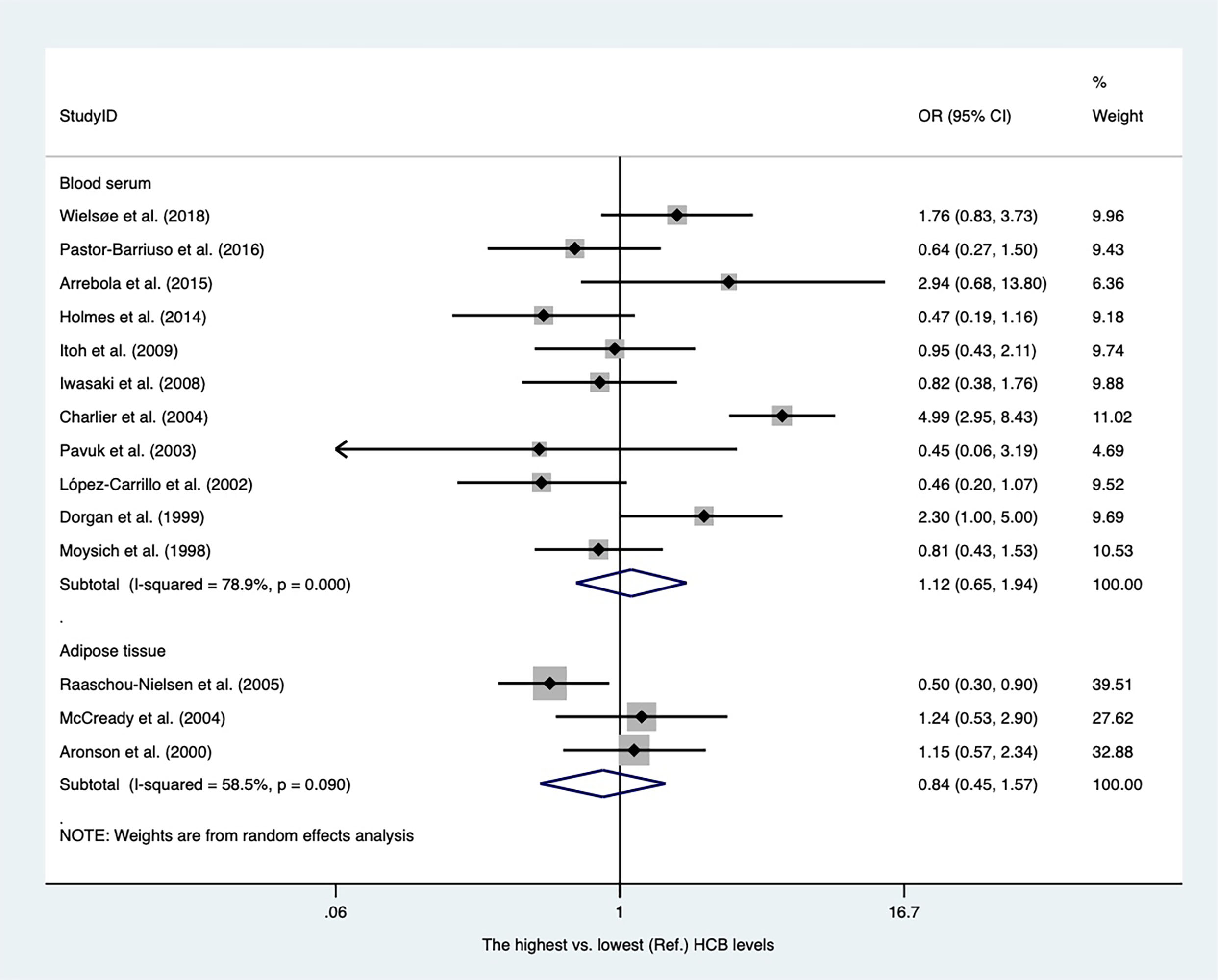

Supplement: Supplementary file 1 [file DataSheet_1.zip › Supplemetary Figures 1-24/Supplementary Figure 7.jpg]

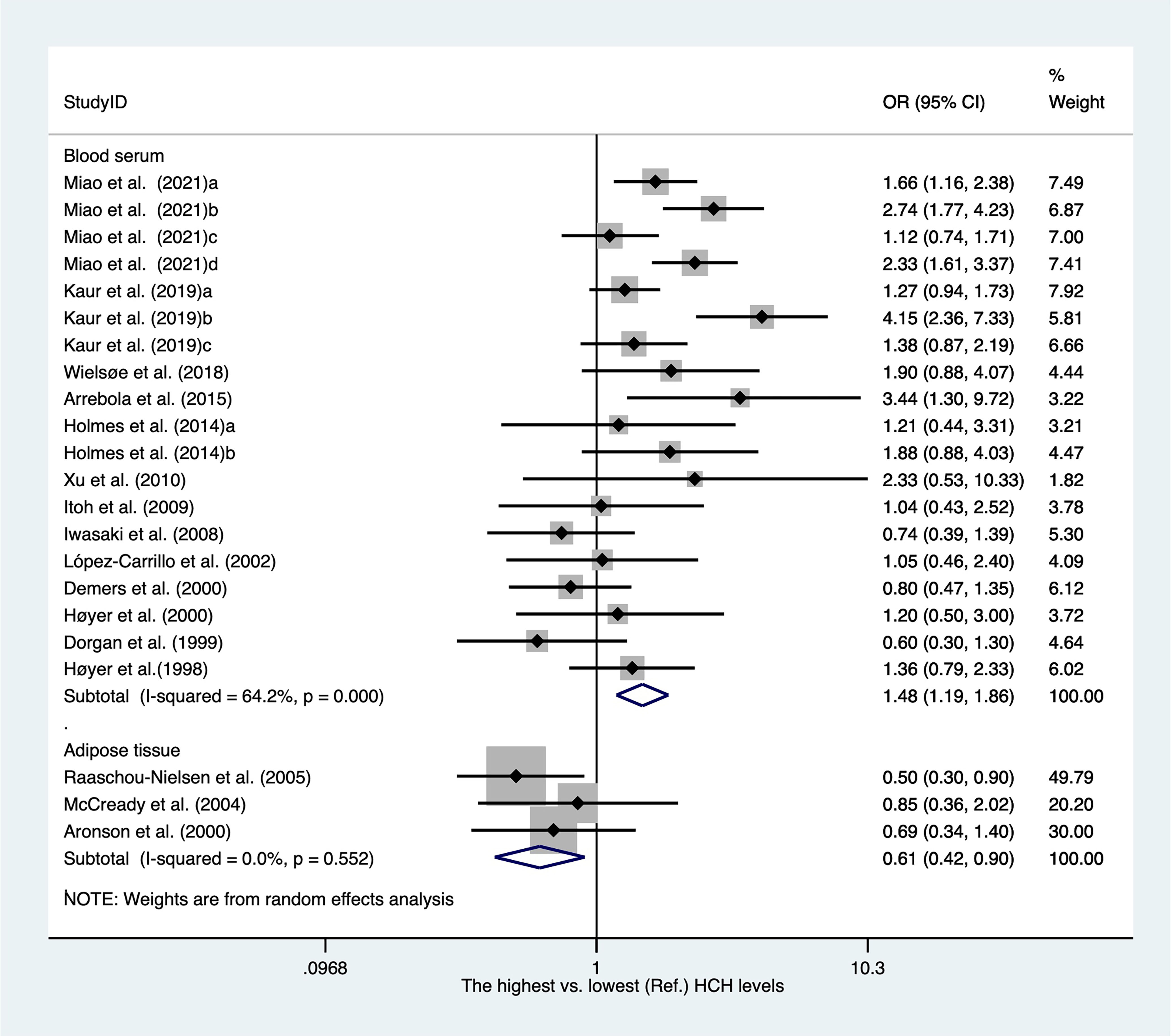

Supplement: Supplementary file 1 [file DataSheet_1.zip › Supplemetary Figures 1-24/Supplementary Figure 8.jpg]

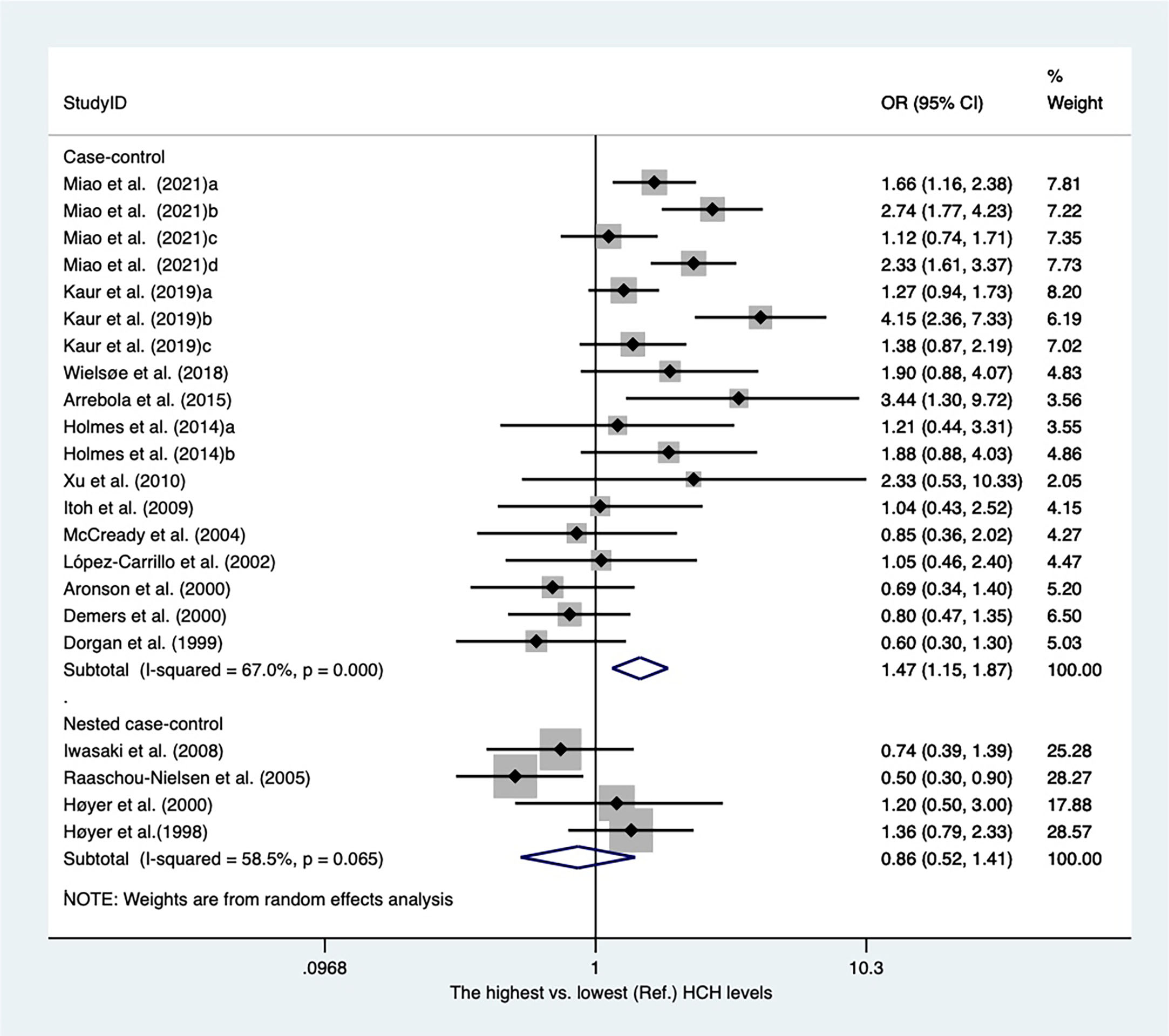

Supplement: Supplementary file 1 [file DataSheet_1.zip › Supplemetary Figures 1-24/Supplementary Figure 9.jpg]
