# Supplementary material for: Endocrine-disrupting chemicals and breast cancer: a meta-analysis
Source: Front Oncol. 2023 Nov 9;13:1282651. doi: 10.3389/fonc.2023.1282651 (PMC10665889; doi:10.3389/fonc.2023.1282651)
Supplement: Supplementary file 2 [file DataSheet_2.docx]

**Supplementary Table 1**. Search specifications and hits in the systematic literature search using three search blocks

| **Search specifications in PubMed (03.04.2023)** | Hits |
| --- | --- |
| **Exposure** | |
| General EDCs  #**1 (Pollutant*[Text Word]) OR (Pollutant*, Environmental[Text Word]) OR (Environmental Pollutant*[Text Word]) OR ("Environmental Pollutants"[Mesh]) OR (Exposure*, Environmental[Text Word]) OR (Environmental Exposure*[Text Word]) OR ("Environmental Exposure"[Mesh]) OR (Pesticide[Text Word]) OR ("Pesticides"[Mesh]) OR ("Agriculture"[Mesh]) OR (agriculture[Text Word]) OR ("Horticulture"[Mesh]) OR (Horticulture[Text Word]) OR (Plasticizer[Text Word]) OR ("Plasticizers"[Mesh]) OR (EDC[Text Word]) OR (Disruptor*, Endocrine[Text Word]) OR (Endocrine Disrupting Chemical*[Text Word]) OR (Chemical*, Endocrine Disrupting[Text Word]) OR (Disrupting Chemical, Endocrine[Text Word]) OR (Endocrine Disruptor Effect*[Text Word]) OR (Disruptor Effect*, Endocrine[Text Word]) OR (Effect*, Endocrine Disruptor[Text Word]) OR ("Endocrine Disruptor*"[Mesh]) OR (organochlorine*[Text Word]) OR (Persistent organic*[Text Word]) OR (hydrocarbon halogenated[Text Word]) OR (flame retardant[Text Word]) OR (alkylphenol*[Text Word]) OR (DDE[Text Word]) OR (DDT[Text Word]) OR (PCB[Text Word]) OR (HCB[Text Word]) OR (Hexadrin[Text Word]) OR ("Endrin"[Mesh]) OR (PBDE[Text Word]) OR (PCDD[Text Word]) OR (PCDF[Text Word]) OR (TCDD[Text Word]) OR (phthalate*[Text Word]) OR ("Hydrocarbons, Chlorinated"[Mesh]) OR (Hydrocarbons, Chlorinated[Text Word]) OR ("Polychlorinated Biphenyls"[Mesh]) OR (polychlorinated biphenyl[Text Word]) OR (1,1 dichloro 2,2 bis(4 chlorophenyl)ethylene [All Fields]) OR ("Polychlorinated Dibenzodioxins"[Mesh]) OR (polychlorinated dibenzodioxin[Text Word]) OR (phthalic acid[Text Word]) OR (polybrominated diphenyl ether [All Fields]) OR (diphenyl ether derivate [All Fields]) OR (polychlorinated biphenyl derivate [All Fields]) OR (polychlorinated dibenzofuran [All Fields])** | **884,741** |
| **Specific compounds**  **#2 ("Chlordan"[Mesh]) OR (Chlordan* [All Fields]) OR ("Dicofol"[Mesh]) OR (Dicofol [All Fields]) OR (Kepone [All Fields]) OR (Chlordecone [All Fields]) OR ("Mirex"[Mesh]) OR (mirex[Text Word]) OR (toxaphene [All Fields]) OR (camphechlor [All Fields]) OR (DDT [All Fields]) OR (clofenotane [All Fields]) OR (p p DDT [All Fields]) OR (1 1 1 2 Tetrachloro 2 2 bis [All Fields]) OR (4 chlorophenyl [All Fields]) OR (etane [All Fields]) OR (ethane [All Fields]) OR (Vinclozolin [All Fields]) OR (Maneb [All Fields]) OR (Metam [All Fields]) OR (Thiram [All Fields]) OR (Zineb [All Fields]) OR (Gamma-HCH [All Fields]) OR (Lindane [All Fields]) OR (Linuron [All Fields]) OR (Lorox [All Fields]) OR (Atrazine [All Fields]) OR (Acetochlor [All Fields]) OR (Alachlor [All Fields]) OR (Styrene [All Fields]) OR (Styrene oxide [All Fields]) OR (Hexachlorobenzene [All Fields]) OR (HCB [All Fields]) OR (Butylbenzylphthalate [All Fields]) OR (BBP [All Fields]) OR ("2 ethylhexyl" [All Fields]) OR (DEHP [All Fields]) OR ("Di n butylphthalate" [All Fields]) OR (DBP [All Fields]) OR ("4 hydroxyphenyl" [All Fields]) OR (propan* [All Fields]) OR ("4 4 isopropylidenediphenol" [All Fields]) OR (Bisphenol A [All Fields]) OR (PCB [All Fields]) OR (PCB153[All Fields]) OR (PCB169[All Fields]) OR (PCB77[All Fields]) OR (Aroclor 1242[All Fields]) OR (Aroclor 1248[All Fields]) OR (Aroclor 1254[All Fields]) OR (Aroclor 1260[All Fields]) OR (PBBs[All Fields]) OR ("Brominated Biphenyls"[All Fields]) OR ("1 2 3 7 8 Pentachlorodibenzo-p-dioxin"[All Fields]) OR ("2 3 7 8 Tetrachlorodibenzo-p-dioxin"[All Fields]) OR ("2 3 4 7 8 Pentachlorodibenzofuran"[All Fields]) OR (Tributyltin[All Fields]) OR (Tributyltin compounds[All Fields]) OR (Tributyltin oxide[All Fields]) OR ("2 propenoic acid"[All Fields]) OR (tert-butylmethacrylate[All Fields]) OR (Methoxyethylacrylate[All Fields]) OR (Phenol[All Fields]) OR (tributylstannyl[All Fields]) OR (carbony[All Fields]) OR (Stannane[All Fields]) OR (Fentin acetate[All Fields]) OR ("3 4-Dichloroaniline"[All Fields]) OR (Resorcino*[All Fields])** | **847,838** |
| #3 #1 OR #2 | **1,625,368** |
| **Outcome** | |
| **#4 (Breast Carcinoma[Text Word]) OR (Neoplasm, Human Mammary[Text Word]) OR (Carcinomas, Human Mammary[Text Word]) OR (Breast Cancer[Text Word]) OR (Breast Tumors[Text Word]) OR ("Breast Neoplasms"[Mesh])** | **439,443** |
| **Exposure and outcome** | |
| #5 **(prospective OR follow-up OR cohort OR longitudinal OR "case-cohort" OR "nested case-control" OR incidence) NOT (review[Publication Type])** | **5,214,315** |
| **#6 #3 AND #4 AND #5** | **2,669** |
| **Search specifications in Web of science (03.04.2023)** | Hits |
| **Exposure** | |
| General EDCs  **#1 TS=Pollutant* OR Pollutant*, Environmental OR Environmental Pollutant* OR Exposure*, Environmental OR Environmental Exposure* OR Pesticide* OR agriculture OR Horticulture OR Plasticizer* OR UV industrial chemical OR EDC OR Disruptor*, Endocrine OR Endocrine Disrupting Chemical* OR Chemical*, Endocrine Disrupting OR Disrupting Chemical, Endocrine OR Endocrine Disruptor Effect* OR Disruptor Effect*, Endocrine OR Effect*, Endocrine Disruptor OR organochlorine* OR Persistent organic* OR hydrocarbon halogenated OR flame retardant OR alkylphenol* OR DDE OR DDT OR PCB OR HCB OR Hexadrin OR Endrin OR PBDE OR PCDD OR PCDF OR TCDD OR phthalate* OR Hydrocarbons, Chlorinated OR polychlorinated biphenyl* OR 1,1 dichloro 2,2 bis(4 chlorophenyl)ethylene OR polychlorinated dibenzodioxin* OR phthalic acid OR polybrominated diphenyl ether OR diphenyl ether derivate OR polychlorinated biphenyl derivate OR polychlorinated dibenzofuran** | **3,302,783** |
| **Specific compounds**  **#2 TS=Chlordan* OR Dicofol OR Kepone OR Chlordecone OR mirex OR toxaphene OR camphechlor OR DDT OR clofenotane OR p p DDT OR 1 1 1 2 Tetrachloro 2 2 bis OR 4 chlorophenyl OR etane OR ethane OR Vinclozolin OR Maneb OR Metam OR Thiram OR Zineb OR Gamma-HCH OR Lindane OR Linuron OR Lorox OR Atrazine OR Acetochlor OR Alachlor OR Styrene OR Styrene oxide OR Hexachlorobenzene OR HCB OR Butylbenzylphthalate OR BBP OR 2 ethylhexyl OR DEHP OR Di n butylphthalate OR DBP OR 4 hydroxyphenyl OR propan* OR 4 4 isopropylidenediphenol OR Bisphenol A OR PCB OR PCB153 OR PCB169 OR PCB77 OR Aroclor 1242 OR Aroclor 1248 OR Aroclor 1254 OR Aroclor 1260 OR PBBs ORBrominated Biphenyls OR 1 2 3 7 8 Pentachlorodibenzo-p-dioxin OR 2 3 7 8 Tetrachlorodibenzo-p-dioxin OR 2 3 4 7 8 Pentachlorodibenzofuran OR Tributyltin OR Tributyltin compounds OR Tributyltin oxide OR 2 propenoic acid OR tert-butylmethacrylate OR Methoxyethylacrylate OR Phenol OR tributylstannyl OR carbony OR Stannane OR Fentin acetate OR 3 4-Dichloroaniline OR Resorcino*** | **827,455** |
| #3 #1 OR #2 | **1,625,368** |
| **Outcome** | |
| **#4 TS=Breast Carcinoma OR Neoplasm, Human Mammary OR Carcinomas, Human Mammary OR Breast Cancer OR Breast Tumors OR Breast Neoplasms** | **929,540** |
| **Exposure and outcome** | |
| **#5 TS=prospective OR follow-up OR cohort OR longitudinal OR case-cohort OR nested case-control OR incidence NOT review** | **5,152,860** |
| **#6 #3 AND #4 AND #5** | **3334** |
| **Search specifications in Embase (03.04.2023)** | |
| **Exposure** | |
| General EDCs  #1 'Pollutant':ab,ti,kw OR 'Pollutant, Environmental':ab,ti,kw OR 'environmental Pollutant':ab,ti,kw OR 'Exposure, Environmental':ab,ti,kw OR 'Environmental Exposure':ab,ti,kw OR 'Pesticide':ab,ti,kw OR 'Agriculture':ab,ti,kw OR 'Horticulture':ab,ti,kw OR 'Plasticizer':ab,ti,kw OR 'UV industrial chemical':ab,ti,kw OR 'EDC':ab,ti,kw OR 'Disruptor, Endocrine':ab,ti,kw OR 'Endocrine Disrupting Chemical':ab,ti,kw OR 'Chemical, Endocrine Disrupting':ab,ti,kw OR 'disrupting Chemical, Endocrine':ab,ti,kw OR 'Endocrine Disruptor Effect':ab,ti,kw OR 'Disruptor Effect, Endocrine':ab,ti,kw OR 'Effect, Endocrine Disruptor':ab,ti,kw OR 'organochlorine':ab,ti,kw OR 'Persistent organic':ab,ti,kw OR 'hydrocarbon halogenated':ab,ti,kw OR 'flame retardant':ab,ti,kw OR 'alkylphenol':ab,ti,kw OR 'DDE':ab,ti,kw OR 'DDT':ab,ti,kw OR 'PCB':ab,ti,kw OR 'HCB':ab,ti,kw OR 'Hexadrin':ab,ti,kw OR 'Endrin':ab,ti,kw OR 'PBDE':ab,ti,kw OR 'PCDD':ab,ti,kw OR 'PCDF':ab,ti,kw OR 'TCDD':ab,ti,kw OR 'phthalate':ab,ti,kw OR 'Hydrocarbons, Chlorinated':ab,ti,kw OR 'Polychlorinated Biphenyls':ab,ti,kw OR '1,1 dichloro 2,2 bis(4 chlorophenyl)ethylene':ab,ti,kw OR 'Polychlorinated Dibenzodioxinse':ab,ti,kw OR 'phthalic acid':ab,ti,kw OR 'polybrominated diphenyl ether ':ab,ti,kw OR 'pdiphenyl ether derivate':ab,ti,kw OR 'polychlorinated biphenyl derivate':ab,ti,kw OR 'polychlorinated dibenzofuran':ab,ti,kw | 222977 |
| **Specific compounds**  #2 'Chlordan':ab,ti,kw OR 'Dicofol':ab,ti,kw OR 'Kepone':ab,ti,kw OR 'Chlordecone':ab,ti,kw OR 'Mirex':ab,ti,kw OR 'toxaphene':ab,ti,kw OR 'camphechlor':ab,ti,kw OR 'clofenotane':ab,ti,kw OR 'p p DDT':ab,ti,kw OR '1 1 1 2 Tetrachloro 2 2 bis':ab,ti,kw OR '4 chloropheny':ab,ti,kw OR 'etane':ab,ti,kw OR 'ethane':ab,ti,kw OR 'Vinclozolin':ab,ti,kw OR 'Maneb':ab,ti,kw OR 'Metam':ab,ti,kw OR 'Thiram':ab,ti,kw OR 'Zineb':ab,ti,kw OR 'Gamma-HCH':ab,ti,kw OR 'GLindane':ab,ti,kw OR 'Lindane':ab,ti,kw OR 'Linuron':ab,ti,kw OR 'Lorox':ab,ti,kw OR 'Atrazine':ab,ti,kw OR 'Acetochlor':ab,ti,kw OR 'Alachlor':ab,ti,kw OR 'Styrene':ab,ti,kw OR 'tyrene oxide':ab,ti,kw OR 'Hexachlorobenzene ':ab,ti,kw OR 'HCB':ab,ti,kw OR 'Butylbenzylphthalate':ab,ti,kw OR 'BBP':ab,ti,kw OR '2 ethylhexyl':ab,ti,kw OR 'DEHP':ab,ti,kw OR 'Di n butylphthalate':ab,ti,kw OR 'DBP':ab,ti,kw OR '4 hydroxypheny':ab,ti,kw OR 'propan*':ab,ti,kw OR '4 4 isopropylidenediphenol':ab,ti,kw OR 'Bisphenol A':ab,ti,kw OR 'PCB':ab,ti,kw OR 'PCB153':ab,ti,kw OR 'PCB169':ab,ti,kw OR 'PCB77':ab,ti,kw OR 'Aroclor':ab,ti,kw OR 'Aroclor 1248':ab,ti,kw OR 'Aroclor 1254':ab,ti,kw OR 'Aroclor 1260':ab,ti,kw OR 'PBBs':ab,ti,kw OR 'Brominated Biphenyls':ab,ti,kw OR '1 2 3 7 8 Pentachlorodibenzo-p-dioxin':ab,ti,kw OR '2 3 7 8 Tetrachlorodibenzo-p-dioxin':ab,ti,kw OR '2 3 4 7 8 Pentachlorodibenzofuran':ab,ti,kw OR 'Tributyltin':ab,ti,kw OR 'Tributyltin compounds':ab,ti,kw OR 'Tributyltin oxide':ab,ti,kw OR '2 propenoic acid':ab,ti,kw OR 'tert-butylmethacrylate':ab,ti,kw OR 'Methoxyethylacrylate':ab,ti,kw OR 'Phenol':ab,ti,kw OR 'tributylstannyl':ab,ti,kw OR 'carbony':ab,ti,kw OR 'Stannane':ab,ti,kw OR 'Fentin acetate':ab,ti,kw OR '3 4-Dichloroaniline':ab,ti,kw OR 'Resorcino*':ab,ti,kw | 216715 |
| #3 #1 OR #2 | 393039 |
| **Outcome** | |
| **#4** 'Breast Carcinoma':ab,ti,kw OR 'Neoplasm, Human Mammary':ab,ti,kw OR 'Carcinomas, Human Mammary':ab,ti,kw OR 'Breast Cancer':ab,ti,kw OR 'Breast Tumors':ab,ti,kw OR 'Breast Neoplasms':ab,ti,kw | 509280 |
| **Exposure and outcome** | |
| **#5** ('prospective':it OR 'prospective':ti,ab,kw OR 'follow up':ti,ab,kw OR 'cohort':ti,ab,kw OR 'cohort':it OR 'longitudinal':ti,ab,kw OR 'case-cohort':ti,ab,kw OR 'case-cohort':it OR 'nested case-control':ti,ab,kw OR 'nested case-control':it OR 'incidence':ti,ab,kw) NOT 'review':it | 4414123 |
| **#6 #3 AND #4 AND #5** | **489** |

| **Supplementary Table 2**: Summary of papers for DDT/DDE exposure and breast cancer risk | | | | | | | | | | |
| --- | --- | --- | --- | --- | --- | --- | --- | --- | --- | --- |
| Reference | Location | Study design | N Cases/  Referents | Biospecimens | Exposure contrast | Substance | OR | 95%CI | CR | Bias |
| Mekonen et al. (2021)(1) | Ethiopia | PCC | 50/50 | serum | Median in cases versus median in referents | p,p’-DDE | 0.92 | 0.73-1.15 | 8 | 1 |
|  |  |  |  |  |  | p,p’-DDT | 1.70 | 1.28-3.88 |  |  |
|  |  |  |  |  |  | o,p’-DDT | 0.60 | 0.26-1.39 |  |  |
|  |  |  |  |  |  | p,p’-DDD | 1.56 | 0.87-2.80 |  |  |
| Miao et al. (2021)(2) | China | HCC | 313/313 | serum | Highest tertile versus lowest | p,p’-DDT | 5.92 | 3.77-9.32 | 9 | 0 |
|  |  |  |  |  |  | p,p’-DDD | 21.90 | 12.70-37.80 |  |  |
| Bachelet et al. (2019)(3) | France | PCC | 695/1055 | serum | Highest quartile versus lowest | p,p'-DDE | 0.93 | 0.73-1.18 | 8 | 0 |
| Cohn et al. (2019)a(4) | America | Nested | 153,129/  432,129 | serum | Highest tertile versus lowest | p,p'-DDT | 1.52 | 0.83-2.77 | 8 | 0 |
| Cohn et al. (2019)b(4) |  |  |  |  |  | p,p'-DDT | 2.79 | 1.15-6.72 |  |  |
| Huang et al. (2019)(5) | China | HCC | 209/163 | Adipose tissue | Highest tertile versus lowest | p,p'-DDT | 0.64 | 0.38-1.06 | 8 | 0 |
|  |  |  |  |  |  | p,p'-DDE | 1.63 | 1.15-2.85 |  |  |
| Kaur et al. (2019)(6) | India | HCC | 42/42 | serum | Median in cases versus median in referents | p,p'-DDE | 8.81 | 3.34-22.62 | 6 | 1 |
|  |  |  |  |  |  | p,p'-DDT | 1.16 | 0.84-1.61 |  |  |
|  |  |  |  |  |  | p,p'-DDD | 1.72 | 0.60-4.92 |  |  |
| Wielsøe et al. (2018)(7) | Denmark | HCC | 77/84 | serum | Highest tertile versus lowest | p,p’-DDE | 2.06 | 0.93-4.52 | 9 | 0 |
|  |  |  |  |  |  | p,p’-DDT | 2.07 | 0.95-4.52 |  |  |
| Pastor-Barriuso et al. (2016)(8) | Spain | PCC | 186/196 | Serum | Highest tertile versus lowest | p,p'-DDE | 0.63 | 0.27-1.46 | 9 | 0 |
| Arrebola et al. (2015)(9) | Tunisia | PCC | 69/54 | serum | Highest tertile versus lowest | p,p'-DDE | 9.65 | 1.81-63.33 | 10 | 0 |
| Holmes et al. (2014)(10) | America | HCC | 75/95 | serum | Median in cases versus median in referents | p,p'-DDE | 0.60 | 0.26-1.43 | 8 | 1 |
|  |  |  |  |  |  | p,p'-DDT | 0.49 | 0.22-1.10 |  |  |
|  |  |  |  |  |  | o,p'-DDT | 1.00 | 0.48-2.11 |  |  |
| Tang et al. (2014)(11) | China | PCC | 78/72 | serum | Above versus below median in referents | p,p'-DDE | 2.52 | 1.19-5.31 | 8 | 1 |
|  |  |  |  |  |  | p,p'-DDT | 1.42 | 0.69-2.92 |  |  |
| Boada et al. (2012)(12) | Spain | PCC | 121/103 | serum | Median in cases versus median in referents | p,p’-DDE | 0.999 | 0.996-1.001 | 9 | 0 |
|  |  |  |  |  |  | p,p’-DDT | 0.994 | 0.987-1.002 |  |  |
|  |  |  |  |  |  | p,p’-DDD | 1.008 | 1.001-1.015 |  |  |
| Itoh et al. (2009)(13) | Japan | HCC | 403/349 | serum | Highest quartile versus lowest | p,p’-DDT | 0.58 | 0.27-1.25 | 10 | 0 |
|  |  |  |  |  |  | p,p’-DDE | 1.02 | 0.46-2.08 |  |  |
|  |  |  |  |  |  | o,p’-DDT | 0.67 | 0.30-1.52 |  |  |
| Iwasaki et al. (2008)(14) | Japan | Nested | 139/278 | serum | Highest quartile versus lowest | p,p'-DDT | 0.99 | 0.47-2.08 | 9 | 0 |
|  |  |  |  |  |  | p,p'-DDE | 1.48 | 0.70-3.13 |  |  |
| Cohn et al. (2007)(15) | America | Nested | 129/129 | serum | Highest tertile versus lowest | p,p´-DDT | 2.90 | 1.10-8.00 | 8 | 0 |
|  |  |  |  |  |  | p,p´-DDE | 1.00 | 0.40-2.40 |  |  |
|  |  |  |  |  |  | o,p´-DDT | 0.40 | 0.20-0.80 |  |  |
| Gatto et al. (2007)(16) | America | HCC | 355/327 | serum | Highest tertile versus lowest | p,p’-DDE | 1.02 | 0.61-1.72 | 10 | 0 |
| Raaschou-Nielsen et al. (2005)(17) | Denmark | Nested | 409/409 | Adipose tissue | Highest quartile versus lowest | p,p'-DDE | 0.70 | 0.50-1.20 | 9 | 0 |
|  |  |  |  |  |  | p,p'-DDT | 0.60 | 0.30-1.00 |  |  |
| Charlier et al. (2004)(18) | Belgium | HCC | 231/290 | serum | Median in cases versus median in referents | p,p’-DDE | 2.21 | 1.41-3.48 | 10 | 0 |
| McCready et al. (2004)(19) | Canada | HCC | 70/69 | Adipose tissue | Above versus below median in referents | p,p'-DDE | 2.48 | 1.08-5.71 | 8 | 1 |
|  |  |  |  |  |  | p,p'-DDT | 2.33 | 0.97-5.61 |  |  |
| Pavuk et al. (2003)(20) | Slovakia | HCC | 22/88 | Serum | Highest quartile versus lowest | p,p'-DDE | 3.04 | 0.65-14.3 | 9 | 0 |
|  |  |  |  |  |  | p,p'-DDT | 1.19 | 0.27-5.23 |  |  |
| Gammon et al. (2002)(21) | America | HCC | 646/249 | Serum | Highest tertile versus lowest | p,p’-DDE | 1.20 | 0.76-1.90 | 10 | 0 |
|  |  |  |  |  |  | p,p'-DDT | 1.15 | 0.74-1.79 |  |  |
| Laden et al. (2001)(22) | America | Nested | 372/372 | serum | Highest tertile versus lowest | p,p’-DDE | 0.82 | 0.49-1.37 | 10 | 0 |
| Aronson et al. (2000)(23) | Canada | HCC | 217/213 | Adipose tissue | Highest quartile versus lowest | p,p'-DDE | 1.62 | 0.84-3.11 | 10 | 0 |
|  |  |  |  |  |  | p,p'-DDT | 1.18 | 0.61-2.29 |  |  |
| Bagga et al. (2000)(24) | America | HCC | 73/73 | Adipose tissue | Median in cases versus median in referents | p,p'-DDT | 1.05 | 0.93-1.19 | 7 | 1 |
|  |  |  |  |  |  | p,p'-DDE | 1.13 | 0.79-1.60 |  |  |
| Demers et al. (2000)(25) | Canada | PCC | 315/307 | serum | Highest tertile versus lowest | p,p'-DDE | 1.00 | 0.60-1.67 | 9 | 1 |
|  |  |  |  |  |  | p,p'-DDT | 0.81 | 0.48-1.37 |  |  |
| Høyer et al. (2000)(26) | Denmark | Nested | 155/274 | serum | Highest quartile versus lowest | p,p'-DDT | 3.60 | 1.10-12.2 | 9 | 0 |
|  |  |  |  |  |  | p,p'-DDE | 1.40 | 0.70-2.80 |  |  |
| Millikan et al. (2000)(27) | America | PCC | 748/659 | serum | Highest tertile versus lowest | p,p'-DDE | 1.09 | 0.79-1.15 | 9 | 0 |
| Romieu et al. (2000)(28) | Mexico | PCC | 120/165 | serum | Highest quartile versus lowest | p,p'-DDE | 3.81 | 1.14-12.80 | 9 | 0 |
| Stellman et al. (2000)(29) | America | HCC | 232/323 | Adipose tissue | Highest tertile versus lowest | p,p’-DDE | 0.74 | 0.44-1.25 | 9 | 0 |
| Wolff et al. (2000)(30) | America | HCC | 175/355 | serum | Highest tertile versus lowest | p,p'-DDE | 0.93 | 0.56-1.50 | 8 | 0 |
|  |  |  |  |  |  | p,p'-DDT | 1.34 | 0.82-2.20 |  |  |
| Dorgan et al. (1999)(31) | Columbia | PCC | 105/208 | serum | Highest tertile versus lowest | p,p'-DDT | 0.40 | 0.20-1.00 | 8 | 0 |
|  |  |  |  |  |  | p,p'-DDE | 0.80 | 0.40-1.50 |  |  |
| Mendonça et al. (1999)(32) | Brazil | HCC | 177/350 | serum | Highest tertile versus lowest | p,p'-DDE | 0.83 | 0.40-1.60 | 8 | 0 |
| Høyer et al. (1998)(33) | Denmark | Nested | 240/447 | serum | Highest quartile versus lowest | p,p'-DDT | 1.19 | 0.76-1.87 | 9 | 0 |
|  |  |  |  |  |  | p,p'-DDE | 0.88 | 0.56-1.37 |  |  |
| López-Carrillo et al. (1997)(34) | Mexico | HCC | 141/141 | serum | Highest tertile versus lowest | p,p'-DDE | 0.76 | 0.41-1.42 | 9 | 0 |
| Schecter et al. (1997)(35) | Vietnam | HCC | 21/21 | serum | Highest tertile versus lowest | p,p'-DDE | 1.14 | 0.23-5.68 | 7 | 1 |
|  | Vietnam | HCC | 21/21 | serum | Highest tertile versus lowest | p,p'-DDT | 1.21 | 0.15-9.65 |  |  |
| Wolf et al. (1993)(36) | America | Nested | 58/171 | serum | Highest tertile versus lowest | p,p’-DDE | 3.68 | 1.01-13.50 | 9 | 1 |

Abbreviations: HCC, hospital-based case-control; PCC, population-based case-control; OR, odds ratio; 95%CI, 95% confidence interval; CR: Completeness of reporting; p,p'-DDE, 2,2-Bis(4-chlorophenyl)-1,1-dichloroethylene; p,p'-DDT, 1-chloro-4-[2,2,2-trichloro-1-(4-chlorophenyl)ethyl]benzene; o,p’-DDT, 1-methoxy-2-[2,2,2-trichloro-1-(4-methoxyphenyl)ethyl]benzene; p,p’-DDD, p,p'-TDE 2,2-Bis(4-chlorophenyl)-1,1-dichloroethane.

a: postmenopausal women; b: premenopausal women.

| **Supplementary Table 3**: Summary of papers for HCB exposure and breast cancer risk | | | | | | | | | | |
| --- | --- | --- | --- | --- | --- | --- | --- | --- | --- | --- |
| Reference | Location | Study design | N Cases/  Referents | Biospecimens | Exposure contrast | Substance | OR | 95%CI | CR | Bias |
| Wielsøe et al. (2018)(7) | Denmark | HCC | 77/84 | serum | Highest tertile versus lowest | HCB | 1.76 | 0.83-3.73 | 9 | 0 |
| Pastor-Barriuso et al. (2016)(8) | Spain | PCC | 186/196 | Serum | Highest tertile versus lowest | HCB | 0.64 | 0.27-1.5 | 9 | 0 |
| Arrebola et al. (2015)(9) | Tunisia | PCC | 69/54 | Serum | Highest quartile versus lowest | HCB | 2.94 | 0.68-13.8 | 10 | 0 |
| Holmes et al. (2014)(10) | America | HCC | 75/95 | Serum | Median in cases versus median in referents | HCB | 0.47 | 0.19-1.16 | 8 | 1 |
| Itoh et al. (2009)(13) | Japan | HCC | 403/349 | Serum | Highest quartile versus lowest | HCB | 0.95 | 0.43-2.11 | 10 | 0 |
| Iwasaki et al. (2008)(14) | Japan | Nested | 139/278 | serum | Highest quartile versus lowest | HCB | 0.82 | 0.38-1.76 | 10 | 0 |
| Raaschou-Nielsen et al. (2005)(17) | Danish | Nested | 409/409 | Adipose tissue | Highest quartile versus lowest | HCB | 0.50 | 0.30-0.90 | 9 | 0 |
| Charlier et al. (2004)(18) | Belgium | HCC | 231/290 | Serum | Median in cases versus median in referents | HCB | 4.99 | 2.95-8.43 | 10 | 0 |
| McCready et al. (2004)(19) | Canada | HCC | 70/69 | Adipose tissue | Above versus below median in referents | HCB | 1.24 | 0.53-2.90 | 8 | 1 |
| Pavuk et al. (2003)(20) | Slovakia | HCC | 22/88 | serum | Highest tertile versus lowest | HCB | 0.45 | 0.06-3.19 | 9 | 0 |
| López-Carrillo et al. (2002)(37) | Mexico | HCC | 95/95 | serum | Highest quartile versus lowest | HCB | 0.46 | 0.20-1.07 | 8 | 0 |
| Aronson et al. (2000)(23) | Canada | HCC | 217/213 | Adipose tissue | Highest quartile versus lowest | HCB | 1.15 | 0.57-2.34 | 10 | 0 |
| Dorgan et al. (1999)(31) | Columbia | PCC | 105/208 | serum | Highest quartile versus lowest | HCB | 2.30 | 1.00-5.00 | 8 | 0 |
| Moysich et al. (1998)(38) | America | PCC | 154/192 | serum | Highest tertile versus lowest | HCB | 0.81 | 0.43-1.53 | 10 | 0 |

Abbreviations: HCC, hospital-based case-control; PCC, population-based case-control; OR, odds ratio; 95%CI, 95% confidence interval; CR: Completeness of reporting; HCB, Hexachlorobenzene.

| **Supplementary Table 4**: Summary of papers for HCH exposure and breast cancer risk | | | | | | | | | | |
| --- | --- | --- | --- | --- | --- | --- | --- | --- | --- | --- |
| Reference | Location | Study design | N Cases/  Referents | Biospecimens | Exposure contrast | Substance | OR | 95%CI | CR | Bias |
| Miao et al. (2021)a(2) | China | HCC | 313/313 | serum | Highest tertile versus lowest | ɑ-HCH | 1.66 | 1.16-2.38 | 9 | 0 |
| Miao et al. (2021)b(2) |  |  |  |  |  | β-HCH | 2.74 | 1.77-4.23 |  |  |
| Miao et al. (2021)c(2) |  |  |  |  |  | ɤ-HCH | 1.12 | 0.74-1.71 |  |  |
| Miao et al. (2021)d(2) |  |  |  |  |  | δ-HCH | 2.33 | 1.61-3.37 |  |  |
| Kaur et al. (2019)a(6) | India | HCC | 42/42 | serum | Above versus below median in referents | α‑HCH | 1.27 | 0.94-1.73 | 6 | 1 |
| Kaur et al. (2019)b(6) |  |  |  |  |  | β‑HCH | 4.15 | 2.36-7.33 |  |  |
| Kaur et al. (2019)c(6) |  |  |  |  |  | γ‑HCH | 1.38 | 0.87-2.19 |  |  |
| Wielsøe et al. (2018)(7) | Denmark | HCC | 77/84 | serum | Highest tertile versus lowest | β-HCH | 1.90 | 0.88-4.07 | 9 | 0 |
| Arrebola et al. (2015)(9) | Tunisia | PCC | 69/54 | serum | Highest quartile versus lowest | β-HCH | 3.44 | 1.30-9.72 | 10 | 0 |
| Holmes et al. (2014)a(10) | America | HCC | 75/95 | serum | Median in cases versus median in referents | β-HCH | 1.21 | 0.44-3.31 | 8 | 1 |
| Holmes et al. (2014)b(10) |  |  |  |  |  | r-HCH | 1.88 | 0.88-4.03 |  |  |
| Xu et al. (2010)(39) | America | PCC | 128/4109 | serum | Highest quartile versus lowest | β-HCH | 2.33 | 0.53-10.33 | 9 | 0 |
| Itoh et al. (2009)(13) | Japan | HCC | 403/349 | serum | Highest quartile versus lowest | β-HCH | 1.04 | 0.43-2.52 | 10 | 0 |
| Iwasaki et al. (2008)(14) | Japan | Nested | 139/278 | serum | Highest quartile versus lowest | β-HCH | 0.74 | 0.39-1.39 | 10 | 0 |
| Raaschou-Nielsen et al. (2005)(17) | Danish | Nested | 409/409 | Adipose tissue | Highest quartile versus lowest | β-HCH | 0.50 | 0.30-0.90 | 9 | 0 |
| McCready et al. (2004)(19) | Canada | HCC | 70/69 | Adipose tissue | Above versus below median in referents | β-HCH | 0.85 | 0.36-2.02 | 8 | 1 |
| López-Carrillo et al. (2002)(37) | Mexico | HCC | 95/95 | serum | Highest quartile versus lowest | β-HCH | 1.05 | 0.46-2.40 | 8 | 0 |
| Aronson et al. (2000)(23) | Canada | HCC | 217/213 | Adipose tissue | Highest quartile versus lowest | β-HCH | 0.69 | 0.34-1.40 | 10 | 0 |
| Demers et al. (2000)(25) | Canada | PCC | 315/307 | serum | Highest tertile versus lowest | β-HCH | 0.80 | 0.47-1.35 | 8 | 0 |
| Høyer et al. (2000)(26) | Denmark | Nested | 155/274 | serum | Highest quartile versus lowest | β-HCH | 1.20 | 0.50-3.00 | 9 | 0 |
| Dorgan et al. (1999)(31) | Columbia | PCC | 105/208 | serum | Highest quartile versus lowest | β-HCH | 0.60 | 0.30-1.30 | 8 | 0 |
| Høyer et al.(1998) (33) | Denmark | Nested | 240/447 | serum | Highest quartile versus lowest | β-HCH | 1.36 | 0.79-2.33 | 9 | 0 |

Abbreviations: HCC, hospital-based case-control; PCC, population-based case-control; OR, odds ratio; 95%CI, 95% confidence interval; CR: Completeness of reporting;

# ɑ-HCH, alpha-hexachlorocyclohexane; β-HCH, beta-hexachlorocyclohexane; γ‑HCH, gamma-hexachlorocyclohexane; δ-HCH, delta-hexachlorocyclohexane.

| **Supplementary Table 5**: Summary of papers for other pesticides exposure and breast cancer risk | | | | | | | | | | |
| --- | --- | --- | --- | --- | --- | --- | --- | --- | --- | --- |
| Reference | Location | Study design | N Cases/  Referents | Biospecimens | Exposure contrast | Substance | OR | 95%CI | CR | Bias |
| Mekonen et al. (2021)(1) | Ethiopian | PCC | 50/50 | serum | Median in cases versus median in referents | ɤ-Chlordane | 1.98 | 1.12-3.50 | 8 | 1 |
|  |  |  |  |  |  | aldrin | 0.86 | 0.58-1.27 |  |  |
|  |  |  |  |  |  | dieldrin | 0.78 | 0.33-1.83 |  |  |
| Miao et al. (2021)(2) | China | HCC | 313/313 | serum | Highest tertile versus lowest | ɑ-Chlordane | 3.45 | 2.44-5.33 | 9 | 0 |
|  |  |  |  |  |  | ɤ-Chlordane | 4.48 | 2.89-6.92 |  |  |
|  |  |  |  |  |  | Aldrin | 0.95 | 0.67-1.33 |  |  |
|  |  |  |  |  |  | Dieldrin | 1.57 | 1.02-2.38 |  |  |
| Kaur et al. (2019)(6) | India | HCC | 42/42 | serum | Median in cases versus median in referents | Aldrin | 1.05 | 0.85-1.30 | 6 | 1 |
|  |  |  |  |  |  | Dieldrin | 11.74 | 3.93-35.09 |  |  |
| Wielsøe et al. (2018)(7) | Denmark | HCC | 77/84 | serum | Highest tertile versus lowest | Mirex | 1.58 | 0.73-3.44 | 9 | 0 |
|  |  |  |  |  |  | Cis-Nonachlor | 2.07 | 0.96-4.47 |  |  |
|  |  |  |  |  |  | Oxychlordane | 1.90 | 0.88-4.07 |  |  |
| Holmes et al. (2014)(10) | America | HCC | 75/95 | serum | Median in cases versus median in referents | Mirex | 0.65 | 0.28-1.55 | 8 | 1 |
|  |  |  |  |  |  | trans-Nonachlor | 0.65 | 0.26-1.66 |  |  |
|  |  |  |  |  |  | Oxychlordane | 0.91 | 0.35-2.35 |  |  |
| Boada et al. (2012)(12) | Spain | PCC | 121/103 | serum | Median in cases versus median in referents | Aldrin | 1.03 | 0.99-1.07 | 9 | 0 |
|  |  |  |  |  |  | Dieldrin | 1.00 | 0.96-1.05 |  |  |
| Xu et al. (2010)(39) | America | PCC | 63/4109 | serum | Highest quartile versus lowest | Dieldrin | 1.04 | 0.26-4.16 | 9 | 0 |
|  |  |  |  |  |  | trans-Nonachlor | 2.60 | 0.65-10.35 |  |  |
|  |  |  |  |  |  | Oxychlordane | 2.55 | 0.73-8.83 |  |  |
| Itoh et al. (2009)(13) | Japan | HCC | 403/349 | serum | Highest quartile versus lowest | Mirex | 0.40 | 0.19-0.84 | 10 | 0 |
|  |  |  |  |  |  | trans-Nonachlor | 0.49 | 0.22-1.06 |  |  |
|  |  |  |  |  |  | cis-Nonachlor | 0.41 | 0.19-0.91 |  |  |
|  |  |  |  |  |  | Oxychlordane | 0.65 | 0.31-1.38 |  |  |
| Raaschou-Nielsen et al. (2005)(17) | Denmark | Nested | 409/409 | Adipose tissue | Highest quartile versus lowest | Dieldrin | 0.90 | 0.50-1.60 | 9 | 0 |
|  |  |  |  |  |  | trans-Nonachlor | 0.70 | 0.50-1.20 |  |  |
|  |  |  |  |  |  | cis-Nonachlor | 1.50 | 0.80-2.70 |  |  |
|  |  |  |  |  |  | Oxychlordane | 0.50 | 0.30-0.90 |  |  |
| McCready et al. (2004)(19) | Canada | HCC | 70/69 | Adipose tissue | Above versus below median in referents | Mirex | 1.17 | 0.54-2.55 | 9 | 0 |
|  |  |  |  |  |  | trans-Nonachlor | 0.97 | 0.44-2.18 |  |  |
|  |  |  |  |  |  | cis-Nonachlor | 1.04 | 0.46-2.36 |  |  |
|  |  |  |  |  |  | Oxychlordane | 0.99 | 0.44-2.21 |  |  |
| Gammon et al. (2002)(21) | America | HCC | 646/249 | Serum | Highest tertile versus lowest | chlordane | 0.98 | 0.62-1.55 | 10 | 0 |
|  |  |  |  |  |  | Dieldrin | 1.37 | 0.69-2.72 |  |  |
| Aronson et al. (2000)(23) | Canada | HCC | 217/213 | Adipose tissue | Highest quartile versus lowest | Mirex | 1.18 | 0.59-2.38 | 10 | 0 |
|  |  |  |  |  |  | trans-Nonachlor | 0.78 | 0.40-1.53 |  |  |
|  |  |  |  |  |  | cis-Nonachlor | 0.80 | 0.41-1.53 |  |  |
|  |  |  |  |  |  | Oxychlordane | 0.59 | 0.31-1.16 |  |  |
| Demers et al. (2000)(25) | Canada | PCC | 315/307 | serum | Highest tertile versus lowest | trans-Nonachlor | 1.20 | 0.68-2.13 | 9 | 1 |
|  |  |  |  |  |  | Oxychlordane | 1.47 | 0.83-2.62 |  |  |
| Wolff et al. (2000)(30) | America | HCC | 175/355 | serum | Highest tertile versus lowest | trans-Nonachlor | 0.73 | 0.43-1.20 | 8 | 0 |
| Dorgan et al. (1999)(31) | Columbia | PCC | 105/208 | serum | Highest quartile versus lowest | Dieldrin | 0.70 | 0.30-1.30 | 8 | 0 |
| Høyer et al. (1998)(33) | Denmark | Nested | 240/447 | serum | Highest quartile versus lowest | Dieldrin | 2.05 | 1.17-3.57 | 9 | 0 |
| Moysich et al. (1998)(38) | America | PCC | 154/192 | serum | Highest tertile versus lowest | Mirex | 1.37 | 0.78-2.39 | 10 | 0 |

Abbreviations: HCC, hospital-based case-control; PCC, population-based case-control; OR, odds ratio; 95%CI, 95% confidence interval; CR: Completeness of reporting.

| **Supplementary Table 6**: Summary of papers for PCBs exposure and breast cancer risk | | | | | | | | | | |
| --- | --- | --- | --- | --- | --- | --- | --- | --- | --- | --- |
| Reference | Location | Study design | N Cases/  Referents | Biospecimens | Exposure contrast | Substance | OR | 95%CI | CR | Bias |
| Parada et al. (2021)(40) | Carolina | PCC | 748/659 | serum | Highest tertile versus lowest | PCB99 | 1.40 | 1.00-1.90 | 11 | 0 |
|  |  |  |  |  |  | PCB118 | 1.10 | 0.79-1.50 |  |  |
|  |  |  |  |  |  | PCB74 | 1.30 | 0.95-1.80 |  |  |
|  |  |  |  |  |  | PCB138 | 1.10 | 0.78-1.50 |  |  |
|  |  |  |  |  |  | PCB153 | 0.96 | 0.69-1.30 |  |  |
|  |  |  |  |  |  | PCB180 | 0.98 | 0.72-1.30 |  |  |
| Bachelet et al. (2019)(3) | France | PCC | 695/1055 | serum | Highest quartile versus lowest | PCB153 | 0.75 | 0.57-0.97 | 8 | 0 |
| Huang et al. (2019)(5) | China | HCC | 209/163 | Adipose tissue | Highest tertile versus lowest | PCB52 | 1.24 | 0.78-2.00 | 8 | 0 |
|  |  |  |  |  |  | PCB101 | 2.41 | 1.43-4.06 |  |  |
|  |  |  |  |  |  | PCB138 | 2.66 | 1.54-4.61 |  |  |
|  |  |  |  |  |  | PCB118 | 4.50 | 2.53-8.00 |  |  |
|  |  |  |  |  |  | PCB153 | 7.88 | 4.13-15.02 |  |  |
|  |  |  |  |  |  | PCB180 | 1.77 | 1.05-2.97 |  |  |
| Wielsøe et al. (2018)(7) | Denmark | HCC | 77/84 | serum | Highest tertile versus lowest | PCB99 | 2.00 | 0.86-4.67 | 9 | 0 |
|  |  |  |  |  |  | PCB101 | 1.26 | 056-2.81 |  |  |
|  |  |  |  |  |  | PCB105 | 1.54 | 0.71-3.33 |  |  |
|  |  |  |  |  |  | PCB118 | 1.48 | 0.70-3.11 |  |  |
|  |  |  |  |  |  | PCB138 | 2.50 | 1.07-5.85 |  |  |
|  |  |  |  |  |  | PCB153 | 2.69 | 1.18-6.14 |  |  |
|  |  |  |  |  |  | PCB156 | 2.06 | 0.93-4.56 |  |  |
|  |  |  |  |  |  | PCB170 | 2.43 | 1.08-5.48 |  |  |
|  |  |  |  |  |  | PCB180 | 1.65 | 0.78-3.47 |  |  |
|  |  |  |  |  |  | PCB183 | 2.39 | 1.04-5.49 |  |  |
|  |  |  |  |  |  | PCB187 | 2.19 | 0.99-4.82 |  |  |
| Pastor-Barriuso et al. (2016)(8) | Spain | PCC | 186/196 | Serum | Highest tertile versus lowest | PCB138 | 1.64 | 0.78-3.46 | 9 | 0 |
|  |  |  |  |  |  | PCB153 | 1.33 | 0.64-2.75 |  |  |
|  |  |  |  |  |  | PCB180 | 1.09 | 0.49-2.43 |  |  |
| Arrebola et al. (2015)(9) | Tunisia | PCC | 69/54 | serum | Highest quartile versus lowest | PCB138 | 2.99 | 0.75-13.42 | 10 | 0 |
|  |  |  |  |  |  | PCB153 | 1.25 | 0.31-5.11 |  |  |
|  |  |  |  |  |  | PCB180 | 1.11 | 0.26-4.64 |  |  |
| Holmes et al. (2014)(10) | America | HCC | 75/95 | serum | Median in cases versus median in referents | PCB153 | 0.55 | 0.22-1.41 | 8 | 1 |
|  |  |  |  |  |  | PCB180 | 0.43 | 0.17-1.09 | 8 | 0 |
| Cohn et al. (2012)(41) | America | Nested | 123/117 | serum | Highest tertile versus lowest | PCB187 | 0.35 | 0.11-1.14 | 8 | 0 |
| Recio-Vega et al. (2011)(42) | Mexican | HCC | 70/70 | Serum | Median in cases versus median in referents | PCB52 | 1.21 | 0.63-2.31 | 9 | 1 |
|  |  |  |  |  |  | PCB101 | 1.19 | 0.61-2.35 |  |  |
|  |  |  |  |  |  | PCB105 | 1.66 | 0.89-3.09 |  |  |
|  |  |  |  |  |  | PCB118 | 3.18 | 1.20-8.42 |  |  |
|  |  |  |  |  |  | PCB138 | 3.56 | 1.41-9.01 |  |  |
|  |  |  |  |  |  | PCB153 | 1.36 | 0.67-2.73 |  |  |
|  |  |  |  |  |  | PCB170 | 5.15 | 1.74-15.20 |  |  |
|  |  |  |  |  |  | PCB180 | 7.32 | 1.89-28.40 |  |  |
|  |  |  |  |  |  | PCB187 | 1.94 | 1.00-3.77 |  |  |
| Raaschou-Nielsen et al. (2005)(17) | Denmark | Nested | 409/409 | Adipose tissue | Highest quartile versus lowest | PCB99 | 1.10 | 0.70-1.90 | 9 | 0 |
|  |  |  |  |  |  | PCB118 | 0.90 | 0.60-1.40 |  |  |
|  |  |  |  |  |  | PCB153 | 1.10 | 0.60-1.80 |  |  |
|  |  |  |  |  |  | PCB156 | 0.90 | 0.60-1.50 |  |  |
|  |  |  |  |  |  | PCB170 | 1.10 | 0.70-1.80 |  |  |
|  |  |  |  |  |  | PCB183 | 1.30 | 0.80-2.00 |  |  |
|  |  |  |  |  |  | PCB187 | 1.20 | 0.80-2.00 |  |  |
| Charlier et al. (2004)(43) | Belgium | PCC | 60/60 | serum | Median in cases versus median in referents | PCB52 | 0.95 | 0.74-1.20 | 10 | 0 |
|  |  |  |  |  |  | PCB101 | 1.00 | 0.77-1.30 |  |  |
|  |  |  |  |  |  | PCB138 | 1.20 | 0.88-1.50 |  |  |
|  |  |  |  |  |  | PCB153 | 1.80 | 1.40-2.50 |  |  |
|  |  |  |  |  |  | PCB180 | 1.10 | 0.76-1.50 |  |  |
| McCready et al. (2004)(19) | Canada | HCC | 70/69 | Adipose tisse | Above versus below median in referents | PCB99 | 2.40 | 0.95-6.04 | 9 | 0 |
|  |  |  |  |  |  | PCB105 | 2.50 | 1.02-6.13 |  |  |
|  |  |  |  |  |  | PCB118 | 1..71 | 0.69-4.21 |  |  |
|  |  |  |  |  |  | PCB138 | 1.07 | 0.48-2.38 |  |  |
|  |  |  |  |  |  | PCB153 | 1.03 | 0.44-2.38 |  |  |
|  |  |  |  |  |  | PCB156 | 1.17 | 0.53-2.58 |  |  |
|  |  |  |  |  |  | PCB170 | 0.86 | 0.38-1.91 |  |  |
|  |  |  |  |  |  | PCB180 | 0.77 | 0.35-1.70 |  |  |
|  |  |  |  |  |  | PCB183 | 1.48 | 0.65-3.33 |  |  |
|  |  |  |  |  |  | PCB187 | 1.09 | 0.50-2.38 |  |  |
| Demers et al. (2002)(44) | Canada | PCC | 314/523 | serum | Highest quartile versus lowest | PCB99 | 1.33 | 0.86-2.07 | 9 | 0 |
|  |  |  |  |  |  | PCB118 | 1.60 | 1.01-2.53 |  |  |
|  |  |  |  |  |  | PCB138 | 1.18 | 0.75-1.85 |  |  |
|  |  |  |  |  |  | PCB153 | 1.22 | 0.78-1.92 |  |  |
|  |  |  |  |  |  | PCB156 | 1.80 | 1.11-2.94 |  |  |
|  |  |  |  |  |  | PCB170 | 1.46 | 0.90-2.37 |  |  |
|  |  |  |  |  |  | PCB180 | 1.17 | 0.70-1.93 |  |  |
|  |  |  |  |  |  | PCB183 | 1.35 | 0.84-2.16 |  |  |
|  |  |  |  |  |  | PCB187 | 1.33 | 0.83-2.13 |  |  |
| Gammon et al. (2002)(21) | America | PCC | 646/249 | serum | Highest tertile versus lowest | PCB118 | 0.93 | 0.60-1.43 | 10 | 0 |
|  |  |  |  |  |  | PCB138 | 0.96 | 0.63-1.48 |  |  |
|  |  |  |  |  |  | PCB180 | 0.95 | 0.62-1.46 |  |  |
|  |  |  |  |  |  | PCB153 | 0.86 | 0.56-1.32 |  |  |
| Laden et al. (2001)(22) | America | Neasted | 155/274 | serum | Highest tertile versus lowest | PCB118 | 0.69 | 0.39-1.22 | 10 | 0 |
|  |  |  |  |  |  | PCB153 | 0.83 | 0.47-1.48 |  |  |
|  |  |  |  |  |  | PCB180 | 0.98 | 0.55-1.75 |  |  |
| Aronson et al. (2000)(23) | Canada | HCC | 217/213 | Adipose tissue | Highest quartile versus lowest | PCB99 | 1.92 | 0.95-3.86 | 10 | 0 |
|  |  |  |  |  |  | PCB105 | 3.17 | 1.51-6.68 |  |  |
|  |  |  |  |  |  | PCB118 | 2.31 | 1.11-4.78 |  |  |
|  |  |  |  |  |  | PCB138 | 1.56 | 0.80-3.06 |  |  |
|  |  |  |  |  |  | PCB153 | 1.04 | 0.51-2.11 |  |  |
|  |  |  |  |  |  | PCB170 | 1.15 | 0.60-2.22 |  |  |
|  |  |  |  |  |  | PCB156 | 1.35 | 0.68-2.69 |  |  |
|  |  |  |  |  |  | PCB180 | 1.27 | 0.66-2.46 |  |  |
|  |  |  |  |  |  | PCB183 | 1.27 | 0.66-2.45 |  |  |
|  |  |  |  |  |  | PCB187 | 1.26 | 0.66-2.40 |  |  |
| Demers et al. (2000)(25) | Canada | PCC | 315/307 | serum | Highest tertile versus lowest | PCB153 | 1.28 | 0.74-2.19 | 9 | 1 |
| Høyer et al. (2000)(26) | Denmark | Neasted | 155/274 | serum | Highest quartile versus lowest | PCB118 | 1.90 | 0.90-3.90 | 9 | 0 |
|  |  |  |  |  |  | PCB153 | 1.30 | 0.60-2.60 |  |  |
|  |  |  |  |  |  | PCB180 | 0.80 | 0.40-2.20 |  |  |
| Holford et al. (2000)(45) | America | HCC | 304/186 | Adipose tissue | Highest tertile versus lowest | PCB138 | 1.04 | 0.94-1.16 | 8 | 0 |
|  |  |  |  |  |  | PCB74 | 0.93 | 0.84-1.04 |  |  |
|  |  |  |  |  |  | PCB153 | 0.87 | 0.78-0.98 |  |  |
|  |  |  |  |  |  | PCB118 | 1.04 | 0.96-1.12 |  |  |
|  |  |  |  |  |  | PCB156 | 0.79 | 0,64-0.99 |  |  |
|  |  |  |  |  |  | PCB170 | 0.85 | 0.65-1.11 |  |  |
|  |  |  |  |  |  | PCB180 | 1.14 | 1.00-1.29 |  |  |
|  |  |  |  |  |  | PCB183 | 1.82 | 1.12-2.98 |  |  |
|  |  |  |  |  |  | PCB187 | 1.11 | 0.90-1.37 |  |  |
| Stellman et al. (2000)(29) | America | HCC | 232/323 | Adipose tissue | Highest tertile versus lowest | PCB156 | 1.50 | 0.90-2.50 | 9 | 0 |
|  |  |  |  |  |  | PCB183 | 2.00 | 1.20-3.40 |  |  |
| Zheng et al. (2000)(46) | America | HCC | 304/186 | Adipose tissue | Highest tertile versus lowest | PCB118 | 0.60 | 0.40-2.20 | 8 | 0 |
|  |  |  |  |  |  | PCB74 | 0.70 | 0.50-1.20 |  |  |
| Dorgan et al. (1999)(31) | Columbia | PCC | 105/208 | serum | Highest quartile versus lowest | PCB118 | 1.00 | 0.50-2.20 |  |  |
|  |  |  |  |  |  | PCB138 | 1.20 | 0.60-2.40 | 8 | 0 |

Abbreviations: HCC, hospital-based case-control; PCC, population-based case-control; OR, odds ratio; 95%CI, 95% confidence interval; CR: Completeness of reporting; PCB, Polychlorinated biphenyl.

| **Supplementary Table 7**: Summary of papers for phthalates exposure and breast cancer risk | | | | | | | | | | |
| --- | --- | --- | --- | --- | --- | --- | --- | --- | --- | --- |
| Reference | Location | Study design | N Cases/  Referents | Biospecimens | Exposure contrast | Substance | OR | 95%CI | CR | Bias |
| Mukherjee et al. (2022)(47) | India | HCC | 90/81 | urine | Median in cases versus median in referents | BBP | 1.17 | 0.35-3.91 | 9 | 1 |
|  |  |  |  |  |  | DBP | 1.70 | 0.65-4.42 |  |  |
|  |  |  |  |  |  | DEHP | 2.97 | 1.18-7.47 |  |  |
|  |  |  |  |  |  | DEP | 158.85 | 27.12-930.24 |  |  |
| Wu et al. (2021)(48) | America | Nested | 1030/1032 | urine | Highest tertile versus lowest | MBzP | 0.79 | 0.63-0.99 | 9 | 0 |
|  |  |  |  |  |  | MBP | 0.87 | 0.69-1.11 |  |  |
|  |  |  |  |  |  | MEHP | 1.01 | 0.77-1.31 |  |  |
|  |  |  |  |  |  | MEHHP | 0.93 | 0.74-1.18 |  |  |
|  |  |  |  |  |  | MEOHP | 0.90 | 0.72-1.13 |  |  |
|  |  |  |  |  |  | MECPP | 0.99 | 0.78-1.25 |  |  |
|  |  |  |  |  |  | MEP | 1.07 | 0.84-1.35 |  |  |
|  |  |  |  |  |  | MiBP | 1.15 | 0.89-1.49 |  |  |
| Reeves et al. (2019)(49) | America | Nested | 419/838 | urine | Highest quartile versus lowest | MBzP | 0.76 | 0.52-1.09 | 9 | 0 |
|  |  |  |  |  |  | DBP | 1.35 | 0.94-1.94 |  |  |
|  |  |  |  |  |  | DEHP | 1.18 | 0.81-1.70 |  |  |
|  |  |  |  |  |  | MEP | 0.70 | 0.47-1.03 |  |  |
|  |  |  |  |  |  | DiBP | 0.69 | 0.47-1.00 |  |  |
| Parada et al. (2018)(50) | America | PCC | 710/598 | urine | Highest tertile versus lowest | MBzP | 0.72 | 0.50-1.03 | 9 | 0 |
|  |  |  |  |  |  | MnBP | 0.79 | 0.56-1.13 |  |  |
|  |  |  |  |  |  | DEHP | 0.86 | 0.60-1.24 |  |  |
|  |  |  |  |  |  | MEP | 0.89 | 0.62-1.26 |  |  |
|  |  |  |  |  |  | MiBP | 0.79 | 0.55-1.13 |  |  |
| Holmes et al. (2014)(10) | America | PCC | 75/95 | urine | Median in cases versus median in referents | MBzP | 1.53 | 0.73-3.22 | 8 | 1 |
|  |  |  |  |  |  | MBP | 0.66 | 0.32-1.39 |  |  |
|  |  |  |  |  |  | MEHHP | 1.50 | 0.71-3.17 |  |  |
|  |  |  |  |  |  | MEHP | 2.43 | 1.12-5.24 |  |  |
|  |  |  |  |  |  | MEOHP | 1.15 | 0.54-2.44 |  |  |
|  |  |  |  |  |  | MEP | 0.55 | 0.26-1.18 |  |  |
| López-Carrillo et al. (2010)(37) | Mexico | PCC | 233/221 | urine | Highest quartile versus lowest | MBzP | 0.46 | 0.27-0.79 | 8 | 0 |
|  |  |  |  |  |  | MBP | 0.85 | 0.47-1.57 |  |  |
|  |  |  |  |  |  | MEHP | 1.23 | 0.75-2.01 |  |  |
|  |  |  |  |  |  | MEHHP | 1.37 | 0.84-2.24 |  |  |
|  |  |  |  |  |  | MEOHP | 0.84 | 0.52-1.36 |  |  |
|  |  |  |  |  |  | MECPP | 1.68 | 1.01-2.78 |  |  |
|  |  |  |  |  |  | MEP | 2.20 | 1.33-3.63 |  |  |
|  |  |  |  |  |  | MiBP | 0.73 | 0.43-1.24 |  |  |

Abbreviations: HCC, hospital-based case-control; PCC, population-based case-control; OR, odds ratio; 95%CI, 95% confidence interval; CR: Completeness of reporting; PCB, Polychlorinated biphenyl; BBP, benzyl butyl phthalate; DBP, dibutyl phthalate; DEP, diethyl phthalate; DEHP, di(2-ethylhexyl) phthalate; DIBP, diisobutyl phthalate.

BBP: MBzP,

DBP: MBP, MnBP,

DEP: MEP,

DEHP: MEHP, MEHHP, MEOHP, MECPP,

DIBP: MiBP,

| **Supplementary Table 8**: Summary of papers for PFAS exposure and breast cancer risk | | | | | | | | | | |
| --- | --- | --- | --- | --- | --- | --- | --- | --- | --- | --- |
| Reference | Location | Study design | N Cases/  Referents | Biospecimens | Exposure contrast | Substance | OR | 95%CI | CR | Bias |
| Chang et al. (2023)(51) | America | Nested | 621/621 | serum | Highest quartile versus lowest | PFOS | 1.12 | 0.79-1.59 | 8 | 0 |
|  |  |  |  |  |  | PFOA | 1.06 | 0.75-1.50 |  |  |
| Velarde et al. (2022)(52) | Philippines | HCC | 75/75 | serum | Highest quartile versus lowest | PFHxA | 2.66 | 0.95-7.66 | 9 | 0 |
|  |  |  |  |  |  | PFHpA | 0.44 | 0.12-1.43 |  |  |
|  |  |  |  |  |  | PFHxS | 1.22 | 0.43-3.45 |  |  |
|  |  |  |  |  |  | PFOS | 2.38 | 0.81-7.31 |  |  |
|  |  |  |  |  |  | PFOA | 0.44 | 0.14-1.36 |  |  |
|  |  |  |  |  |  | PFNA | 1.29 | 0.40-4.10 |  |  |
|  |  |  |  |  |  | PFDA | 9.26 | 2.54-45.10 |  |  |
|  |  |  |  |  |  | PFUnDA | 3.98 | 1.29-13.49 |  |  |
|  |  |  |  |  |  | PFDoA | 13.63 | 3.24-94.88 |  |  |
| Li et al. (2022)(53) | China | PCC | 373/657 | serum | Median in cases versus median in referents | PFOA | 3.32 | 2.32-4.75 | 9 | 0 |
|  |  |  |  |  |  | PFDA | 2.22 | 1.55-3.17 |  |  |
|  |  |  |  |  |  | PFTrDA | 0.03 | 0.02-0.06 |  |  |
| Feng et al. (2022)(54) | China | Nested | 226/997 | serum | Highest quartile versus lowest | PFOA | 1.91 | 1.17-3.13 | 9 | 0 |
|  |  |  |  |  |  | PFNA | 1.36 | 0.86-2.16 |  |  |
|  |  |  |  |  |  | PFDA | 1.02 | 0.63-1.64 |  |  |
|  |  |  |  |  |  | PFHpA | 1.24 | 0.78-1.95 |  |  |
|  |  |  |  |  |  | PFOS | 0.86 | 0.53-1.41 |  |  |
|  |  |  |  |  |  | PFHxS | 0.78 | 0.48-1.26 |  |  |
| Itoh et al. (2021)(55) | Japan | HCC | 405/405 | serum | Highest quartile versus lowest | PFOS | 0.15 | 0.06-0.39 | 10 | 0 |
|  |  |  |  |  |  | PFOA | 0.20 | 0.08-0.52 |  |  |
|  |  |  |  |  |  | PFNA | 0.12 | 0.05-0.32 |  |  |
|  |  |  |  |  |  | PFDA | 0.18 | 0.07-0.47 |  |  |
|  |  |  |  |  |  | PFUnDA | 0.37 | 0.15-0.92 |  |  |
|  |  |  |  |  |  | PFDoDA | 0.37 | 0.16-0.84 |  |  |
|  |  |  |  |  |  | PFTrDA | 0.14 | 0.05-0.35 |  |  |
| Tsai et al. (2020)(56) | China | PCC | 120/119 | serum | Median in cases versus median in referents | PFHxS^a^ | 1.59 | 0.99-2.57 | 9 | 0 |
|  |  |  |  |  |  | PFOA^a^ | 1.14 | 0.66-1.96 |  |  |
|  |  |  |  |  |  | PFOS^a^ | 2.34 | 1.02-5.38 |  |  |
|  |  |  |  |  |  | PFNA^a^ | 1.40 | 0.63-3.12 |  |  |
|  |  |  |  |  |  | PFDA^a^ | 1.28 | 0.79-2.08 |  |  |
|  |  |  |  |  |  | PFUnDA^a^ | 1.66 | 0.85-3.24 |  |  |
|  |  |  |  |  |  | PFDoDA^a^ | 0.76 | 0.45-1.28 |  |  |
|  |  |  |  |  |  | PFTrDA^a^ | 1.37 | 0.86-2.20 |  |  |
|  |  |  |  |  |  | PFHxS^b^ | 0.92 | 0.56-1.51 |  |  |
|  |  |  |  |  |  | PFOA^b^ | 0.78 | 0.40-1.51 |  |  |
|  |  |  |  |  |  | PFOS^b^ | 0.62 | 0.29-1.29 |  |  |
|  |  |  |  |  |  | PFNA^b^ | 0.56 | 0.26-1.21 |  |  |
|  |  |  |  |  |  | PFDA^b^ | 0.85 | 0.55-1.32 |  |  |
|  |  |  |  |  |  | PFUnDA^b^ | 0.85 | 0.55-1.32 |  |  |
|  |  |  |  |  |  | PFDoDA^b^ | 0.78 | 0.48-1.25 |  |  |
|  |  |  |  |  |  | PFTrDA^b^ | 0.77 | 0.49-1.21 |  |  |
| Mancini et al. (2020)(57) | French | Nested | 194/194 | serum | Highest quartile versus lowest | PFOS | 1.72 | 0.88-3.36 | 8 | 0 |
|  |  |  |  |  |  | PFOA | 0.92 | 0.43-1.98 |  |  |
| Hurley et al. (2018)(58) | California | Nested | 902/858 | serum | Highest tertile versus lowest | PFOA | 0.93 | 0.72-1.20 | 9 | 0 |
|  |  |  |  |  |  | PFNA | 1.04 | 0.80-1.35 |  |  |
|  |  |  |  |  |  | PFUnDA | 0.79 | 0.61-1.02 |  |  |
|  |  |  |  |  |  | PFHxS | 0.80 | 0.62-1.04 |  |  |
|  |  |  |  |  |  | PFOS | 0.90 | 0.70-1.16 |  |  |
|  |  |  |  |  |  | MeFOSAA | 0.88 | 0.68-1.13 |  |  |
| Wielsøe et al. (2018)(7) | Denmark | HCC | 77/84 | serum | Highest tertile versus lowest | PFHpA | 1.52 | 0.54-4.24 | 9 | 0 |
|  |  |  |  |  |  | PFOA | 2.64 | 1.17-5.97 |  |  |
|  |  |  |  |  |  | PFNA | 2.07 | 0.90-4.76 |  |  |
|  |  |  |  |  |  | PFDA | 2.36 | 1.04-5.36 |  |  |
|  |  |  |  |  |  | PFUnA | 2.00 | 0.88-4.53 |  |  |
|  |  |  |  |  |  | PFDoA | 0.93 | 0.45-1.91 |  |  |
|  |  |  |  |  |  | PFHxS | 2.69 | 1.23-5.88 |  |  |
|  |  |  |  |  |  | PFOS | 5.50 | 1.19-13.84 |  |  |
| Bonefeld-Jørgenser et al. (2014)(59) | Denmark | Nested | 250/233 | serum | Highest tertile versus lowest | PFOS | 0.90 | 0.47-1.70 | 9 | 0 |
|  |  |  |  |  |  | PFOA | 0.94 | 0.51-1.76 |  |  |
|  |  |  |  |  |  | PFNA | 0.80 | 0.43-1.47 |  |  |
|  |  |  |  |  |  | PFHxS | 0.61 | 0.33-1.12 |  |  |
|  |  |  |  |  |  | PFOSA | 1.89 | 1.01-3.54 |  |  |
| Bonefeld-Jorgensen et al. (2011)(60) | Denmark | HCC | 31/115 | serum | Median in cases versus median in referents | PFOS | 1.03 | 1.00-1.07 | 9 | 1 |
|  |  |  |  |  |  | PFOA | 1.20 | 0.77-1.88 |  |  |

a: age ≤ 50 years; b: age > 50 years

Abbreviations: HCC, hospital-based case-control; PCC, population-based case-control; OR, odds ratio; 95%CI, 95% confidence interval; CR: Completeness of reporting; PFDA, perfluorodecanoic acid; PFDoDA, perfluorododecanoic acid; PFHpA, perfluoro heptanoic acid; PFHxS , perfluorohexanesulfonic acid ; PFNA, perfluorononanoic acid; PFOA, perfluorooctanoic acid; PFOS, perfluorooctanesulfonic acid; PFTrDA, perfluoro-n-tridecanoic acid ; PFUnDA, perfluoro undecanoic acid.

| **Supplementary Table 9**: Summary of papers for PBDE exposure and breast cancer risk | | | | | | | | | | |
| --- | --- | --- | --- | --- | --- | --- | --- | --- | --- | --- |
| Reference | Location | Study design | N Cases/  Referents | Biospecimens | Exposure contrast | Substance | OR | 95%CI | CR | Bias |
| Hurley et al. (2019)(61) | california | Nested | 902/936 | serum | Highest quartile versus lowest | BDE-47 | 0.88 | 0.67-1.17 | 9 | 0 |
|  |  |  |  |  |  | BDE-100 | 0.89 | 0.67-1.17 |  |  |
|  |  |  |  |  |  | BDE-153 | 0.89 | 0.67-1.18 |  |  |
| He et al. (2018)(62) | China | HCC | 209/165 | Adipose tissue | Highest tertile versus lowest | BDE-28 | 2.83 | 1.63-4.92 | 11 | 0 |
|  |  |  |  |  |  | BDE-47 | 5.47 | 2.96-10.11 |  |  |
|  |  |  |  |  |  | BDE-71 | 0.38 | 0.22-0.65 |  |  |
|  |  |  |  |  |  | BDE-99 | 3.22 | 1.85-5.60 |  |  |
|  |  |  |  |  |  | BDE-100 | 5.45 | 2.90-10.23 |  |  |
|  |  |  |  |  |  | BDE-138 | 2.40 | 1.37-4.20 |  |  |
|  |  |  |  |  |  | BDE-153 | 1.74 | 1.02-2.97 |  |  |
|  |  |  |  |  |  | BDE-154 | 1.84 | 1.05-3.22 |  |  |
|  |  |  |  |  |  | BDE-183 | 0.73 | 0.41-1.29 |  |  |
|  |  |  |  |  |  | BDE-190 | 1.07 | 0.62-1.85 |  |  |
|  |  |  |  |  |  | BDE-209 | 4.72 | 2.52-8.83 |  |  |
|  |  |  |  |  |  | ∑PBDEs | 1.83 | 1.07-3.14 |  |  |
| Holmes et al. (2014)(10) | Alaska | HCC | 75/95 | serum | Median in cases versus median in referents | BDE-47 | 1.58 | 0.75-3.33 | 8 | 1 |
| Hurley et al. (2011)(63) | America | HCC | 78/56 | Adipose tissue | Highest tertile versus lowest | BDE-47 | 0.56 | 0.19-1.68 | 8 | 0 |
|  |  |  |  |  |  | BDE-99 | 1.19 | 0.35-4.10 |  |  |
|  |  |  |  |  |  | BDE-100 | 0.91 | 0.33-2.53 |  |  |
|  |  |  |  |  |  | BDE-153 | 0.52 | 0.19-1.39 |  |  |
|  |  |  |  |  |  | BDE-154 | 1.67 | 0.44-6.29 |  |  |
|  |  |  |  |  |  | ∑PBDEs | 2.04 | 0.45-9.20 |  |  |

Abbreviations: HCC, hospital-based case-control; PCC, population-based case-control; OR, odds ratio; 95%CI, 95% confidence interval; CR: Completeness of reporting; PBDE, polybrominated diphenyl ethers.

| **Supplementary Table 10**: Summary of papers for PBA exposure and breast cancer risk | | | | | | | | | | |
| --- | --- | --- | --- | --- | --- | --- | --- | --- | --- | --- |
| Reference | Location | Study design | N Cases/  Referents | Biospecimens | Exposure contrast | Substance | OR | 95%CI | CR | Bias |
| Wu et al. (2022)(48) | America | Nested | 1030/1032 | urine | Highest tertile versus lowest | BPA | 0.95 | 0.75-1.21 | 9 | 0 |
| Parada et al. (2019)(64) | America | Nested | 711/598 | urine | Highest tertile versus lowest | BPA | 0.75 | 0.52-1.08 | 8 | 0 |
| Reeves et al. (2018)(65) | America | HCC | 36/14 | Adipose tissue | Highest quartile versus lowest | BPA | 0.90 | 0.40-2.00 | 9 | 0 |
| Trabert et al. (2014)(66) | Poland | PCC | 575/575 | urine | Highest quartile versus lowest | BPA | 1.09 | 0.73-1.63 | 10 | 0 |
| Aschengrau et al. (1998)(67) | America | PCC | 261/753 | urine | Median in cases versus median in referents | BPA | 0.80 | 0.50-1.40 | 9 | 0 |

Abbreviations: HCC, hospital-based case-control; PCC, population-based case-control; OR, odds ratio; 95%CI, 95% confidence interval; CR: Completeness of reporting; PBA, bisphenol A.

**Supplementary Table 11. Evaluation form (rating form) for bias and completeness of reporting:**

A. Completeness of reporting

B. Assessment of bias and confounding

| **A. Completeness of reporting (is adequate information provided or not? No assessment of quality)**  Each issue is rated with 1 (adequate information) or 0 (not enough description), if 0 indicate briefly the main reason | | |
| --- | --- | --- |
| Study design | |  |
| Sampling frame and procedures | |  |
| Inclusion and exclusion criteria | |  |
| Population characteristics of exposed/unexposed or cases/referents | |  |
| Response rates reported or implicitly given | |  |
| Methods for exposure measurements (reference to method for chemical analysis or detailed description)? | |  |
| Methods for outcome ascertainment | |  |
| External quality assurance program of biochemical analyses (certified lab and/or participating in analyses of spiked samples from other labs)? | |  |
| Detection level and precision (CV) (information provided on both issues)? | |  |
| Statistical analysis | |  |
| Exposure-response (provided?) | |  |
| Completeness of reporting sum score (0-11) | |  |
| **B. Assessment of bias and confounding** | | |
|  | Check | **High risk (0)** (likely risk of bias not addressed)  **Uncertain risk (1)** (information not provided)  If high risk: Justify your decision by short statements or quotes from the study  **Low risk** (2)(best practice) |
| 1-Reporting of tested hypotheses |  | High risk: incomplete/selective reporting of tested hypotheses compared to objectives or available data |
|  |  | Low risk: Estimates presented for all hypotheses |
| 2-Sample size justification (power calculations and/or addressing sample size in discussion) |  | High risk: small numbers may increase risk of false negative reporting |
|  |  | Low risk: Justification provided by authors |
| 3-Selection bias |  |  |
| Selection bias cohort studies (attrition). Nested-case referent studies evaluated according to this item, non-response of cases and controls also taken into consideration) |  | High risk: loss to follow-up larger than 20% or differ more than 10% between exposed and unexposed |
|  |  | Low risk: Loss to follow-up less than 20% with no difference between groups |
|  |  | Uncertain: attrition not reported |
| Selection bias case-control studies (non-response) |  | High risk: Non-response more than 20% or non-response differed by more than 10% in cases and controls |
|  |  | Low risk: Non-response less than 20% with no difference between cases and controls |
|  |  | Uncertain: Non-response not reported |
| 4-Information bias (outcome ascertainment) |  | High risk: outcome identified by patient recall in questionnaires or interviews |
|  |  | Low risk: outcome identified by medical examination/record or from validated registries |
|  |  | Uncertain: methods not described |
| 5-Confounding (are relevant factors considered? judgement specific for outcome). |  | High risk: Major confounding factors/effect modifiers not or partially assessed. (for example: mothers pre-pregnancy BMI, mothers own age at puberty, history of precocious puberty or late puberty, family SES (income and educational status), race, ethnicity, child’s own BMI, physical activity), breast fed and history of endocrine disease. |
|  |  | Low risk: Major confounding factors/effect modifiers adequately accounted for by design and/or analysis |
|  |  | Uncertain: adjustment for confounding factors not reported |
| 6-Measuring of confounding factors |  | High risk: Non valid or inadequate reporting or measures of confounding factors |
|  |  | Low risk: Adequate and non-differential ascertainment of information |
|  |  | Uncertain risk: Source and methods for collection of data not reported |
| 7-Exposure-contrast |  | High risk: exposure categories split by the median or by ad hoc grouping  Comparison of median values in cases and control do not allow for evaluation of exposure-response |
|  |  | Low risk: exposure categories divided by tertiles, quantiles (or more detailed) or by grouping of levels |
| Sum score bias(confounding (range 0-14) |  |  |

**Supplementary Table 12: The summary of the potential sources**

| StudyID | Reporting of tested hypotheses | Sample size justication | Selection bias | Information bias (outcome ascertainment) | Confounding (relevant confounders considered) | Measuring of confounding factors | Expoure contrast |
| --- | --- | --- | --- | --- | --- | --- | --- |
| Mekonen et al. (2021)(1) | low risk | high risk | uncertain | low risk | low risk | low risk | high risk |
| Miao et al. (2021)(2) | low risk | low risk | uncertain | low risk | low risk | low risk | low risk |
| Bachelet et al. (2019) (3) | low risk | low risk | uncertain | low risk | low risk | low risk | low risk |
| Cohn et al. (2019)(4) | low risk | high risk | uncertain | low risk | low risk | low risk | low risk |
| Huang et al. (2019)(5) | low risk | low risk | low risk | low risk | low risk | low risk | low risk |
| Kaur et al. (2019)(6) | low risk | high risk | low risk | low risk | low risk | high risk | low risk |
| Wielsøe et al. (2018)(7) | low risk | high risk | uncertain | low risk | low risk | low risk | low risk |
| Pastor-Barriuso et al. (2016)(8) | low risk | low risk | high risk | low risk | low risk | low risk | low risk |
| Arrebola et al. (2015)(9) | low risk | low risk | high risk | low risk | low risk | low risk | low risk |
| Holmes et al. (2014)(10) | low risk | high risk | uncertain | low risk | low risk | low risk | high risk |
| Tang et al. (2014)(11) | low risk | high risk | low risk | low risk | low risk | low risk | high risk |
| Boada et al. (2012)(12) | low risk | low risk | low risk | low risk | low risk | low risk | high risk |
| Itoh et al. (2009)(13) | low risk | low risk | low risk | low risk | low risk | low risk | low risk |
| Iwasaki et al. (2008)(14) | low risk | low risk | low risk | low risk | low risk | low risk | low risk |
| Cohn et al. (2007)(15) | low risk | low risk | uncertain | low risk | low risk | low risk | low risk |
| Gatto et al. (2007)(16) | low risk | low risk | high risk | low risk | low risk | low risk | low risk |
| Raaschou-Nielsen et al. (2005)(17) | low risk | low risk | uncertain | low risk | low risk | low risk | low risk |
| Charlier et al. (2004)(18) | low risk | low risk | low risk | low risk | low risk | low risk | low risk |
| McCready et al. (2004)(19) | low risk | high risk | uncertain | low risk | low risk | high risk | low risk |
| Pavuk et al. (2003)(20) | low risk | high risk | uncertain | low risk | low risk | low risk | low risk |
| Gammon et al. (2002)(21) | low risk | low risk | low risk | low risk | low risk | low risk | low risk |
| Laden et al. (2001)(22) | low risk | low risk | low risk | low risk | low risk | low risk | low risk |
| Aronson et al. (2000)(23) | low risk | low risk | low risk | low risk | low risk | low risk | low risk |
| Bagga et al. (2000)(24) | low risk | high risk | uncertain | low risk | high risk | high risk | high risk |
| Demers et al. (2000)(25) | low risk | low risk | uncertain | low risk | low risk | low risk | low risk |
| Høyer et al. (2000)(26) | low risk | low risk | high risk | low risk | low risk | low risk | low risk |
| Millikan et al. (2000)(27) | low risk | low risk | uncertain | low risk | low risk | low risk | low risk |
| Romieu et al. (2000)(28) | low risk | high risk | uncertain | low risk | low risk | low risk | low risk |
| Stellman et al. (2000)(29) | low risk | low risk | low risk | low risk | low risk | low risk | low risk |
| Wolff et al. (2000)(30) | low risk | high risk | low risk | low risk | low risk | low risk | low risk |
| Dorgan et al. (1999)(31) | low risk | low risk | low risk | low risk | low risk | low risk | high risk |
| Mendonça et al. (1999)(32) | low risk | low risk | uncertain | low risk | low risk | low risk | high risk |
| Høyer et al. (1998)(33) | low risk | high risk | uncertain | low risk | low risk | low risk | low risk |
| López-Carrillo et al. (1997)(34) | low risk | high risk | low risk | low risk | low risk | low risk | low risk |
| Schecter et al. (1997)(35) | low risk | high risk | uncertain | low risk | high risk | high risk | low risk |
| Wolf( et al. (1993)(36) | high risk | low risk | uncertain | low risk | low risk | high risk | low risk |
| López-Carrillo et al. (2002)(37) | low risk | high risk | low risk | low risk | low risk | low risk | low risk |
| Moysich et al. (1998)(38) | low risk | low risk | high risk | low risk | low risk | low risk | low risk |
| Xu et al. (2010)(39) | low risk | low risk | low risk | low risk | low risk | low risk | low risk |
| Parada et al. (2021)(40) | low risk | low risk | low risk | low risk | low risk | low risk | low risk |
| Cohn et al. (2012)(41) | low risk | low risk | low risk | low risk | low risk | low risk | low risk |
| Recio-Vega et al. (2011)(42) | low risk | high risk | low risk | low risk | low risk | low risk | high risk |
| Charlier et al. (2004)(43) | low risk | low risk | low risk | low risk | low risk | low risk | low risk |
| Demers et al. (2000)(44) | low risk | low risk | uncertain | low risk | low risk | low risk | low risk |
| Holford et al. (2000)(45) | low risk | low risk | uncertain | low risk | low risk | low risk | low risk |
| Zheng et al. (2000)(46) | low risk | low risk | low risk | low risk | low risk | low risk | high risk |
| Mukherjee et al. (2022)(47) | low risk | high risk | uncertain | low risk | low risk | low risk | high risk |
| Wu et al. (2021)(48) | low risk | low risk | uncertain | low risk | low risk | low risk | low risk |
| Reeves et al. (2019)(49) | low risk | low risk | uncertain | low risk | low risk | low risk | low risk |
| Parada et al. (2018)(50) | low risk | low risk | uncertain | low risk | low risk | low risk | low risk |
| Chang et al. (2023)(51) | low risk | low risk | low risk | low risk | low risk | low risk | low risk |
| Velarde et al. (2022)(52) | low risk | high risk | uncertain | uncertain | low risk | low risk | low risk |
| Li et al. (2022)(53) | low risk | low risk | uncertain | uncertain | uncertain | uncertain | high risk |
| Feng et al. (2022)(54) | low risk | low risk | low risk | low risk | low risk | low risk | low risk |
| Itoh et al. (2021)(55) | low risk | low risk | uncertain | low risk | low risk | low risk | low risk |
| Tsai et al. (2020)(56) | low risk | low risk | low risk | low risk | low risk | low risk | high risk |
| Mancini et al. (2020)(57) | low risk | low risk | uncertain | low risk | low risk | low risk | low risk |
| Hurley et al. (2018)(58) | low risk | low risk | low risk | low risk | low risk | low risk | low risk |
| Bonefeld-Jørgenser et al. (2014)(59) | low risk | low risk | low risk | low risk | low risk | low risk | low risk |
| Bonefeld-Jorgensen et al. (2011)(60) | low risk | high risk | uncertain | low risk | low risk | low risk | high risk |
| Hurley et al. (2019)(61) | low risk | low risk | low risk | low risk | low risk | low risk | low risk |
| He et al. (2018)(62) | low risk | low risk | low risk | low risk | low risk | low risk | low risk |
| Hurley et al. (2011)(63) | low risk | high risk | low risk | low risk | low risk | low risk | low risk |
| Parada et al. (2019)(64) | low risk | low risk | uncertain | low risk | low risk | low risk | low risk |
| Reeves et al. (2018)(65) | low risk | low risk | uncertain | low risk | low risk | low risk | low risk |
| Trabert et al. (2014)(66) | low risk | low risk | low risk | low risk | low risk | low risk | low risk |
| Aschengrau et al. (1998)(67) | low risk | low risk | uncertain | low risk | low risk | low risk | high risk |

1. Mekonen S, Ibrahim M, Astatkie H, Abreha A. Exposure to organochlorine pesticides as a predictor to breast cancer: A case-control study among Ethiopian women. PLoS One 2021;16:e0257704.10.1371/journal.pone.0257704

2. Miao Y, Rong M, Li M, He H, Zhang L, Zhang S, et al.. Serum concentrations of organochlorine pesticides, biomarkers of oxidative stress, and risk of breast cancer. Environ Pollut 2021;286:117386.10.1016/j.envpol.2021.117386

3. Bachelet D, Verner MA, Neri M, Cordina Duverger É, Charlier C, Arveux P, et al.. Breast Cancer and Exposure to Organochlorines in the CECILE Study: Associations with Plasma Levels Measured at the Time of Diagnosis and Estimated during Adolescence. Int J Environ Res Public Health 2019;16.10.3390/ijerph16020271

4. Cohn BA, Cirillo PM, Terry MB. DDT and Breast Cancer: Prospective Study of Induction Time and Susceptibility Windows. J Natl Cancer Inst 2019;111:803-810.10.1093/jnci/djy198

5. Huang W, He Y, Xiao J, Huang Y, Li A, He M, et al.. Risk of breast cancer and adipose tissue concentrations of polychlorinated biphenyls and organochlorine pesticides: a hospital-based case-control study in Chinese women. Environ Sci Pollut Res Int 2019;26:32128-32136.10.1007/s11356-019-06404-3

6. Kaur N, Swain SK, Banerjee BD, Sharma T, Krishnalata T. Organochlorine pesticide exposure as a risk factor for breast cancer in young Indian women: A case-control study. South Asian J Cancer 2019;8:212-214.10.4103/sajc.sajc_427_18

7. Wielsøe M, Kern P, Bonefeld-Jørgensen EC. Serum levels of environmental pollutants is a risk factor for breast cancer in Inuit: a case control study. Environ Health 2017;16:56.10.1186/s12940-017-0269-6

8. Pastor-Barriuso R, Fernández MF, Castaño-Vinyals G, Whelan D, Pérez-Gómez B, Llorca J, et al.. Total Effective Xenoestrogen Burden in Serum Samples and Risk for Breast Cancer in a Population-Based Multicase-Control Study in Spain. Environ Health Perspect 2016;124:1575-1582.10.1289/ehp157

9. Arrebola JP, Belhassen H, Artacho-Cordón F, Ghali R, Ghorbel H, Boussen H, et al.. Risk of female breast cancer and serum concentrations of organochlorine pesticides and polychlorinated biphenyls: a case-control study in Tunisia. Sci Total Environ 2015;520:106-113.10.1016/j.scitotenv.2015.03.045

10. Holmes AK, Koller KR, Kieszak SM, Sjodin A, Calafat AM, Sacco FD, et al.. Case-control study of breast cancer and exposure to synthetic environmental chemicals among Alaska Native women. Int J Circumpolar Health 2014;73:25760.10.3402/ijch.v73.25760

11. Tang M, Zhao M, Zhou S, Chen K, Zhang C, Liu W. Assessing the underlying breast cancer risk of Chinese females contributed by dietary intake of residual DDT from agricultural soils. Environ Int 2014;73:208-215.10.1016/j.envint.2014.08.001

12. Boada LD, Zumbado M, Henríquez-Hernández LA, Almeida-González M, Alvarez-León EE, Serra-Majem L, et al.. Complex organochlorine pesticide mixtures as determinant factor for breast cancer risk: a population-based case-control study in the Canary Islands (Spain). Environ Health 2012;11:28.10.1186/1476-069x-11-28

13. Itoh H, Iwasaki M, Hanaoka T, Kasuga Y, Yokoyama S, Onuma H, et al.. Serum organochlorines and breast cancer risk in Japanese women: a case-control study. Cancer Causes Control 2009;20:567-580.10.1007/s10552-008-9265-z

14. Iwasaki M, Inoue M, Sasazuki S, Kurahashi N, Itoh H, Usuda M, et al.. Plasma organochlorine levels and subsequent risk of breast cancer among Japanese women: a nested case-control study. Sci Total Environ 2008;402:176-183.10.1016/j.scitotenv.2008.05.009

15. Cohn BA, Wolff MS, Cirillo PM, Sholtz RI. DDT and breast cancer in young women: new data on the significance of age at exposure. Environ Health Perspect 2007;115:1406-1414.10.1289/ehp.10260

16. Gatto NM, Longnecker MP, Press MF, Sullivan-Halley J, McKean-Cowdin R, Bernstein L. Serum organochlorines and breast cancer: a case-control study among African-American women. Cancer Causes Control 2007;18:29-39.10.1007/s10552-006-0070-2

17. Raaschou-Nielsen O, Pavuk M, Leblanc A, Dumas P, Philippe Weber J, Olsen A, et al.. Adipose organochlorine concentrations and risk of breast cancer among postmenopausal Danish women. Cancer Epidemiol Biomarkers Prev 2005;14:67-74

18. Charlier C, Foidart JM, Pitance F, Herman P, Gaspard U, Meurisse M, et al.. Environmental dichlorodiphenyltrichlorethane or hexachlorobenzene exposure and breast cancer: is there a risk? Clin Chem Lab Med 2004;42:222-227.10.1515/cclm.2004.040

19. McCready D, Aronson KJ, Chu W, Fan W, Vesprini D, Narod SA. Breast tissue organochlorine levels and metabolic genotypes in relation to breast cancer risk Canada. Cancer Causes Control 2004;15:399-418.10.1023/B:CACO.0000027505.32564.c2

20. Pavuk M, Cerhan JR, Lynch CF, Kocan A, Petrik J, Chovancova J. Case-control study of PCBs, other organochlorines and breast cancer in Eastern Slovakia. J Expo Anal Environ Epidemiol 2003;13:267-275.10.1038/sj.jea.7500277

21. Gammon MD, Wolff MS, Neugut AI, Eng SM, Teitelbaum SL, Britton JA, et al.. Environmental toxins and breast cancer on Long Island. II. Organochlorine compound levels in blood. Cancer Epidemiol Biomarkers Prev 2002;11:686-697

22. Laden F, Hankinson SE, Wolff MS, Colditz GA, Willett WC, Speizer FE, et al.. Plasma organochlorine levels and the risk of breast cancer: an extended follow-up in the Nurses' Health Study. Int J Cancer 2001;91:568-574.10.1002/1097-0215(200002)9999:9999<::aid-ijc1081>3.0.co;2-w

23. Aronson KJ, Miller AB, Woolcott CG, Sterns EE, McCready DR, Lickley LA, et al.. Breast adipose tissue concentrations of polychlorinated biphenyls and other organochlorines and breast cancer risk. Cancer Epidemiol Biomarkers Prev 2000;9:55-63

24. Bagga D, Anders KH, Wang HJ, Roberts E, Glaspy JA. Organochlorine pesticide content of breast adipose tissue from women with breast cancer and control subjects. J Natl Cancer Inst 2000;92:750-753.10.1093/jnci/92.9.750

25. Demers A, Ayotte P, Brisson J, Dodin S, Robert J, Dewailly E. Risk and aggressiveness of breast cancer in relation to plasma organochlorine concentrations. Cancer Epidemiol Biomarkers Prev 2000;9:161-166

26. Høyer AP, Jørgensen T, Grandjean P, Hartvig HB. Repeated measurements of organochlorine exposure and breast cancer risk (Denmark). Cancer Causes Control 2000;11:177-184.10.1023/a:1008926219539

27. Millikan R, DeVoto E, Duell EJ, Tse CK, Savitz DA, Beach J, et al.. Dichlorodiphenyldichloroethene, polychlorinated biphenyls, and breast cancer among African-American and white women in North Carolina. Cancer Epidemiol Biomarkers Prev 2000;9:1233-1240

28. Romieu I, Hernandez-Avila M, Lazcano-Ponce E, Weber JP, Dewailly E. Breast cancer, lactation history, and serum organochlorines. Am J Epidemiol 2000;152:363-370.10.1093/aje/152.4.363

29. Stellman SD, Djordjevic MV, Britton JA, Muscat JE, Citron ML, Kemeny M, et al.. Breast cancer risk in relation to adipose concentrations of organochlorine pesticides and polychlorinated biphenyls in Long Island, New York. Cancer Epidemiol Biomarkers Prev 2000;9:1241-1249

30. Wolff MS, Berkowitz GS, Brower S, Senie R, Bleiweiss IJ, Tartter P, et al.. Organochlorine exposures and breast cancer risk in New York City women. Environ Res 2000;84:151-161.10.1006/enrs.2000.4075

31. Dorgan JF, Brock JW, Rothman N, Needham LL, Miller R, Stephenson HE, Jr., et al.. Serum organochlorine pesticides and PCBs and breast cancer risk: results from a prospective analysis (USA). Cancer Causes Control 1999;10:1-11.10.1023/a:1008824131727

32. Mendonça GA, Eluf-Neto J, Andrada-Serpa MJ, Carmo PA, Barreto HH, Inomata ON, et al.. Organochlorines and breast cancer: a case-control study in Brazil. Int J Cancer 1999;83:596-600.10.1002/(sici)1097-0215(19991126)83:5<596::aid-ijc4>3.0.co;2-p

33. Høyer AP, Grandjean P, Jørgensen T, Brock JW, Hartvig HB. Organochlorine exposure and risk of breast cancer. Lancet 1998;352:1816-1820.10.1016/s0140-6736(98)04504-8

34. López-Carrillo L, Blair A, López-Cervantes M, Cebrián M, Rueda C, Reyes R, et al.. Dichlorodiphenyltrichloroethane serum levels and breast cancer risk: a case-control study from Mexico. Cancer Res 1997;57:3728-3732

35. Schecter A, Toniolo P, Dai LC, Thuy LT, Wolff MS. Blood levels of DDT and breast cancer risk among women living in the north of Vietnam. Arch Environ Contam Toxicol 1997;33:453-456.10.1007/s002449900276

36. Wolff MS, Toniolo PG, Lee EW, Rivera M, Dubin N. Blood levels of organochlorine residues and risk of breast cancer. J Natl Cancer Inst 1993;85:648-652.10.1093/jnci/85.8.648

37. López-Carrillo L, López-Cervantes M, Torres-Sánchez L, Blair A, Cebrián ME, García RM. Serum levels of beta-hexachlorocyclohexane, hexachlorobenzene and polychlorinated biphenyls and breast cancer in Mexican women. Eur J Cancer Prev 2002;11:129-135.10.1097/00008469-200204000-00004

38. Moysich KB, Ambrosone CB, Vena JE, Shields PG, Mendola P, Kostyniak P, et al.. Environmental organochlorine exposure and postmenopausal breast cancer risk. Cancer Epidemiol Biomarkers Prev 1998;7:181-188

39. Xu X, Dailey AB, Talbott EO, Ilacqua VA, Kearney G, Asal NR. Associations of serum concentrations of organochlorine pesticides with breast cancer and prostate cancer in U.S. adults. Environ Health Perspect 2010;118:60-66.10.1289/ehp.0900919

40. Parada H, Jr., Benmarhnia T, Engel LS, Sun X, Tse CK, Hoh E, et al.. A Congener-specific and Mixture Analysis of Plasma Polychlorinated Biphenyl Levels and Incident Breast Cancer. Epidemiology 2021;32:499-507.10.1097/ede.0000000000001356

41. Cohn BA, Terry MB, Plumb M, Cirillo PM. Exposure to polychlorinated biphenyl (PCB) congeners measured shortly after giving birth and subsequent risk of maternal breast cancer before age 50. Breast Cancer Res Treat 2012;136:267-275.10.1007/s10549-012-2257-4

42. Recio-Vega R, Velazco-Rodriguez V, Ocampo-Gómez G, Hernandez-Gonzalez S, Ruiz-Flores P, Lopez-Marquez F. Serum levels of polychlorinated biphenyls in Mexican women and breast cancer risk. J Appl Toxicol 2011;31:270-278.10.1002/jat.1672

43. Charlier CJ, Albert AI, Zhang L, Dubois NG, Plomteux GJ. Polychlorinated biphenyls contamination in women with breast cancer. Clin Chim Acta 2004;347:177-181.10.1016/j.cccn.2004.04.025

44. Demers A, Ayotte P, Brisson J, Dodin S, Robert J, Dewailly E. Plasma concentrations of polychlorinated biphenyls and the risk of breast cancer: a congener-specific analysis. Am J Epidemiol 2002;155:629-635.10.1093/aje/155.7.629

45. Holford TR, Zheng T, Mayne ST, Zahm SH, Tessari JD, Boyle P. Joint effects of nine polychlorinated biphenyl (PCB) congeners on breast cancer risk. Int J Epidemiol 2000;29:975-982.10.1093/ije/29.6.975

46. Zheng T, Holford TR, Tessari J, Mayne ST, Owens PH, Ward B, et al.. Breast cancer risk associated with congeners of polychlorinated biphenyls. Am J Epidemiol 2000;152:50-58.10.1093/aje/152.1.50

47. Mukherjee Das A, Gogia A, Garg M, Elaiyaraja A, Arambam P, Mathur S, et al.. Urinary concentration of endocrine-disrupting phthalates and breast cancer risk in Indian women: A case-control study with a focus on mutations in phthalate-responsive genes. Cancer Epidemiol 2022;79:102188.10.1016/j.canep.2022.102188

48. Wu AH, Franke AA, Wilkens LR, Tseng C, Conroy SM, Li Y, et al.. Urinary phthalate exposures and risk of breast cancer: the Multiethnic Cohort study. Breast Cancer Res 2021;23:44.10.1186/s13058-021-01419-6

49. Reeves KW, Díaz Santana M, Manson JE, Hankinson SE, Zoeller RT, Bigelow C, et al.. Urinary Phthalate Biomarker Concentrations and Postmenopausal Breast Cancer Risk. J Natl Cancer Inst 2019;111:1059-1067.10.1093/jnci/djz002

50. Parada H, Jr., Gammon MD, Chen J, Calafat AM, Neugut AI, Santella RM, et al.. Urinary Phthalate Metabolite Concentrations and Breast Cancer Incidence and Survival following Breast Cancer: The Long Island Breast Cancer Study Project. Environ Health Perspect 2018;126:047013.10.1289/ehp2083

51. Chang VC, Rhee J, Berndt SI, Moore SC, Freedman ND, Jones RR, et al.. Serum perfluorooctane sulfonate and perfluorooctanoate and risk of postmenopausal breast cancer according to hormone receptor status: An analysis in the Prostate, Lung, Colorectal and Ovarian Cancer Screening Trial. Int J Cancer 2023.10.1002/ijc.34487

52. Velarde MC, Chan AFO, Sajo M, Zakharevich I, Melamed J, Uy GLB, et al.. Elevated levels of perfluoroalkyl substances in breast cancer patients within the Greater Manila Area. Chemosphere 2022;286:131545.10.1016/j.chemosphere.2021.131545

53. Li X, Song F, Liu X, Shan A, Huang Y, Yang Z, et al.. Perfluoroalkyl substances (PFASs) as risk factors for breast cancer: a case-control study in Chinese population. Environ Health 2022;21:83.10.1186/s12940-022-00895-3

54. Feng Y, Bai Y, Lu Y, Chen M, Fu M, Guan X, et al.. Plasma perfluoroalkyl substance exposure and incidence risk of breast cancer: A case-cohort study in the Dongfeng-Tongji cohort. Environ Pollut 2022;306:119345.10.1016/j.envpol.2022.119345

55. Itoh H, Harada KH, Kasuga Y, Yokoyama S, Onuma H, Nishimura H, et al.. Serum perfluoroalkyl substances and breast cancer risk in Japanese women: A case-control study. Sci Total Environ 2021;800:149316.10.1016/j.scitotenv.2021.149316

56. Tsai MS, Chang SH, Kuo WH, Kuo CH, Li SY, Wang MY, et al.. A case-control study of perfluoroalkyl substances and the risk of breast cancer in Taiwanese women. Environ Int 2020;142:105850.10.1016/j.envint.2020.105850

57. Mancini FR, Cano-Sancho G, Gambaretti J, Marchand P, Boutron-Ruault MC, Severi G, et al.. Perfluorinated alkylated substances serum concentration and breast cancer risk: Evidence from a nested case-control study in the French E3N cohort. Int J Cancer 2020;146:917-928.10.1002/ijc.32357

58. Hurley S, Goldberg D, Wang M, Park JS, Petreas M, Bernstein L, et al.. Breast cancer risk and serum levels of per- and poly-fluoroalkyl substances: a case-control study nested in the California Teachers Study. Environ Health 2018;17:83.10.1186/s12940-018-0426-6

59. Bonefeld-Jørgensen EC, Long M, Fredslund SO, Bossi R, Olsen J. Breast cancer risk after exposure to perfluorinated compounds in Danish women: a case-control study nested in the Danish National Birth Cohort. Cancer Causes Control 2014;25:1439-1448.10.1007/s10552-014-0446-7

60. Bonefeld-Jorgensen EC, Long M, Bossi R, Ayotte P, Asmund G, Krüger T, et al.. Perfluorinated compounds are related to breast cancer risk in Greenlandic Inuit: a case control study. Environ Health 2011;10:88.10.1186/1476-069x-10-88

61. Hurley S, Goldberg D, Park JS, Petreas M, Bernstein L, Anton-Culver H, et al.. A breast cancer case-control study of polybrominated diphenyl ether (PBDE) serum levels among California women. Environ Int 2019;127:412-419.10.1016/j.envint.2019.03.043

62. He Y, Peng L, Zhang W, Liu C, Yang Q, Zheng S, et al.. Adipose tissue levels of polybrominated diphenyl ethers and breast cancer risk in Chinese women: A case-control study. Environ Res 2018;167:160-168.10.1016/j.envres.2018.07.009

63. Hurley S, Reynolds P, Goldberg D, Nelson DO, Jeffrey SS, Petreas M. Adipose levels of polybrominated diphenyl ethers and risk of breast cancer. Breast Cancer Res Treat 2011;129:505-511.10.1007/s10549-011-1481-7

64. Parada H, Jr., Gammon MD, Ettore HL, Chen J, Calafat AM, Neugut AI, et al.. Urinary concentrations of environmental phenols and their associations with breast cancer incidence and mortality following breast cancer. Environ Int 2019;130:104890.10.1016/j.envint.2019.05.084

65. Reeves KW, Schneider S, Xue J, Kannan K, Mason H, Johnson M, et al.. Bisphenol-A in breast adipose tissue of breast cancer cases and controls. Environ Res 2018;167:735-738.10.1016/j.envres.2018.08.033

66. Trabert B, Falk RT, Figueroa JD, Graubard BI, Garcia-Closas M, Lissowska J, et al.. Urinary bisphenol A-glucuronide and postmenopausal breast cancer in Poland. Cancer Causes Control 2014;25:1587-1593.10.1007/s10552-014-0461-8

67. Aschengrau A, Coogan PF, Quinn M, Cashins LJ. Occupational exposure to estrogenic chemicals and the occurrence of breast cancer: an exploratory analysis. Am J Ind Med 1998;34:6-14.10.1002/(sici)1097-0274(199807)34:1<6::aid-ajim2>3.0.co;2-x
